# RBFOX2 deregulation promotes pancreatic cancer progression and metastasis through alternative splicing

**DOI:** 10.1038/s41467-023-44126-w

**Published:** 2023-12-19

**Authors:** Michelle Maurin, Mohammadreza Ranjouri, Cristina Megino-Luque, Justin Y. Newberg, Dongliang Du, Katelyn Martin, Robert E. Miner, Mollie S. Prater, Dave Keng Boon Wee, Barbara Centeno, Shondra M. Pruett-Miller, Paul Stewart, Jason B. Fleming, Xiaoqing Yu, Jose Javier Bravo-Cordero, Ernesto Guccione, Michael A. Black, Karen M. Mann

**Affiliations:** 1 Department of Molecular Oncology, Moffitt Cancer Center, Tampa, FL 33612 USA; 2 Division of Hematology and Oncology, Department of Medicine, The Tisch Cancer Institute, Icahn School of Medicine at Mount Sinai, New York, NY 10029 USA; 3 Department of Biostatistics and Bioinformatics, Moffitt Cancer Center, Tampa, FL 33612 USA; 4 Department of Cell and Molecular Biology and Center for Advanced Genome Engineering (CAGE), St. Jude Children’s Research Hospital, Memphis, TN 38105 USA; 5 Institute for Molecular and Cell Biology (IMCB), Agency for Science, Technology and Research (A*STAR), Singapore, 138673 Republic of Singapore; 6 Department of Anatomic Pathology, Moffitt Cancer Center, Tampa, FL 33612 USA; 7 Department of Gastrointestinal Oncology, Moffitt Cancer Center, Tampa, FL 33612 USA; 8 Center for OncoGenomics and Innovative Therapeutics (COGIT), Icahn School of Medicine at Mount Sinai, New York, NY 10029 USA; 9 Center for Therapeutics Discovery, Department of Oncological Sciences and Pharmacological Sciences, Tisch Cancer Institute, Icahn School of Medicine at Mount Sinai, New York, NY 10029 USA; 10 Department of Biochemistry, University of Otago, Dunedin, 9054 New Zealand

**Keywords:** RNA splicing, Pancreatic cancer, Pancreatic cancer

## Abstract

RNA splicing is an important biological process associated with cancer initiation and progression. However, the contribution of alternative splicing to pancreatic cancer (PDAC) development is not well understood. Here, we identify an enrichment of RNA binding proteins (RBPs) involved in splicing regulation linked to PDAC progression from a forward genetic screen using *Sleeping Beauty* insertional mutagenesis in a mouse model of pancreatic cancer. We demonstrate downregulation of RBFOX2, an RBP of the FOX family, promotes pancreatic cancer progression and liver metastasis. Specifically, we show RBFOX2 regulates exon splicing events in transcripts encoding proteins involved in cytoskeletal remodeling programs. These exons are differentially spliced in PDAC patients, with enhanced exon skipping in the classical subtype for several RBFOX2 targets. RBFOX2 mediated splicing of *ABI1*, encoding the Abelson-interactor 1 adapter protein, controls the abundance and localization of ABI1 protein isoforms in pancreatic cancer cells and promotes the relocalization of ABI1 from the cytoplasm to the periphery of migrating cells. Using splice-switching antisense oligonucleotides (AONs) we demonstrate the *ABI1* ∆Ex9 isoform enhances cell migration. Together, our data identify a role for RBFOX2 in promoting PDAC progression through alternative splicing regulation.

## Abstract (web summary)

The role of alternative splicing in pancreatic cancer (PDAC) development remains to be explored. Here, RBFOX2 is shown to regulate exon splicing events in transcripts encoding proteins involved in cytoskeletal remodelling programs and its downregulation promotes PDAC progression and liver metastasis.

## Introduction

Pancreatic ductal adenocarcinoma (PDAC) is a highly metastatic cancer driven by oncogenic *KRAS* mutations and inactivation of tumor suppressor genes *TP53*, *SMAD4,* and *CDKN2A*. Importantly, these driver mutations are present in liver metastases^1,2^, the major site of disease dissemination and recurrence^3–5^, suggesting these events are necessary for disease maintenance. However, additional signaling, metabolic and regulatory processes contribute to disease progression. Recently, the regulation of RNA splicing has gained attention for its importance in cancer development, and initial analyses of splicing events in cancer have elucidated differential splicing signatures between tumor and normal tissue^6–8^, conserved splicing events across cancer types^9,10^, and the influence of alternative splicing events on therapy response.

RNA splicing is an integral biological process that regulates transcript stability and extends the repertoire of transcripts generated from a single genetic locus, contributing to a diversity of protein functions. Alternative splicing plays a major role in producing cell-type specific transcripts and is important for both maintaining embryonic stem cell (ESC) pluripotency and promoting cellular differentiation^11–14^. Regulation of alternative splicing is complex, involving conserved protein complexes inclusive of RNA binding proteins that direct the specificity of splicing events. In pancreatic cancer, individual splicing events have been linked to disease progression, patient survival, and response to chemotherapy^15–18^. Analysis of global RNA splicing signatures in pancreatic cancer using TCGA RNA-seq data identified enrichment of alternatively spliced transcripts in metabolic processes, cell-cell adhesion and cytoskeletal organization, suggesting alternative splicing in pancreatic cancer may impact processes important for cancer progression^17,19^. A study by Wang et al. identified a gene set in PDAC regulated at multiple levels by mutation, differential gene expression, and alternative splicing ^20^. While the importance of alternative splicing in controlling cell fate is well established in development, the role and regulation of alternative splicing in cancer and the biological functions of protein isoforms generated from alternately spliced transcripts are not well understood.

Previously, we performed a forward genetic screen in mice using *Sleeping Beauty* insertional mutagenesis to identify genes and processes important for promoting pancreatic cancer progression^21,22^. We identified an enrichment of genes encoding RNA binding proteins (RBPs), including *Mbnl1*, *Mbnl2,* and *Rbfox2*. MBNL1, MBNL2, and RBFOX2 have known roles in promoting alternative splicing of exons in transcripts associated with ESC pluripotency and differentiation. RBFOX2 coordinately regulates alternative splicing with MBNL1 in the differentiation of iPSCs ^14^. Further, RBFOX2 was identified as a key splicing regulator in ovarian and breast cancers responsible for the overlapping exon splicing patterns in these two cancers ^8^. Both cancer types exhibit RBFOX2 downregulation, either by transcriptional control in ovarian cancer or by alternative splicing in breast cancer, resulting in a reduction of the nuclear RBFOX2 isoform. The role of RBFOX2 in directing alternative splicing in pancreatic cancer is not known.

In this study, we uncovered a role for RBFOX2 as a tumor suppressor of pancreatic cancer metastasis in mouse models. Utilizing ex vivo and in vivo approaches, we defined a network of RBFOX2-controlled alternative splicing events linked to cytoskeletal remodeling and specifically associated exon-skipping in *ABI1* with enhanced migratory potential and enrichment at the cell periphery of pancreatic cancer cells. These RBFOX2-mediated exon splicing events occur in pancreatic cancer patients, with differential splicing for a subset of splicing events observed across PDAC sub-types. Our studies link RBFOX2-associated alternative splicing of *ABI1* to pancreatic cancer progression.

## Results

### *RBFOX2* is a progression driver in pancreatic cancer

**Fig. 1:**
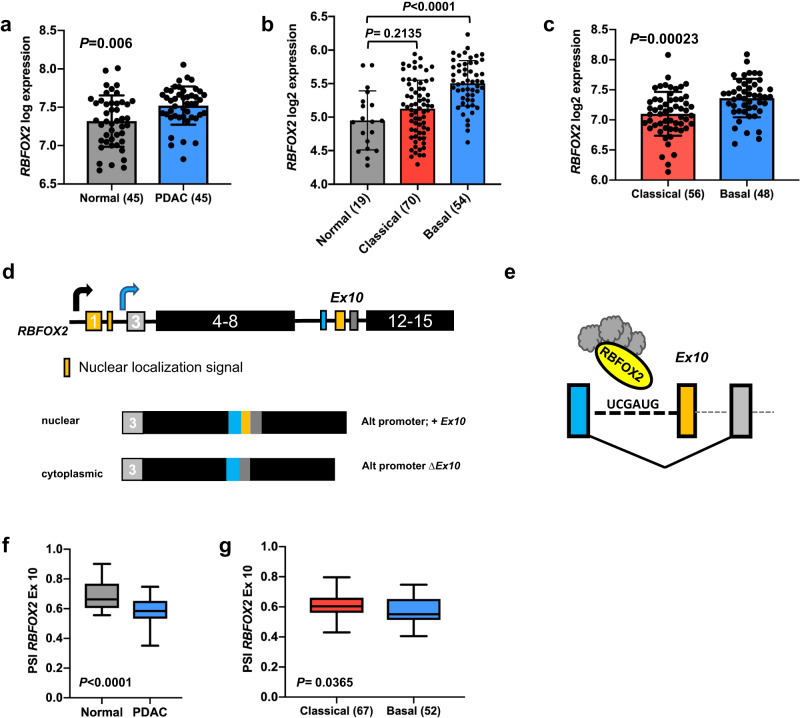
RBFOX2 is differentially expressed and alternatively spliced in pancreatic cancer. *RBFOX2* expression analysis using microarray data from GEO dataset GSE28735 ^27^ showed a significant increase in *RBFOX2* expression in tumors (*n* = 45) compared to normal pancreas (*n* = 45) (**a** FDR adj. *P* = 0.006, two-sided t-test). Gene expression analysis of CPTAC RNA-seq data^34^ generated from normal pancreas and resected pancreatic cancer specimens showed *RBFOX2* gene expression is significantly higher in tumors of the basal subtype (*n* = 54) compared to normal pancreas (*n* = 19) (**b** adj. *P* < 0.0001, ordinary one-way ANOVA with Tukey’s multiple comparisons, DF = 138) and no difference between normal pancreas and the classical subtype (*n* = 70) (adj. *P* = 0.2135, two-sided t-test). *RBFOX2* expression is significantly increased in basal (*n* = 48) compared to classical (*n* = 56) PDAC in RNA-seq data from E-MTAB-6830^35^ (**c** Wilcoxon Rank-sum test, *P* = 0.00023). The canonical full length *RBFOX2* transcript includes two N-terminal two exons encode an N-terminal nuclear localization signal, while downstream exon 10 (ENSG00000100320:015) encodes an internal NLS (**d**). *RBFOX2* transcripts in the pancreas utilize an internal start codon present in exon 3 and lack the N-terminal exons. *RBFOX2* transcripts show alternative splicing of exon 10 in mature transcripts. The RBFOX2 recognition sequence UCGAUG is present upstream of exon 10 (**e**). RBFOX2 binding at this site is predicted to facilitate skipping of exon 10 in mature transcripts. Analysis of percent spliced-in (PSI), a measure of exon inclusion, for alternatively spliced *RBFOX2* exon 10 in *RBFOX2*transcripts shows a significant increase in the inclusion of exon 10 in normal pancreas (*n* = 20) compared to PDAC (*n* = 136), (**f** CPTAC, *P* < 0.0001, 2-tailed t-test, t = 4.700, df=159). *RBFOX2* exon 10 PSI was significantly different between classical and basal PDAC tumors (**g** CPTAC^34^, *P* = 0.0365, 2-tailed t-test, t = 2.115, df=117). Samples ±2 SD from the population mean were removed prior to statistical analysis. Data are presented as mean values +/- SD. The box plots define the 25th and 75th percentiles (box), median (solid line) and the minima and maxima (whiskers). Statistical analysis was performed using PRISM and the *P*-values reported for the indicated statistical tests are shown. Source data are provided as a Source data file.

We identified an enrichment of genes encoding RNA binding proteins (GO: 0003723, *P* = 1.2E-16, Enrichr)^23,24^ from in vivo  forward genetic screens using *Sleeping Beauty* (SB) insertional mutagenesis in a GEMM model of pancreatic cancer^21,22^. A subset of these genes encodes proteins involved in the regulation of mRNA splicing (GO: 0048024, *P* = 1.09E-09, Enrichr). An oncoprint (Supplemental Fig. 1a) shows the frequency and coincidence of SB insertions in the coding region of eleven splicing regulators statistically defined from a population of 172 pancreas tumors. *Mbnl1* and *Mbnl2*, the most frequent “hits” present in 25% and 31% of tumors, respectively, are RNA-binding proteins previously described for their roles in splicing regulation in human ES cells and cancer^11–14^. *Rbfox2*, the third most frequently hit gene (24% of tumors), encodes the RNA splicing factor RBFOX2, the most ubiquitously expressed protein of the FOX family members, which also includes RBFOX1 and RBFOX3 ^25^. RBFOX2 is essential for human ES cell survival ^26^ and is associated with developmental fates ^14^ and cancer progression ^8^. The pattern of SB insertions in the *Rbfox2* locus suggested that gene expression was selectively disrupted in the murine PDAC tumors (Supplemental Fig. 1b). However, analysis of *RBFOX2* gene expression in normal human pancreas and pancreatic tumors from GEO dataset GSE28735 ^27^ revealed a significant increase in *RBFOX2* expression in tumors compared to normal (Fig. 1a, FDR adj. *P* = 0.006). In contrast, *MBNL2* was significantly decreased in tumors compared to normal pancreas (Supplemental Fig. 1c (FDR adj. *P* = 0.0321), while *MBNL1* expression was not significantly different (Supplemental Fig. 1d). *RBFOX2* expression has been linked to TGFß-driven epithelial-to-mesenchymal transition (EMT) in breast cancer^28–30^. Pancreatic cancers are broadly sub-classified as Classical (epithelial-like) or Basal (mesenchymal-like) using gene expression signatures defined by Moffitt and colleagues^31–33^. We further investigated the relationship between *RBFOX2* expression in normal pancreas and PDAC subtypes in CPTAC RNA-seq data ^34^ using subtyping information from published metadata. We found *RBFOX2* gene expression is significantly higher in tumors of the basal subtype compared to normal pancreas (b, adj. *P* < 0.0001, one-way ANOVA with Tukey’s HSD test for multiple comparisons), while there was no significant difference in *RBFOX2* expression between normal pancreas and the classical subtype (adj. *P* = 0.2135). Using a second RNA-seq dataset E-MTAB-6830 ^35^, we defined 56 classical tumors and 48 basal tumors using subtype gene expression signatures ^33^ (see Methods) and confirmed a significant increase in *RBFOX2* in the basal subtype compared to the classical subtype (Fig. 1c, Wilcoxon Rank-sum test, *P* = 0.00023). These data confirm the observed increase in *RBFOX2* in the squamous subtype of PDAC reported by Bailey et al. ^31^.

### *RBFOX2* is alternatively spliced in PDAC

*RBFOX2* function is regulated in part by alternative splicing. The first two exons of full-length *RBFOX2* transcripts (NM_001082578.2) encode the 5’ start-site and a canonical nuclear localization signal (Fig. 1d) and this RBFOX2 isoform was characterized in ESCs ^36^. In the pancreas and other differentiated cell types, initiation of *RBFOX2* transcription utilizes an alternative 5’ start-site upstream of exon 3 and an internal nuclear localization signal encoded by a 40-nucleotide exon (ENSG00000100320:015, exon 10 in hg38 and B40 in mouse)^37^. *RBFOX2* transcripts that include exon 10 encode a nuclear isoform, while *RBFOX2* transcripts that lack exon 10 encode a cytoplasmic isoform (Fig. 1d). Using the CPTAC RNA-seq dataset from human pancreatic cancers, we confirmed *RBFOX2* transcripts from the pancreas lack the first two exons present in full length *RBFOX2*. Interrogating the publicly available TCGA SpliceSeq database of annotated splicing events across 33 cancer types from TCGA RNA-seq datasets^38^, we confirmed alternative splicing of *RBFOX2* exon 10 (hg19 Exon 12 in SpliceSeq) in PDAC patients. Alternative splicing is determined by calculating the Percent Spliced In (PSI, ^39^) which defines the percent of total mature mRNA transcripts that include a cassette exon. Further, we interrogated the incidence of a described autoregulatory alternative splicing event in *RBFOX2* exon 6 which removes part of the RNA recognition motif (RRM)^40^ and found this alternative splicing event was not present in PDAC patient samples. RBFOX2 is an RNA binding protein (RBP) that binds a highly conserved 6-nucleotide intronic recognition sequence (UGCAUG), recruits splicing regulators, including HNRNPM ^41^, and facilitates alternative splicing of select exons^16–19^. RBFOX2 binding sites are highly conserved and RBFOX2 splicing regulation of target exons is tightly controlled by both the position and number of recognition sites in intronic sequences adjacent to spliced exons^26,42^. We mapped UGCAUG RBFOX2 binding sites upstream of exon 10 and using publicly available ENCODE Clip-Seq data generated from K562 cells^43^, we found evidence for RBFOX2 binding to its 6-nucleotide recognition sequence positioned upstream of exon 10 (Fig. 1e). These data suggest RBFOX2 itself may be involved in regulating alternative splicing of exon 10 in *RBFOX2* transcripts. Using bulk RNA-seq data, we found the mean PSI for *RBFOX2* exon 10 was significantly lower in PDAC tumors compared to normal pancreas in the CPTAC dataset (Fig. 1f, *P* < 0.0001), with a mean PSI of 0.59 for PDAC and a slight but significant decrease in exon 10 PSI in basal compared to classical PDAC tumors (Fig. 1g, *P* = 0.0365). Further, we showed a significant negative correlation between *RBFOX2* exon 10 PSI and *RBFOX2* expression in the CPTAC PDAC dataset (Supplemental Fig. 2a, Pearson *P* < 0.0001) and in an independent PDAC RNA-seq dataset (EGAD00001004548, Supplemental Fig. 2b, Pearson *P* = 0.0005). RT-PCR analysis showed epithelial-like PDAC cell lines exhibited a lower *RBFOX2* exon 10 PSI compared to mesenchymal-like PDAC cell lines (Supplemental Fig. 2c). Together, these data suggest that *RBFOX2* is upregulated in PDAC tumors with a related increase in exon 10 skipping.

### RBFOX2 protein abundance is decreased in advanced PDAC

**Fig. 2:**
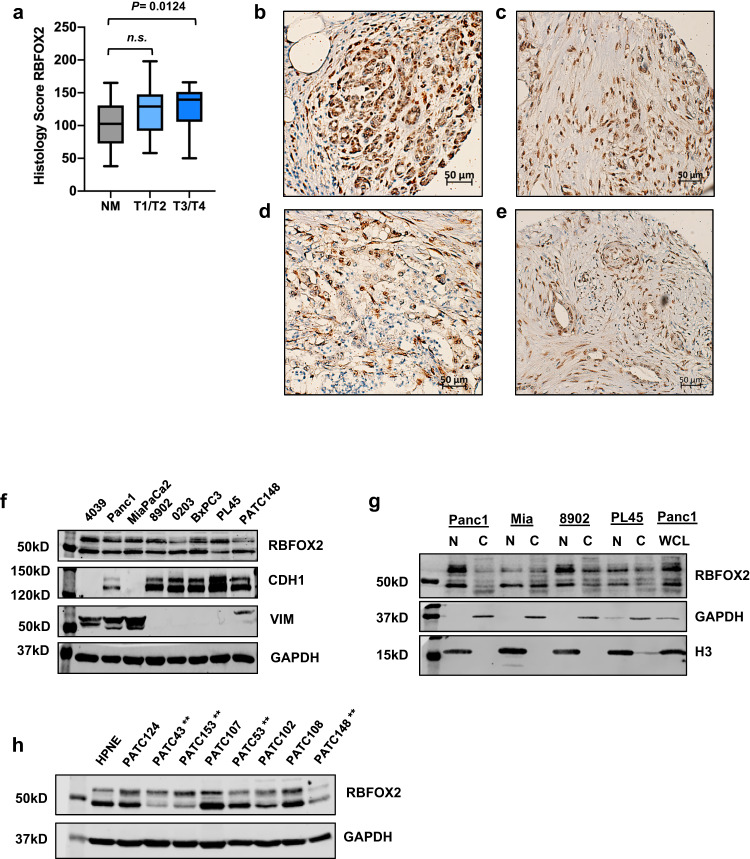
RBFOX2 abundance and localization in pancreatic cancer. RBFOX2 abundance was quantified for non-malignant vs. IDC tumor stage, considering the number of positive nuclei and staining intensity, represented as histology score (**a**). Stage T3/T4 (*n* = 26) PDAC tumors had a significantly higher RBFOX2 histology score compared to NM (*n* = 60) pancreas (*P* = 0.0124, Welch’s ANOVA with Dunnett’s multiple comparisons test, t = 3.010, df=48.15). No significant difference was observed between NM and stage T1/T2 (*n* = 25) (*P* = 0.0542, t = 2.453, df=39.79). Representative images for RBFOX2 IHC are shown for NM (**b**, HS 118), T2 (**c**, HS 180) and T3 (**d**, HS 65 and **e**, HS 165). Scale bar is 50um. HS: Histology Score. In PDAC cell lines, RBFOX2 protein is detected as two distinct bands by western blot at 47kD and 53 kD, with differential intensity of the 53kD band across lines (**f**). Mesenchymal-like cell lines are characterized by expression of vimentin (VIM), and epithelial-like cell lines are characterized by expression of E-cadherin (CDH1). Cell fractionation shows RBFOX2 is predominantly nuclear in Panc1 and PL45 cells, while MiaPaCa2 (Mia) and 8902 cells exhibit both nuclear and cytoplasmic RBFOX2 localization (**g**). Histone H3 marks the nuclear fraction, while GAPDH marks the cytoplasmic fraction. Panc1 whole cell lysate (WCL) is run for comparison of RBFOX2 species and intensities. Western blot analysis of RBFOX2 in human cell lines derived from PDX models of PDAC (**h**) shows cell lines derived from PDXs of individual patients with liver metastasis (marked with **) have lower expression of RBFOX2. RBFOX2 in the immortalized human pancreatic ductal cell line HPNE is shown for comparison. The box plots define the 25th and 75th percentiles (box), median (solid line) and the minima and maxima (whiskers). ns: not significant. Western blots were performed in 3 independent experiments. Source data are provided as a Source data file.

Given the complex regulation of *RBFOX2* at the RNA level in PDAC, we next investigated the abundance and distribution of RBFOX2 protein in resected pancreatic cancers. We stained tissue microarrays constructed at Moffitt Cancer Center for RBFOX2 and scored an average of 1296 tumor nuclei per sample, excluding stroma, from 60 non-malignant (NM) samples and 51 invasive ductal carcinomas (IDC). At the population level, there was no significant difference in the mean percentage of RBFOX2-positive nuclei from non-malignant (NM) or invasive ductal carcinoma (IDC) (Supplemental Fig. 3a, adj. *P* = 0.1744); on average, 60% of scored nuclei were positive (Fig. 2a). Within individual sample cores, the distribution and intensity of RBFOX2 staining was heterogeneous. RBFOX2 was exclusively nuclear in some cells, while other cells within the same core stained for RBFOX2 in the cytoplasm or were devoid of RBFOX2 signal (see representative core images in Supplemental 3). RBFOX2 intensity was high in regions containing acinar cells and early neoplastic lesions. We then quantified the overall nuclear RBFOX2 staining intensity, defined as histology score, for each core and analyzed RBFOX2 across tumor stages included in the TMA analysis (Supplemental Fig. 3b). The non-malignant (NM) samples exhibited the greatest range for RBFOX2 histology scores, which may reflect the variable histology of the NM cores, wherein some cores were comprised of dense regions of acinar cells and little stroma, while other regions exhibited dense stroma with areas of acinar cells and ADM or desmoplasia (see examples in Supplemental Fig. 3c and **e**). We combined the data from stages T1/T2 and T3/T4 for statistical analysis and found no significant difference in the average RBFOX2 histology score (Fig. 2a) between NM and stage T1/T2 (*P* = 0.0542, Welch’s ANOVA with Dunnet’s multiple comparisons test). We did observe a slight but significant increase in the RBFOX2 histology score for stage T3/T4 PDAC tumors compared to NM pancreas (Fig. 2b, adj. *P* = 0.0124). Representative histology images of RBFOX2-stained TMAs are shown for NM, T2 and T3 (Fig. 2b–e). Scanned core images along with the digital image analysis for nuclear RBFOX2 staining intensity are shown in Supplemental Fig. 3. To further characterize RBFOX2 protein abundance in PDAC cell lines, we performed Western blot analysis of RBFOX2 protein abundance in a panel of pancreatic cancer cell lines and found RBFOX2 is present as two major isoforms of 47 kD and 53 kD (Fig. 2f). We observed variable expression of the 53 kD isoform across cell lines. These two species of RBFOX2 are not predicted to represent the alternative splice forms that retain or exclude exon 10, since this exon encodes only 40 amino acids of RBFOX2. Further, we did not observe an appreciable difference in RBFOX2 protein abundance with respect to the epithelial-like or mesenchymal-like properties of the cell lines as defined by the expression of epithelial marker E-cadherin (CDH1) or mesenchymal marker vimentin (VIM). 4039 and MiaPaCa2 cell lines are highly mesenchymal and lack CDH1, while Panc1 and PATC148 cells express both markers. The PATC148 cell line was derived from a PDX model of an invasive PDAC tumor that metastasized to the liver ^44^. Epithelial-like cell lines, including PL45, lack VIM. Quantification of total RBFOX2 protein from whole cell lysate of epithelial-like cell lines 8902, Panc 0203, BxPC3 and PL45 was performed relative to a standard curve of Panc1 whole cell lysate (Supplemental Fig. 4a, b) and demonstrated similar levels of total RBFOX2 expression across these lines, except for PL45, which expresses about half the amount of RBFOX2 present in Panc1 cells (Supplemental Fig. 4c). To further investigate the distribution of RBFOX2 in PDAC cells, we performed cell fractionation and probed for the presence of RBFOX2 in the nuclear and cytoplasmic fractions. We observed almost exclusively nuclear localization of both RBFOX2 species in Panc1 cells, which reflected the data from Panc1 whole cell lysate (WCL, Fig. 2g). Surprisingly, RBFOX2 was present in equal proportion in the nuclear and cytoplasmic fractions for MiaPaCa2 cells. 8902 cells exhibited RBFOX2 in both fractions as well, with more RBFOX2 in the nucleus. In PL45 cells, RBFOX2 was primarily nuclear with about half the intensity of the RBFOX2 signal observed in the nucleus for Panc1 and 8902 cells, consistent with the quantitation data from whole cell lysates (Supplemental Fig. 4c). Together, these data indicate RBFOX2 nuclear intensity and cellular localization is heterogeneous in PDAC. Importantly, we observed decreased RBFOX2 abundance in PDX-derived cell lines from PDAC patients with liver metastases (Fig. 2h, metastatic lines marked with asterisks), suggesting RBFOX2 may have a tumor suppressive role in progression to metastatic PDAC.

#### RBFOX2 depletion promotes cell migration and invasion in pancreatic cancer cells

**Fig. 3:**
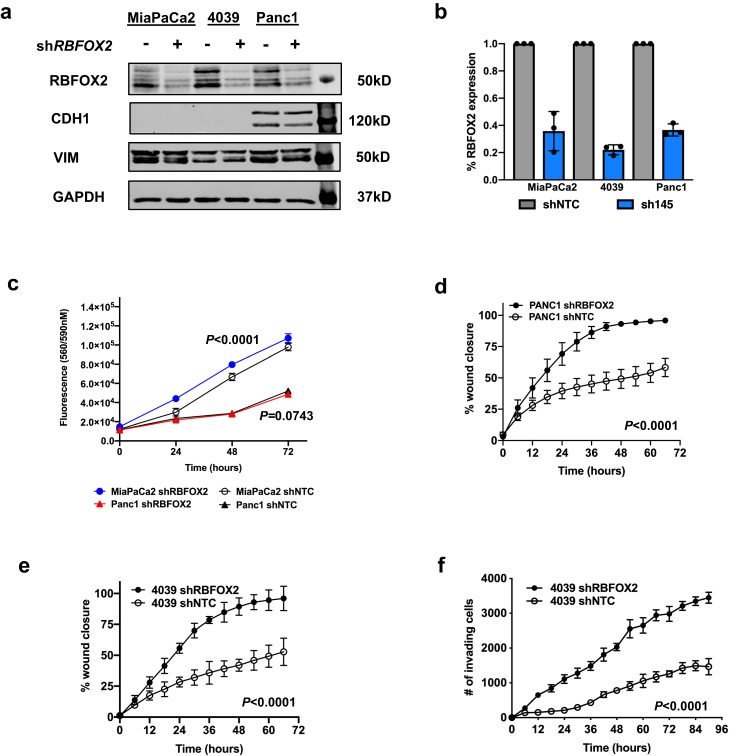
RBFOX2 depletion drives aggressive cellular phenotypes. ShRNA-mediated knock-down of RBFOX2 in mesenchymal-like PDAC cell lines MiaPaCa2, 4039 and Panc1 leads to a significant reduction in RBFOX2 protein (**a**) compared to cells stably transduced with a non-targeting shRNA (shNTC). No change in expression of epithelial marker CDH1 or mesenchymal marker vimentin (VIM) was observed with RBFOX2 knockdown. The percent of remaining RBFOX2 protein upon *RBFOX2* knockdown (sh145) was determined from the average quantification of two cell passages examined in three independent experiments (**b**). MiaPaCa2 cells with RBFOX2 depletion (sh*RBFOX2*) exhibited a slight but significant increase in cellular growth compared to control cells (shNTC) assessed using Cell Titer Blue assays (**c,** *P* < 0.0001, two-way ANOVA; DF = 3; F = 9.6); cell proliferation was unchanged in RBFOX2-depleted Panc1 cells (*P* = 0.0743, two-way ANOVA; DF = 3; F = 2.550). Cell migration is significantly increased upon RBFOX2 depletion in Panc1 cells (**d,** *P* < 0.0001, two-way ANOVA; DF = 23; F = 21.88) and in 4039 cells (**e,** *P* < 0.0001, two-way ANOVA; DF = 11; F = 22.99) using a wound healing assay. Cell invasion is significantly increased upon RBFOX2 depletion in 4039 cells (**f,** *P* < 0.0001, two-way ANOVA; DF = 15; F = 120) using an Incucyte chemotaxis assay. Data in panels c-f are representative of three independent experiments generated across three cell passages, with 6-8 technical replicates plated per line per experiment. Data are presented as the mean values +/- SD of the technical replicates from a single experiment. Statistical analysis was performed using PRISM and the *P*-values calculated for the indicated statistical tests are shown. Source data are provided as a Source data file.

Based on our prediction of *Rbfox2* as a tumor suppressor from our in vivo screen and our observation of decreased RBFOX2 in PDX-derived cell lines from metastatic PDAC, we further investigated the potential role of RBFOX2 downregulation in promoting pancreatic cancer progression. We performed stable depletion of RBFOX2 in MiaPaCa2, 4039 and Panc1 cells using a constitutively expressed shRNA (Fig. 3a) or inducible shRNA (Supplemental Fig. 5a) and showed RBFOX2 protein was significantly depleted while expression of VIM and CDH1 was unchanged. Relative quantification of RBFOX2 protein knockdown is shown (Fig. 3b and Supplemental Fig. 5b). MiaPaCa2 cells with constitutive RBFOX2 depletion exhibited a slight but significant increase in proliferation in 2D cultures compared to cells with a non-targeting control shRNA (Fig. 3c, *P* < 0.0001, two-way ANOVA), while Panc1 cells showed no difference in proliferation. No change in cellular proliferative capacity was observed with inducible RBFOX2 knockdown in MiaPaCa2 cells (*P* = 0.2098) or for Panc1 cells (*P* = 0.9780, Supplemental Fig. 5c). Interestingly, constitutive RBFOX2 depletion significantly increased cell migration in Panc1 and 4039 cells (Fig. 3d, e, two-way ANOVA, *P* < 0.0001) using a wound healing assay and significantly increased cell invasion in 4039 cells (Fig. 3f two-way ANOVA, *P* < 0.0001) using an Incucyte chemotaxis assay. Induced RBFOX2 knockdown in 4039 cells significantly increased cell migration compared to control cells (Supplemental Fig. 4d, *P* < 0.001, two-way ANOVA). Together, these data suggest that RBFOX2 reduction in mesenchymal-like PDAC cell lines promotes cell mobility.

### Inclusion of exon 10 promotes nuclear RBFOX2 repletion

**Fig. 4:**
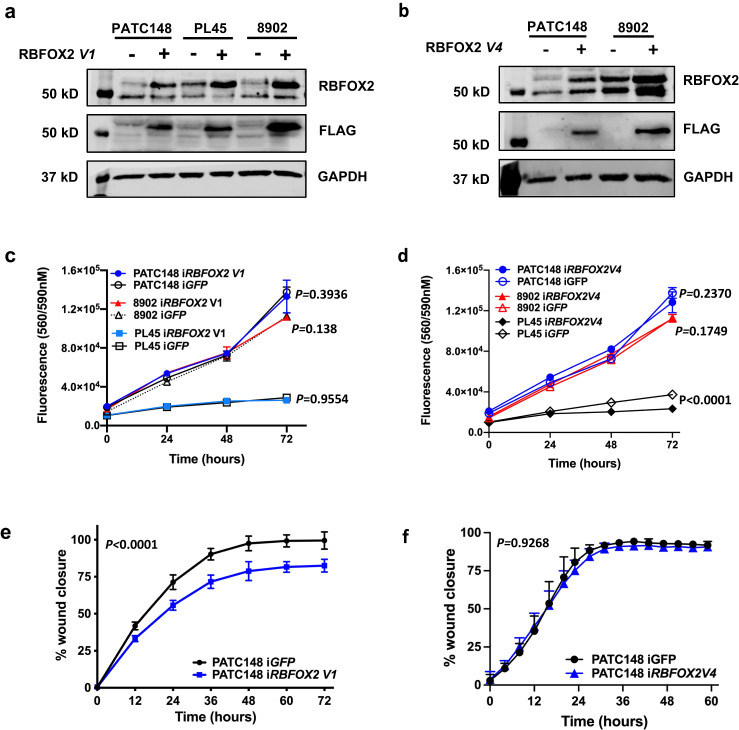
Nuclear RBFOX2 repletion rescues cell migration in PDAC cells. Inducible Flag-tagged nuclear *RBFOX2* (*V1*) or cytoplasmic *RBFOX2* (*V4*) cDNA isoforms reconstitute RBFOX2 protein levels in PATC148, PL45 and 8902 cells (**a**, **b**). Detection of the Flag-tag confirms expression of the exogenous cDNA. No change in proliferation was detected for cells expressing i*RBFOX2 V1* compared to control cells (*iGFP*) grown on 2D (**c**). PATC148, *P* = 0.3936, DF = 3, F = 1.029; 8902 *P* = 0.138, DF = 3, F = 4.182; PL45 *P* = 0.9554, DF = 3, F = 6.716, two-way ANOVA). Induction of i*RBFOX2* *V4* did not change the proliferative capacity of 8902 or PATC148 cells but slightly decreased proliferation in PL45 cells (**d**). PATC148, *P* = 0.2370, DF = 3, F = 12.13; 8902 *P* = 0.1749, DF = 3, F = 1.766; PL45 *P* < 0.0001, DF = 3, F = 114.2, two-way ANOVA. Cell migration in PATC148 i*RBFOX2 V1* cells was significantly decreased compared to PATC148 i*GFP* control cells using a wound-healing assay (**e**, *P* < 0.0001, DF = 6, F = 9.003, two-way ANOVA). Expression of RBFOX2 *V4* does not change the migratory capacity of PATC148 cells in a wound healing assay (**f**, P = 0.9268, DF = 18, F = 0.5557, two-way ANOVA). Data in panels c-f are from individual representative experiments, with 6 technical replicates plated per line per experiment. Three independent experiments were performed across three cell passages. Data are presented as mean values +/- SD. Statistical analysis was performed using PRISM and the *P*-values reported for the indicated statistical tests are shown. Source data are provided as a Source data file.

We next investigated whether RBFOX2 nuclear repletion in epithelial-like cells would affect cell survival and migration in vitro. We stably transduced PATC148, PL45, and 8902 cells lines with doxycycline-inducible vectors expressing Flag-tagged cDNAs for nuclear RBFOX2 (i*RBFOX2* V1, Fig. 4a), cytoplasmic RBFOX2 (i*RBFOX2* V4, Fig. 4b) or a GFP control cDNA (i*GFP*). The FLAG tag confirmed the induction of RBFOX2 cDNAs. We achieved similar expression for each induced RBFOX2 isoform in each cell line panel. Quantification of total RBFOX2 in the V1 or V4 expressing lines compared to the GFP control is shown for PATC148 and 8902 cells (Supplemental Fig. 6a). Cellular fractionation followed by western blot analysis confirmed the nuclear localization of the V1 isoform and unexpected but reproducible detection of RBFOX2 V1 in the cytoplasm (Supplemental Fig. 6b, c). These data confirm that exon 10 is required for RBFOX2 nuclear import. The presence of V1 in the cytoplasm may reflect additional non-splicing related regulation of RBFOX2 nuclear import in PATC148 cells or may reflect steady-state RBFOX2 processing. In contrast, RBFOX2 V4 lacking exon 10 remained exclusively in the cytoplasm of PATC148 cells, as expected. We observed no difference in the proliferative capacity of cells expressing induced *RBFOX2* V1 compared to GFP (Fig. 4c, see indicated *P*-values for each cell line pair, two-way ANOVA), or for PATC148 or 8902 cells expressing induced *RBFOX2* V4 (Fig. 4d, see indicated *P*-values for each cell line pair, two-way ANOVA). PL45 cells exhibited a slight but significant decrease in proliferation with induced V4 (Fig. 4d, *P* < 0.0001, two-way ANOVA). Induced *RBFOX2* V1 in PATC148 cells significantly reduced cell migration compared to iGFP control cells measured by a wound-healing assay (Fig. 4e, *P* = 0.0005, two-way ANOVA), while induced *RBFOX2* V4 had no effect on PATC148 cell migration (Fig. 4f, *P* = 0.9258, two-way ANOVA). 8902 cells closed the wound too quickly to be able to distinguish a difference between RBFOX2 isoforms, while PL45 cells poorly migrate in a wound healing assay.

### RBFOX2 depletion drives aggressive PDAC in vivo

**Fig. 5:**
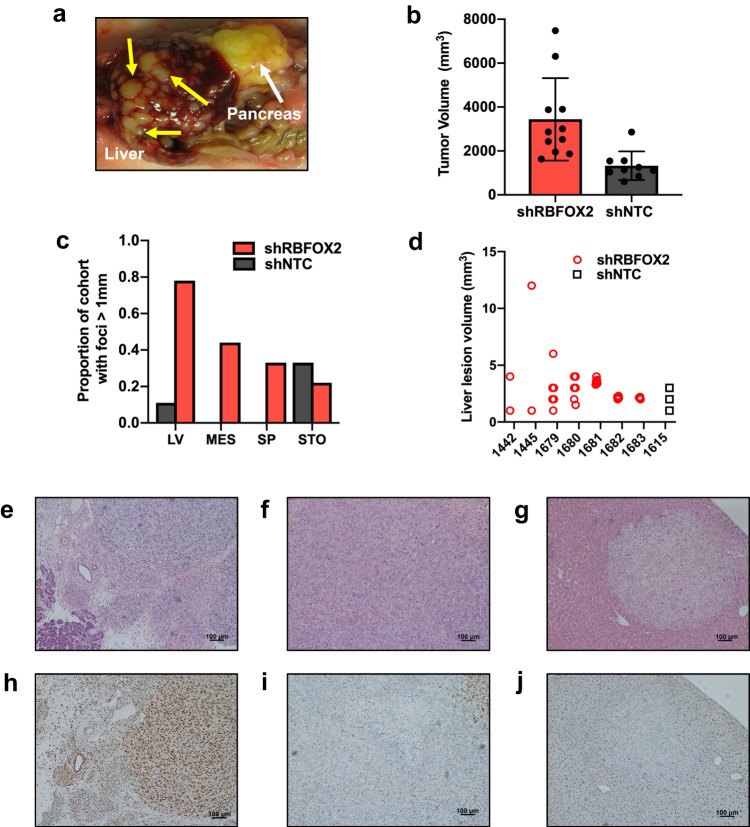
RBFOX2 depletion drives aggressive pancreatic cancer in vivo. Orthotopic injection of 4039 cells depleted for RBFOX2 promotes an aggressive disease in NSG mice (**a**). The pancreas tumor is labeled by the white arrow; multiple lesions in the liver are denoted by yellow arrows. Pancreas tumors derived from 4039 cells lacking RBFOX2 (*n* = 11, red bars) have an increased tumor volume compared to tumors derived from control cells (*n* = 9, black bars) (**b**). Data are combined from two independent experiments with randomly assigned male and female mice. Mice with RBFOX2-depleted cells exhibit a higher incidence of metastatic lesions to the liver (LV), mesentery (MES) and spleen (SP), quantified as the percent of mice with at least one metastatic focus measured > 1 mm at necropsy (**c**). The volume of liver lesions (mm^3^) collected at necropsy is graphed on a per-animal basis (**d**). Only one mouse from the control cohort exhibited liver lesions. Histological analysis by H&E staining of pancreas tumors from 4039 cells expressing the non-targeting shRNA (**e**) shows tumor with surrounding acinar cells. Pancreas tumors with RBFOX2 depletion (**f**) have little surrounding normal pancreas. H&E staining from an RBFOX2-depleted liver lesion (**g**). Analysis of RBFOX2 expression by immunocytochemistry demonstrates robust RBFOX2 expression in tumors from replete cells (**h**) and an absence of signal in RBFOX2 depleted pancreas tumor (**i**) and resulting liver metastasis (**j**). RBFOX2 expression is decreased in the normal liver compared to normal pancreas. STO= stomach. Scale bar is 100um. Data are presented as mean values +/- SD. Source data are provided as a Source data file.

Given that RBFOX2 nuclear depletion promotes aggressive in vitro phenotypes in PDAC cells, we next investigated whether RBFOX2 depletion impacted PDAC tumor development in vivo. 4039 or Panc1 cells stably transduced with a constitutive shRNA targeting *RBFOX2* or the non-targeting shRNA were introduced directly into the pancreas of NSG male and female mice by surgical injection. After 45 days, mice injected with 4039 cells with RBFOX2 depletion exhibited highly aggressive pancreatic disease, with large primary tumors and multiple lesions to the liver (Fig. 5a). Mice injected with Panc1 cells depleted for RBFOX2 showed similar phenotypes. Tumor volumes were increased in RBFOX2-depleted tumors compared to RBFOX2-replete tumors with either 4039 cells (Fig. 5b) or Panc1 cells (Supplemental Fig. 7a). RBFOX2-depleted cells exhibited an enhanced ability to migrate and seed several organs based on the presence of foci expressing the GFP reporter. 80% of the mice with RBFOX2-depleted tumors developed foci in the liver greater than 1 mm, compared to only 10% of control mice, (Fig. 5c). Liver lesions from RBFOX2-depleted tumors exhibited both an increased size and multiplicity compared to RBFOX2-replete tumors (Fig. 5d). Notably, several mice with RBFOX2-depleted cells exhibited more liver lesions than could be measured. In addition, these mice exhibited foci in the mesentery, spleen and stomach. Mice injected with RBFOX2-depleted Panc1 cells also exhibited an increased incidence of liver foci (Supplemental Fig. 7b) and an increase in both the size and multiplicity of metastatic lesions in the liver (Supplemental Fig. 7c) compared to control. Histological analysis of pancreas tumors from RBFOX2 replete tumors by H&E staining showed tumor development with some normal pancreas within and surrounding the tumor (Fig. 5e and Supplemental Fig. 7d), while tumors derived from RBFOX2 depleted cells showed large areas of tumor growth with little stromal infiltrate and some normal pancreas surrounding the tumor area (Fig. 5f and Supplemental Fig. 7e). Colonization of RBFOX2-depleted cells in the liver (Fig. 5g and Supplemental Fig. 7f) is surrounded by normal tissue. Immunohistochemistry confirmed RBFOX2 expression in the tumor and surrounding cells (Fig. 5h and Supplemental Fig. 7g) and the absence of RBFOX2 expression in the RBFOX2-depleted pancreas tumor (Fig. 5i and Supplemental Fig. 7h) and related liver lesions (Fig. 5j and Supplemental Fig. 7i. Further, we showed that RBFOX2 depletion in a murine cell line derived from the KPC PDAC GEMM (Supplemental Fig. 8a) promotes PDAC tumor growth in a C57BL/6 J immune competent orthotopic model (Supplemental Fig. 8b). Four out of 5 mice with RBFOX2 depleted cells developed lung lesions using a tail vein metastatic colonization model in C57BL/6 J mice, while only one of five mice in the control group developed lung lesions (Supplemental Fig. 8c). Together, these in vivo data support a tumor suppressive role for RBFOX2 in metastatic progression.

### Reduced RBFOX2 nuclear abundance promotes exon skipping in cytoskeletal remodeling transcripts in pancreatic cancer

**Fig. 6:**
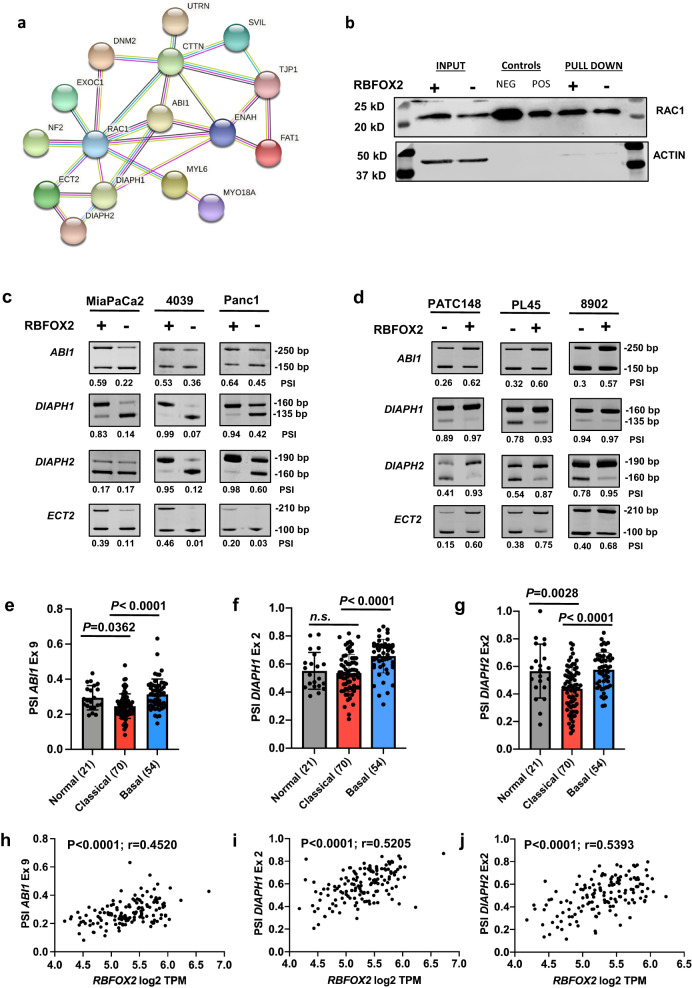
RBFOX2 depletion promotes exon skipping in actin regulators. RBFOX2 splicing targets are significantly enriched for protein interactions through RAC1 (**a**, STRING PPI *P* < 7.48E-14). Levels of GTP-bound RAC1 in Panc1 cells replete and depleted for RBFOX2 were unchanged using a GTP pulldown assay (**b**). Data are representative of three independent experiments generated across three cell passages. RT-PCR analysis of RBFOX2 target exons in *ABI1*, *DIAPH1*, *DIAPH2* and *ECT2* shows a shift towards exon exclusion in the absence of RBFOX2 in MiaPaCa2, 4039 and Panc1 isogenic cell line pairs (**c**). PSI values (Percent spliced-in) calculated for each isogenic pair quantify the decrease in transcripts encoding the long isoform (LF) upon RBFOX2 depletion. Reconstitution of RBFOX2 in PATC148, 8902 and PL45 cells promotes exon inclusion of RBFOX2 target exons in *ABI1*, *DIAPH2* and *ECT2* (**d**). PSI values quantify the increase in transcripts encoding the long mRNA isoform (LF). Splicing assays are representative of three independent RT-PCR assays with PSI quantitation across multiple cell passages. Comparison of RBFOX2 target exon PSI values in normal pancreas and PDAC subtypes using CPTAC RNA-seq data ^34^ revealed differences in the population mean and variance. The mean PSI for *ABI1* exon 9 in classical PDAC is significantly lower than for normal pancreas (**e**, *P* = 0.0362) and basal PDAC (*P* < 0.0001). The mean PSI for *DIAPH1* exon 2 in classical PDAC is significantly lower than for basal PDAC (**f,** *P* < 0.0001) but not significantly different from normal pancreas. The mean PSI for *DIAPH2* exon 2 in classical PDAC is significantly lower than for normal pancreas (**g,** *P* = 0.0028) and basal PDAC (*P* < 0.0001). *P*-values for each panel were calculated using one-factor ANOVA with Tukey’s multiple comparisons test. Data in bar graphs are presented as mean values +/- SD. RBFOX2 target exon abundance (calculated as PSI) positively correlates with *RBFOX2* expression (log2 TPM) in the CPTAC RNA-seq data set ^34^ for *ABI1* exon 9 (*n* = 140 XY pairs) (**h**, Pearson’s correlation, *P* < 0.0001; r = 0.4520, 95% confidence interval 0.3093 to 0.5748), *DIAPH1* exon 2 (*n* = 140 XY pairs) (**i**, Pearson’s correlation, *P* < 0.0001; r = 0.5205, 95% confidence interval 0.3881 to 0.6318) and *DIAPH2* exon 2 (*n* = 139 XY pairs) (**j**, Pearson’s correlation, *P* < 0.0001; r = 0.5393, 95% confidence interval 0.4096 to 0.6476). *P*-values are reported exactly as computed by PRISM. Source data are provided as a Source data file.

RBFOX2 has been described as an important regulator of EMT splicing signatures in breast cancer and cancer cell line models^30,45^. To define splicing events regulated by RBFOX2 in PDAC, we utilized Applied Biosystems Clariom D arrays (see Methods) to profile exon level expression in the cell line pairs replete and depleted for RBFOX2 (MiaPaCa2, 4039, and Panc1) and in the orthotopic primary tumors (4039 and Panc1). We statistically identified 27 differentially spliced exons from a combined analysis of high-vs-low RBFOX2 cell lines and 42 differentially spliced exons from pancreas tumors replete and depleted for RBFOX2 (Supplementary Data 1). Eighteen of these alternatively spliced exons overlapped between the in vitro and in vivo analyses. In addition, we identified 56 intron retention, 14 alternative 5’ donor site and 4 alternative 3’ acceptor site events in vitro and 61 intron retention, 20 alternative 5’ donor site and 13 alternative 3’ acceptor site events. No intron retention or alternative 5’ donor site events were conserved between the in vitro and in vivo analyses, while only a single alternative 3’ acceptor site event was shared. In addition, we identified 240 differentially expressed protein coding genes with a 2-fold or greater change in expression between RBFOX2 replete and depleted pancreas tumor samples (Supplementary Data 1). Given the evidence for conserved exon splicing events in cancer and our limited understanding of these events in PDAC, we further characterized the differential exon splicing events associated with RBFOX2 in our pancreas cell line and tumor models. RBFOX2 binding to its recognition site(s) downstream of an alternatively spliced exon facilitates the inclusion of the target exon in processed transcripts, while RBFOX2 binding to its recognition site(s) present upstream of an alternatively spliced exon leads to exon skipping^36,41,46^. By overlaying mapped RBFOX2 binding sites^26,42^ with these spliced exons, we confirmed all 27 exons from the in vitro analysis and 35 of the exons from the in vivo analyses are flanked by conserved intronic RBFOX2 recognition sites present within 500 bp of the spliced exon, suggesting these exons are direct RBFOX2 targets. Further, the placement of the UCGAUG sequence predicted the direction of the exon splicing change upon RBFOX2 depletion. This subset of non-overlapping 44 alternatively spliced exons occurs in transcripts encoding structural or signaling proteins enriched in cytoskeletal function (GO:0005856, *P* = 8.68E-07, Enrichr). The encoded proteins interact with a frequency greater than expected by chance, with multiple connections through Rac1 (Fig. 6a, STRING PPI *P* < 7.48E-14)^47,48^. Further, 11 exons map to genes statistically defined as candidate cancer genes in our SB-PDAC model^21,22^, including *Abi1* and *Dia1* (see Supplemental Fig. 9 for oncoprint of insertions). Notably, we found no difference in levels of GTP-bound Rac1 in Panc1 RBFOX2 replete and depleted cells (Fig. 6b) or in 4039 RBFOX2 replete and depleted cells (Supplemental Fig. 10a) using GTP-pulldown assays. We validated the relationship between RBFOX2 abundance and exon splicing events in PDAC cell lines by RT-PCR using primers flanking the target exon. *ABI1*, *DIAPH1, DIAPH2* and *ECT2* showed consistent exon skipping across cell lines with constitutive (Fig. 6c) or induced RBFOX2 depletion (Supplemental Fig. 10b). In MiaPaCa2 cells, skipping of the RBFOX2-target exon in *DIAPH2* occurs in the presence of RBFOX2, suggesting *DIAPH2* exon splicing in this cell line is regulated by another RBP(s). Induced nuclear RBFOX2 repletion in PATC148, 8902, and PL45 cells reverted exon skipping and increased the PSI for *ABI1*, *DIAPH2,* and *ECT2*, confirming these exons are alternatively spliced in response to nuclear levels of RBFOX2 in PDAC cells (Fig. 6d). As expected, repletion of the cytoplasmic RBFOX2 V4 isoform did not revert RBFOX2-mediated splicing of these targets (Supplemental Fig. 10c). The PSI for *DIAPH1* was high in all control cell lines and splicing was unaffected by RBFOX2 repletion. *ABI1* splicing has not been previously associated with EMT or described in PDAC^30,45^. Interestingly, *DIAPH2* PSI in 8902 control cells was like that observed in the mesenchymal-like RBFOX2 replete cell lines. To investigate whether a further reduction of RBFOX2 in 8902 cells would alter the splicing of *DIAPH2* and other targets, we generated 8902 cell line pairs with RBFOX2 knockdown using either the constitutive or inducible *RBFOX2* shRNAs. RBFOX2 protein knockdown was determined by western blot (Supplemental Fig. 10d). RBFOX2 depletion in 8902 cells further reduced the PSI for *ABI1* and *ECT2* (Supplemental Fig. 10e) and facilitated exon exclusion in *DIAPH1* and *DIAPH2*. These data suggest that the splicing regulation of RBFOX2 target exons is regulated by RBFOX2 nuclear abundance, which can be similar in mesenchymal-like and epithelial-like cell lines. Additional differentially spliced RBFOX2 target exons with a 20% or greater change in PSI upon RBFOX2 depletion are shown for the cell line panel in Supplemental Fig. 10f).

We next investigated whether these RBFOX2 target exons are alternatively spliced in pancreatic cancer patients. Using the CPTAC dataset^34^, we defined the PSIs for RBFOX2 target exons in normal pancreas and in PDAC subtypes. Tumors of the classical PDAC subtype had a significantly lower PSI for *ABI1* exon 9 compared to both normal pancreas (*P* = 0.0362) and basal PDAC (Fig. 6e, one-way ANOVA *P* < 0.0001), consistent with our cell line data. For *DIAPH1* exon 2, the PSI was highly variable in classical tumors, with a mean of 0.5, which was significantly lower than the mean PSI in basal tumors (Fig. 6f, one-way ANOVA, *P* < 0.0001). For *DIAPH2* exon 2, the PSI was significantly lower in classical tumors compared to normal pancreas (*P* = 0.0028) or to basal tumors (Fig. 6f, one-way ANOVA, *P* < 0.0001). We found similar relationships between RBFOX2 target exon PSI values and PDAC subtypes using the EGAD00001004548 RNA-seq dataset^1,49–51^ (Supplemental Fig. 11a–c). Next, we investigated the relationship between *RBFOX2* gene expression (log2 Transcripts Per Million, TPM) and the PSI of the defined RBFOX2 target exons. We found a positive correlation for both the CPTAC (Pearson correlation *P* = 0.0009) and EGAD00001004548 RNA-seq datasets (Pearson correlation *P* = 0.0009) using a permutation test to compare the average absolute correlation between the PSI of spliced exons and *RBFOX2* expression with the average absolute correlation of randomly selected exons and *RBFOX2* expression in resected human PDAC patients (see Methods and Supplementary Data 2). Scatterplots for RBFOX2 target exons with *RBFOX2* expression (log2 TPM) from the CPTAC data are shown for *ABI1, DIAPH1* and *DIAPH2* (Fig. 6h–j). Scatterplots for *RBFOX2* gene expression with *RBFOX2* target exon PSI for *DIAPH1*, *ABI1* and *DIAPH2* in the EGAD00001004548 RNA-seq is shown for PDAC (Supplemental Fig. 11d–f) and for liver metastases (Supplemental Fig. 11g–i, and Supplementary Data 2). Taken together, these data demonstrate RBFOX2 primarily mediates alternative splicing of a subset of exons in pancreatic cancer involved in cytoskeletal remodeling through RAC1 downstream targets, and this splicing is driven by RBFOX2 nuclear abundance, with variable RBFOX2 expression and PSI values for target exons observed across cell lines and patient samples.

### RBFOX2 depletion promotes ABI1 isoform switching through *ABI1* alternative splicing

**Fig. 7:**
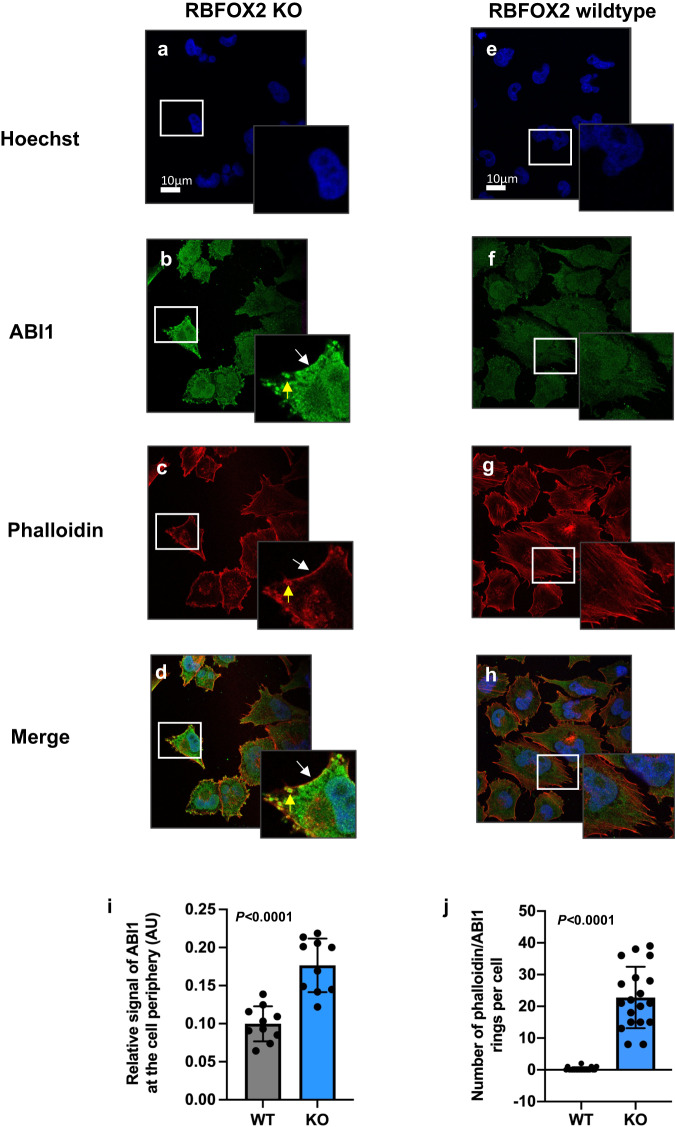
RBFOX2 loss promotes ABI1 cellular redistribution in PDAC cells. MiaPaCa2 cells with CRISPR-mediated RBFOX2 depletion (**panels a**–**d**) exhibit a redistribution of ABI1 (**b**) to the periphery of cells (see inset, white arrow) and coincident with F-actin (**c**, see inset, white arrow). The formation of actin into ring-like structures at the cell periphery was distinctive in RBFOX2 deficient cells (**c;** see inset, yellow arrow indicates actin ring). The merged image (**d**) shows overlap between ABI1 and F-actin at the cell periphery (inset, white arrow) and in actin rings (inset, yellow arrow). In contrast, RBFOX2-replete cells (**panels e**–**h**) exhibit a uniform distribution of ABI1 throughout the cell (**f**) and filamentous F-actin (**g**). The merged image (**h** and inset) shows no overlap of ABI1 and F-actin at the cell periphery and the absence of actin ring-like structures. Nuclei were stained using Hoechst 33342 (**a**, **b**). **g** Quantification of the relative ABI1 signal at the cell periphery (**I**, arbitrary units (AU)) shows significantly increased ABI1 in RBFOX2 depleted (KO) cells (*n* = 10) compared to RBFOX2 replete (WT) cells (*n* = 10; *P* < 0.0001, t-test; t = 5.770, df=18). The number of phalloidin/ABI1 rings is significantly increased in RBFOX2 depleted (KO) cells (*n* = 19) compared to RBFOX2 replete (WT) cells (*n* = 20; **j,** *P* < 0.0001, t-test; t = 10.42, df=37). Data are presented as mean values +/- SD. Scale bar is 10um. *P*-values are reported exactly as computed by PRISM. Source data are provided as a Source data file.

RBFOX2 target exons are enriched in transcripts involved in cytoskeletal remodeling and cell adhesion, including *ABI1*. Given that *Abi1* was also identified as a hit in our in vivo forward genetic screen for PDAC (Supplemental Fig. 9), we wanted to investigate how alternative splicing of ABI1 may impact ABI1 function. ABI1 is part of the WAVE regulatory complex (WRC) that regulates actin remodeling through interactions with the Arp2/3 complex to promote cell migration^52,53^. Western blot analysis of ABI1 protein isoforms identified a shift toward increased abundance of ABI1∆Ex9 in response to RBFOX2 knockdown, consistent with the splice shift we observed in *ABI1* transcripts (Supplemental Fig. 12a). Panc1 cells exhibit three ABI1 isoforms, with a distinct decrease of ABI1 full length (FL) in the absence of RBFOX2 and increased abundance of a smaller isoform. RBFOX2 repletion in PATC148 cells resulted in an enrichment of ABI1 FL protein, consistent with increased exon inclusion (Supplemental Fig. 12b). These data demonstrate the abundance of ABI1 protein isoforms can be predicted by the PSI of exon 9 in *ABI1* RNA transcripts. To investigate whether RBFOX2 depletion influenced ABI1 cellular localization, we generated Panc1 and MiaPaCa2 cells with CRISPR-mediated *RBFOX2* knockout (Supplemental Fig. 12c) and confirmed isoform switching of *ABI1* at both the RNA (Supplemental Fig. 12d, e) and protein level (Supplemental Fig. 12f). Using confocal imaging, we then investigated ABI1 localization in RBFOX2 depleted and replete MiaPaCa2 cells. Nuclei were stained with Hoeschst (Fig. 7a, e). RBFOX2-depleted MiaPaCa2 cells exhibited a redistribution of ABI1 to the cell periphery (Fig. 7b, white arrow), coincident with F-actin staining (Fig. 7c, white arrow and merged image in panel d). Unexpectedly, we also observed the presence of actin rings in the cell periphery that showed dense ABI1 colocalization (Fig. 7b, c, yellow arrow). In comparison, MiaPaCa2 control cells replete for RBFOX2 (WT) exhibited a more uniform distribution of ABI1 throughout the cytoplasm (Fig. 7f) and actin stress fibers marked by F-actin (Fig. 7g), with little overlapping signal (merged image, Fig. 7h) and the absence of actin rings. Quantification of relative ABI1 signal at the cell periphery was significantly greater in RBFOX2 knockout (KO) cells compared to control cells (WT, Fig. 7i, *P* < 0.0001, t-test). The quantification of phalloidin/ABI1 rings per cell is shown in Fig. 7j (*P* < 0.0001, t-test). To further define the redistribution of ABI1 to the cell periphery in the absence of RBFOX2, we co-stained RBFOX2 KO cells with ABI1 and Cortactin (Supplemental Fig. 13a–d). Cortactin is a well-established marker of lamellipodia in motile cells and is localized with the Arp2/3 complex at sites of actin polymerization within lamellipodia^54^. Cortactin binds and activates the Arp2/3 complex and regulates actin polymerization^55^. ABI1 staining (Supplemental Fig. 13b) was coincident with Cortactin (Supplemental Fig. 13c) at the cell periphery of RBFOX2 KO cells, with robust overlapping signal in cellular protrusions (Supplemental Fig. 13d). In contrast, ABI1 and Cortactin were uniformly distributed throughout RBFOX2 replete cells, with no enrichment at the cell membrane (Supplemental Fig. 13e–h). Overall, these data suggest that the ABI1∆Ex9 isoform is redistributed to the cell periphery in the absence of RBFOX2 and is enriched in structures marked by Cortactin.

### ABI1 splice-switching promotes cell migration

**Fig. 8:**
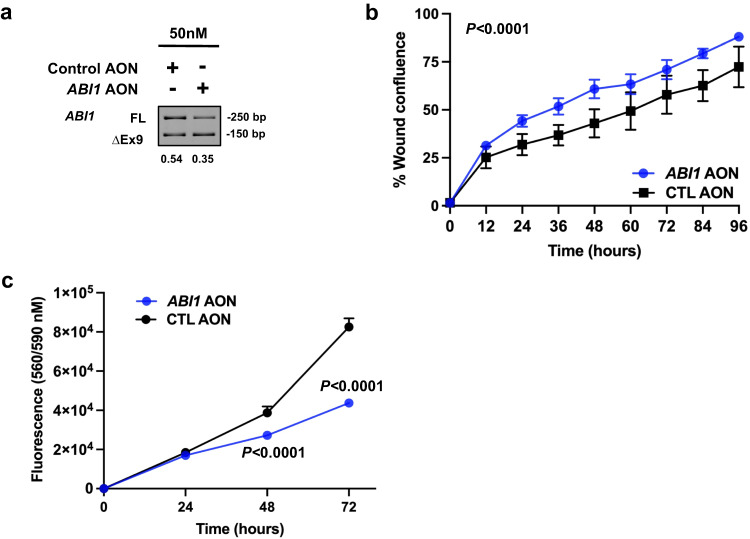
*ABI1* isoform-switching promotes migration in Panc1 cells. Splice-switching antisense oligonucleotides (AONs) were designed to target *ABI1* exon 9 in pre-mRNA transcripts to enrich for processed transcripts lacking the exon. Panc1 cells transfected with 50 nM *ABI1 AON* show a similar ∆PSI observed with RBFOX2 depletion (**a**). Panc1 cells treated with 50 nM *ABI1* AON migrated significantly faster than cells with 50 nM control AON using a wound healing assay (**b** *P* < 0.0001, DF = 8, F = 8.575, two-way ANOVA) but showed significantly decreased cellular proliferation compared to cells transfected with a control AON (**c** *P* < 0.0001, t = 11.93, DF = 24 (48 hrs) and t = 40.49, DF = 24 (72 hrs), two-way ANOVA). Data in panel **b** is representative of three independent experiments generated across three cell passages, with 4–6 technical replicates plated per line per experiment. Data in panel **c** is representative of three independent experiments generated across three cell passages, with 6–8 technical replicates plated per line per experiment. Data are presented as mean values +/- SD. Statistical analysis was performed using PRISM and the *P*-values reported for the indicated statistical tests are shown. Source data are provided as a Source data file.

To further investigate the biological role of RBFOX2-mediated *ABI1* exon 9 skipping to promote pancreatic cancer progression, we induced *ABI1* splice-switching in Panc1 cells using an *ABI1* AON to mimic the *ABI1 ∆*PSI observed with *RBFOX2* knockdown (Fig. 8a). We observed a significant increase in cell migration in Panc1 cells transfected with 50 nM *ABI1* AON compared to 50 nM control AON in a wound healing assay (Fig. 8b, overall adj. *P* < 0.0001, two-way ANOVA) and a significant reduction in cellular proliferation (Fig. 8c, overall adj. *P* < 0.0001, two-way ANOVA), consistent with our hypothesis that exon skipping of RBFOX2 target exons promotes cellular migration. These data demonstrate the *ABI1* ∆Ex9 splice form influences cellular growth and migratory potential in PDAC cells, and that RBFOX2 loss promotes the redistribution of ABI1 to the cell periphery and changes in the actin cytoskeleton that may be associated with RBFOX2-induced deregulation of cell migration.

## Discussion

Alternative splicing is an integral biological process promoting cellular plasticity, differentiation, and transformation. Recent pan-cancer analyses of splicing signatures suggest alternative splicing is a cancer hallmark and that cancers of epithelial origin exhibit conserved splicing events likely important for cancer progression^9,10^. Oncogenic mutations in splicing regulators, including SF3B1 and U2AF1, are therapeutic liabilities in leukemias and lymphomas,^56^. However, splicing regulators are rarely mutated in pancreatic cancer. Our forward genetic screen in a GEMM model of pancreatic cancer identified a significant enrichment of RNA binding proteins (RBPs) implicated in PDAC progression^21,22^ with predicted loss of function based on the transposon insertion pattern within the coding region. The human homologs of the top three RNA binding proteins identified in our screen, MBNL2, MBNL1 and RBFOX2, are differentially regulated in cancer transcriptionally and post-transcriptionally through alternative splicing^13,37^.

In this study, we showed RBFOX2 is an important regulator of alternative splicing in PDAC and that loss of nuclear RBFOX2 promoted increased tumor growth and metastasis in vivo. *RBFOX2* transcription is upregulated in PDAC compared to normal pancreas, with a significant increase in *RBFOX2* transcripts in the basal subtype, consistent with transcriptome data previously published for PDAC^31^. Data from our studies and available e-CLIP data from ENCODE ^43^ suggest RBFOX2 in part self-regulates its alternative splicing of exon 10, which encodes the nuclear localization signal in mature transcripts from the pancreas. *RBFOX2* exon 10 skipping is predicted to reduce levels of nuclear RBFOX2. While exon 10 is required for RBFOX2 nuclear localization, our data suggest regulation of RBFOX2 nuclear abundance in PDAC is complex and involves mechanisms in addition to splicing. Using TMAs from resected patient samples, we found RBFOX2 abundance and cellular distribution is heterogeneous within tumor cores and is enriched in acinar cells and early neoplastic lesions within adenocarcinoma. While we were unable to evaluate RBFOX2 abundance in PDAC liver metastatic samples, we did observe reduced RBFOX2 protein in cell lines derived from metastatic PDX models. Using a series of cell lines replete and depleted for RBFOX2, we demonstrated downregulation of RBFOX2 promotes cell migration and invasion, while RBFOX2 nuclear repletion reduces migratory capacity. Further, RBFOX2 depletion promotes tumor growth and metastasis to the liver and mesentery in orthotopic mouse models. Recent work from Jbara et al.^57^ support our ex vivo and in vivo characterization of RBFOX2 function in pancreatic cancer cells. Using metastatic seeding experiments in mice, they demonstrated RBFOX2-deficient cells more efficiently colonize the lung than RBFOX2 replete cells. We also observed increased colonization of the lungs through tail vein injection of RBFOX2-depleted KPC cells compared to RBFOX2-replete KPC cells in an immunocompetent mouse model. Together, these functional data provide strong evidence that RBFOX2 deficiency plays a biological role in PDAC progression.

Using expression arrays, we defined differentially spliced exons controlled by RBFOX2 abundance in cell lines and in orthotopic tumors. These alternatively spliced transcripts encode a network of protein-protein interactions (PPI) surrounding Rac1, a known player in cell invasion^58–60^. Jbara et al. defined similar splicing events in their recent study of alternative splicing in pancreatic cancer^57^ and showed that RBFOX2-mediated alternative splicing of the myosin phosphatase RHO-interacting protein *MPRIP* is associated with PDAC metastases. Interestingly, a subset of these RBFOX2 target exons is preferentially excluded across multiple cancers, including lung, breast, and colon^6–10^; further, these RBFOX2 target exons were shown to be alternatively spliced in ovarian and breast cancers in response to RBFOX2^8^. Here, we showed RBFOX2 target exons are differentially spliced between normal pancreas and pancreatic cancer, suggesting that alternative splicing of these exons is an integral part of pancreatic cancer progression. We further showed that RBFOX2 target exons have significantly lower PSI in the classical subtype of PDAC, which suggests alternative splicing regulation of these exons may have context-dependent effects on protein function and disease development. Specifically, we investigated the biology of ABI1 isoforms in PDAC progression and showed that alternative splice forms of ABI1 differentially regulate migratory potential of PDAC cells. ABI1 is known to be upregulated in cancer^61–63^, and we identified *Abi1* in a forward genetic screen in a PDAC GEMM^21,22^. In this study, we showed that ABI1 protein is redistributed to the cell periphery in RBFOX2-deficient cells and specifically in actin rings at the cell membrane observed only in the absence of RBFOX2. The nature of these ring structures, which indicate a change in actin cytoskeleton organization in the absence of RBFOX2, requires further investigation. The appearance of fewer actin stress fibers in RBFOX2-deficient cells may account for the increased cell migration and invasion observed in vitro and the increased frequency of liver metastasis observed in orthotopic models. We utilized splice-switching AONs to demonstrate the ABI1∆Ex9 splice form promotes cell migration in the presence of RBFOX2. Importantly, RBFOX2 regulates alternative splicing of other cytoskeletal remodeling proteins that may interact with ABI1 to promote PDAC cell migration and invasion. DIAPH2, also alternatively spliced by RBFOX2, was recently shown to interact with the C-terminal SH3 domain of ABI1^64^ and both ABI1 and DIAPH2 have been shown to have important roles in invadopodia formation in breast cancer^65^. The functional relationship among RBFOX2-mediated exon splicing events warrants further investigation to understand how these splicing events cooperate to promote pancreatic cancer progression.

Importantly, other splicing factors are likely to contribute to the complex regulation of exon splicing in pancreatic cancer. RBFOX2 depletion and repletion experiments demonstrated the role of RBFOX2 in regulating splicing of transcripts of the small GTPase *ECT2*, removing one of two exons encoding the N-terminal BRCT domain which negatively regulates ECT2 GTP binding^66^. This splicing event is conserved, which may contribute to ECT2 oncogenic function in breast and other cancers^67–70^. In PDAC, the *ECT2* PSI was less than 0.5 across all cell lines assayed, indicating there is likely functional redundancy between RBFOX2 and other splicing factors regulating *ECT2* splicing. In addition, several of the exon splicing events we identified with RBFOX2 depletion are alternatively spliced by the RBP QKI in breast cancer cell lines^71^ and RBFOX2 and QKI intronic binding sites reside in proximity for some alternatively spliced exons^72^. *QKI* expression is low across most of the PDAC cell lines we assayed and its functional role in PDAC splicing regulation has not been studied. Other splicing factors, including MBNL1, identified in our forward genetic screen^21,22^ may also play a role in regulating alternative splicing in PDAC. Alternative splicing of *KIF13A*, an event we identified in our RBFOX2 depletion studies, was previously shown to be affected by MBNL1 abundance^14^.

While analyses to date have identified few splicing regulators with prognostic implications in PDAC, the incidence of exon splicing events conserved across PDAC and other cancers suggests the processes regulated by alternatively spliced transcripts are integral to cancer progression. PDAC cells that retain both epithelial and mesenchymal-like programs demonstrate increased metastatic potential in mouse models^73,74^. Our results from *KRAS* mutant PDAC cell lines linking RBFOX2 depletion in mesenchymal cells with enhanced exon skipping and metastatic phenotypes suggest that a shift towards a more epithelial-like splicing signature can promote PDAC metastasis. Defining mechanisms regulating RBFOX2 splicing function in cancer will be critical to further our understanding of the context dependency of the RBFOX2 splicing program driving metastasis in PDAC.

## Methods

The research reported in this manuscript complies with all relevant ethical regulations and policies of Moffitt Cancer Center and the governing Institutional Regulatory Board for de-identified patient samples. BSL-2 and ABSL-2 experiments were conducted under Institutional Biosafety protocols PROTO2020-009 and PROTO2023-024 and IACUC protocols IS00008009 and IS00011557 administered by the University of South Florida and Moffitt Cancer Center.

### Cell culture

4039, Panc1, MiaPaCa2, Panc02.03, BxPC3, PL45, and HPNE cell lines were obtained from ATCC and were propagated according to ATCC recommended conditions. 8902 cells were obtained from the DSMZ-German Collection of Microorganisms and Cell Cultures GmbH and propagated in DMEM with 10% FBS. MDA-PATC cell lines (PATC124, PATC43, PATC153, PATC107, PATC53, PATC102, PATC108, PATC148) were obtained from MD Anderson Cancer Center and cultured in RPMI media supplemented with 2 uM glutamine and containing 100ug/mL primocin (Invivogen, Cat. no. ant-pm-1). Cell lines were routinely tested for mycoplasma and validated using STR profiling and mutational analysis.

### Vectors and lentivirus production

pGIPZ lentiviral vectors containing shRNAs directed against *RBFOX2* (Dharmacon) were purchased as lentiviral preparations (Cat. no. VGH5523-200225575, VGH5518-200275809, VGH5518-200230034). Additional shRNAs against *RBFOX2* were designed using splashRNA ^75^ and cloned into the lentiviral PRRL vector as described ^76^. See Supplemental Table 1 for sequences. DDK-MYC tagged *RBFOX2* cDNAs were purchased from Origene in pCMV6-Entry vector (Cat. nos. RC206846 for RBFOX2-V1 and RC222907 for RBFOX2-V4). cDNAs were first subcloned into pLenti (Origene Cat. no. PS100092) maintaining the DDK-MYC tag and then into Pentr4FLAG (Addgene Cat. no. 17423) before cloning into the lentiviral DOX-inducible destination vector pLIX403 (Addgene Cat. no. 41395) using LR Clonase II. Lentiviral DNA constructs were packaged using ABMgood packaging mix (Applied Biological Materials Cat. no. LV053) and transfected into 293 FT cell lines using jetPRIME transfection reagent (Polyplus, Cat. no. 89129-922). Lentivirus was concentrated by ultracentrifugation in clear ultracentrifuge tubes (Beckman Cat. no. NC9146666) spun in a Beckman Ultracentrifuge OPTIMA L 70 K rotor (SW32 TI) at 23000 rpm for 2 hours at 4^o^C. Lentiviral titers were obtained using ABMgood qPCR Lentivirus Titer Kit (Applied Biological Materials, Cat. no. LV900). All vectors were confirmed by restriction digest and Sanger sequencing. Transductions were performed according to Dharmacon guidelines with 8 ug/mL polybrene and stably selected using puromycin.

### CRISPR RBFOX2 sgRNA, siRNA, and AON transfections

RBFOX2 sgRNA was introduced using nucleofection into Panc1 (Lonza SE kit, Nucleofector X Unit) or MiaPaCa2 cells (Lonza V kit, Nucleofector IIB Unit). siRNAs were purchased from Horizon Discovery to target *RBFOX2* (on-TARGET siRNA pool, Cat. no. M-020616-02-0005), or siControl (NTC siGenome, Cat. no. D-001210-01-05). Antisense oligonucleotides were purchased from TechNoA for splice-switching: *ABI1* (TechNoA tNOA-20M-hABI1-01, #1852, #1853). siRNAs or AONs were transfected using Lipofectamine RNAiMax reagent (ThermoFisher Scientific, Cat. No. 13778150) according to the manufacturer’s instructions; oligos were incubated with Lipofectamine 20 minutes at room temperature prior to addition to culture media.

### Cellular assays

Proliferation assays were performed for adherent cells using Cell Titer Blue (Promega, Cat. no. G8080) and quantified using Promega Glomax Discover plate reader, software version 3.2.3, after four hours of incubation with the reagent at 37 degrees Celsius, 5% O_2._ Cell lines were induced with 250 ng/mL doxycycline 72 hours prior to initiation of assays. For experiments with AONs, cells were plated for proliferation assays 24 hours post-transfection. Wound healing assays were performed using the Incucyte Live-Cell Analysis System (Incucyte Zoom, Sartorius). Cells were plated in 96-well plates (Essen Bioscience, Cat. no. 4379) overnight to form a confluent cell layer. 6 replicate wells were plated per cell line per assay. 16 hours post-plating, cells were treated with 10ug/mL mitomycin C (Cayman chemical, Cat. no. 11435-5) for 2.5 hours to inhibit cellular proliferation. A scratch was made using Incucyte® Woundmaker Tool (Cat. no. 4563) and cellular migration was assayed 72-96 hours. Percent wound confluence was imaged using Incycyte Zoom software version 2021 A. Where applicable, pLIX403 and PRRL constructs were induced using 250 ng/mL doxycycline for three days prior to plating for cellular assays and treatment continued throughout the experiment. For migration assays with AONs, cells were transfected directly in the 96-well plates (16 hours post-plating) and a scratch was made 24 hours post-transfection. We titrated the numbers of cells plated such that at the time of the scratch there was a confluent monolayer of cells. Chemotactic invasion assays were performed using 4039 cells modified for RBFOX2 expression with the Incucyte Live-Cell Analysis System (Incucyte Zoom). Cells were serum-starved in media containing 1% bovine serum albumin (VWR, Cat. no. 0332-100 G) for protein supplementation 16 hours prior to plating. Cells were treated with Cell Trace Far Red (Invitrogen, Cat. no. C34572) to detect nuclei according to the manufacturer’s instructions prior to plating cells in serum-free media with 1% BSA on top of Matrigel-coated wells of Incucyte® Clearview 96-well plates (Corning Matrigel Growth Factor Reduced (GFR) Basement Membrane Matrix (Fisher Scientific, product no. CLS356231). 3D invasion into the matrix was monitored for 96 hours and percent invasion was calculated using the Integrated Incucyte® Chemotaxis Analysis Software Module (Sartorius), version 2021 A.

### Western blotting

Whole cell protein lysates from adherent or non-adherent cell cultures were isolated in RIPA buffer (100 mM Tris pH7.5, 300 mM NaCl, 2 mM EDTA, 2% NP-40, 2% Sodium deoxycholate and 0.2% SDS including protease inhibitors (Aprotinin (TOCRIS Bioscience Cat. no. 4139/10), Leupeptin (Fisher Scientific, Cat. no. 501685874), Sodium Orthovanadate (New England Biolabs, Cat. no. p0758s), Pepstatin (Cat. no. BP267110), Sodium Fluoride (Sigma, Cat. no. 201154-5 G), AEBSF (Fisher Scientific, Cat. no. 40160000-2) and nuclease for cell lysis (Life Technologies, Cat. no. 88700) directly from tissue culture plates by scraping. Protein concentrations were determined using a BCA assay (Pierce, Catalog no 23227). Protein extracts were separated using SDS page gels with 4X Laemmli loading buffer and transferred onto nitrocellulose (BioRad Cat. No. 3500601) using a wet transfer apparatus and blocked using either LI-COR Intercept (TBS) Blocking Buffer (LI-COR, Cat. No. 927-60001) or 5% milk in TBST (20 mM Tris pH 7.5,150 mM NaCl with 0.1% Tween-20). Antibodies were validated according to the manufacturer’s instructions and recommended cell lines and/or by using MCF7 cells as a positive control for RBFOX2 and MB-MDA-231 cells as a positive control for ABI1 expression in conjunction with PDAC cell lines with RBFOX2 or ABI1 knock-down or over-expression of exogenous constructs. Antibodies were diluted in the appropriate blocking buffer to visualize the following: RBFOX2 ((RBM9) Bethyl Labs, Cat. No. A300-864A, 1:2000); ABI1 (Cat. no. 39444, 1:500), Vimentin (Cat. no. 5741 S, 1:1000), CDH1(Cat. no. 3195 S, 1:1000) and Rac1 (Cat. no. 4651, 1:500) from Cell Signaling; GAPDH (Santa Cruz Cat. no. SC-69778, 1:5000); Beta-Actin (Cat. no A5441, 1:20 K), Flag-M2 (Cat. no. F3165, 1:5000) and Histone H3 (Cat. no. 05-928, 1:2000) from Sigma. LI-COR Secondary antibodies for Infrared were IRDye 800cw anti-Rabbit (Cat. no. 926-32211, 1:5000) and IRDye 680RD anti-Mouse (Cat. no. 925-68070, 1:5000); HRP-conjugated anti-Rabbit (Vector Laboratories, Cat. no. PI-1000, 1:5000) and HRP-conjugated anti-Mouse (Jackson Immuno Research Labs, Cat. no. 115-035-003, 1:5000). HRP secondaries were developed using Pico chemiluminescent reagent (Pierce, Cat. no. PI34577). Gel images were acquired using LI-COR Odyssey CF software version 1.0.36 and quantitated using LICOR ImageStudioLite Version 5.2.5.

### Cell fractionation

Cells were harvested from cultures grown to 80% confluency. 2-3 million cells resuspended in cold PBS were pelleted by centrifugation at 850 g for 5 minutes at 4 degrees, then resuspended in 80 uL cold NETN buffer for 10 minutes on ice (50 mM TRIS HCl pH 8.0, 250 mM NaCl, 0.05%, NP40, 5 mM EDTA, 1 mM DTT, and protease inhibitors) to isolate the cytoplasmic fraction. Lysed cells were centrifuged at 10,000 *g* for 5 minutes at 4 degrees and the supernatant containing the cytoplasmic fraction was transferred to a new tube on wet ice. The nuclear pellet was washed twice with cold PBS, then resuspended in 60uL of complete RIPA buffer with protease inhibitors and nuclease. Lysates were incubated on ice for 40 minutes, with vortexing every 10 minutes to ensure complete digestion. Protein concentrations were determined using BCA assay (Pierce, Catalog no 23227). Exogenous Flag-tagged and endogenous RBFOX2 were probed in each cell fraction on an 8% SDS-PAGE gel. Histone H3 and GAPDH were used as markers of the nuclear and cytoplasmic fractions, respectively.

### Rac1-GTP pulldown assays

3T3 cells stimulated with PDGF were used to validate the Rac1 pulldown assay. Panc1 and 4039 cells grown to 80% confluency over 3 days were stimulated for 30 minutes with 30 ng/mL PDGF (Peprotech Cat. no. 100-00AB), then placed immediately on ice, washed once with PBS, then lysed and immediately scraped (Lysis Buffer: 50 mM Tris pH 7.5, 10 mM MgCl2, 0.5 M NaCl, 2% NP-40 and protease inhibitors). The cell lysate was clarified by centrifugation at 10,000 x *g*, 4^o^C for 1 minute, then transferred to a prechilled tube and quantitated by Bradford assay. 300 ug of protein in 600 uL of lysis buffer was used for the GTP pulldown with 20 ul of PAK beads (Sigma, Cat. no. 14-325) incubated for one hour. PAK beads were centrifugated at 5000 x *g* at 4^o^C for 1 minute and washed once with 500 uL wash buffer (25 mM Tris pH 7.5, 30 mM MgCl_2_, 40 mM NaCl). Washed beads were spun down and the bead pellet was resuspended in 20uL of 2x Laemmli Sample Buffer (BioRad, Cat. no. 161-0737) with 2-Mercaptoethanol (ICN Biomedicals, Cat. no. 806443). GTP-bound Rac1 and total Rac1 were visualized by western blot.

### PDAC TMAs

Pancreatic cancer TMAs were previously constructed at Moffitt Cancer Center under Moffitt’s Total Cancer Care (TCC^TM^), an institutional review board (IRB)-approved general biobanking protocol (MCC14690/IRB 104189 and MCC13579/IRB 101642). Under TCC, patients provide prospective written consent for biospecimen collection. Access to de-identified pancreatic cancer TMAs used in this study was granted under MCC50295.

### Immunohistochemistry and immunocytochemistry

Analysis of RBFOX2 nuclear abundance was performed using immunohistochemistry on formalin-fixed, paraffin-embedded mouse tissue (pancreas and liver) with RBFOX2 antibody (RBM9 IHC00199, Bethyl Labs, 1:600) and on human pancreas tissue microarrays with RBFOX2 antibody (RBM9 IHC00199, Bethyl Labs, 1:2000) using a Ventana Tissue Processor. Mouse pancreas spleen and human breast tissue were used as positive controls. Histological images were captured using the Zeiss Axio Imager.M2 with Zeiss Axiocam 503 color camera and Zeiss Zen Blue software version 3.5. TMA slides were scanned using the Aperio™ ScanScope AT2 (Leica Biosystems, Vista, CA) with a 20x/0.8NA objective lens. Images were stored in Aperio’s Eslide manager software where each TMA image was segmented into individual cores using the software’s TMA lab module. The individual core images were exported as uncompressed Tiff files and imported into the Definiens Tissue Studio v4.7 (Definiens Inc, Germany) for segmentation and analysis. In Tissue Studio, a machine learning algorithm was used to segment each TMA core image into Tumor and Non-Tumor (mostly Stroma) areas. A minimum size threshold setting was used to further refine this segmentation. Next, a nucleus detection algorithm was used to find all nuclei within each segmented area. Detected objects that were smaller than 10 microns squared were not considered as nuclei. This nuclei detection was used for cell simulation using a simple growth algorithm of 5 microns. IHC stain intensity within the nucleus compartment was binned into four categories Negative, Low, Moderate, or High based on thresholds set using positive and negative stain controls included with this batch of slides. The data for each image was exported into Microsoft Excel where positive cell density, percent positive, and H-score were calculated using the raw data. Mean IHC staining intensity for the cell, nucleus, and cytoplasm compartments was also exported. To visualize cellular morphologies and protein distribution for live cells grown in culture, 3E + 04 cells filtered through 70uM strainers were grown in complete media on acid-washed coverslips (Fisher, Cat. no. NC0706236) coated with 2% unlabeled bovine gelatin (Sigma, Cat. no. G1393) overnight and fixed with 4% paraformaldehyde. Cells were permeabilized with 0.5% Triton-X in PBS for 5 minutes and blocked with 5% BSA in 0.2% Triton-X. Cells were incubated with Alexa Fluor 594-labelled phalloidin (ThermoFisher, Cat. no. A12381, 1:1200) according to methods previously described ^77^. To visualize ABI1 isoform localization, cells were grown as described and incubated with phalloidin and ABI1 antibody (Invitrogen PA5110991, 1:250) with Alexa-488 conjugated secondary (Invitrogen A11034, 1:1000). Cortactin was visualized using ab [4F11] (Abcam, Cat. no. ab33333, 1:250) with Alexa-546 conjugated secondary (Invitrogen A10040, 1:1000). Cells were stained with Hoescht 33324 diluted 1:8000 (stock 10 mg/mL, Invitrogen H3570) for 10 minutes and coverslips were mounted to slides using Vectashield mounting media plus antifade (Vectashield, 103881-344). Immunofluorescence imaging was performed using the Leica TCS SP8 STED 3X and LAS X software. Analysis of ABI1 intensity at the cell periphery was done using Image J (version 1.53t) with a custom ImageJ macro as previously described^78^.

### Splicing analysis using arrays

Transcriptome profiling to define RBFOX2 splicing signatures from 4039, Panc1 and MiaPaCa2 cell line pairs replete or depleted for RBFOX2 was performed using human Affymetrix Clariom D Arrays (Life Technologies, Cat. no. 902922) with the standard input kit run on the Applied Biosystems GeneChip 3000 instrument. Total RNA was extracted from whole cells or from pancreatic tumors using Qiagen rNeasy Mini kit Dnase I treatment (Cat. No. 74106). Samples were prepared with the GeneChip WT Plus Reagent kit according to manufacturer’s guidelines. Differential exon usage analysis between RBFOX2 “high” (control) and RBFOX2 “low” (knockdown) was performed using Affymetrix Transcriptome Analysis Console (TACX) software version 4.0.2 using the r1.GENE.CDF file with na36.hg38.a1 probesets. Analysis of cell lines and tumor samples was performed independently.

### Gene expression and splicing analysis from human RNA-seq data

Aligned data (BAM format) were downloaded for data sets EGAD00001004548 and EGAD00001003584 through MTA access with the University of Ontario. CPTAC BAM files were accessed through Genomic Data Commons (https://gdc.cancer.gov) using dbGAP. Within each data set, gene-level and exon-level expression count data were generated for each sample using the featureCounts command from the Subread software package ^79^ against GRCh38 v84 annotation and normalized to Transcripts Per Million (TPM). The Python script dexseq_prepare_annotation.py provided with the R package DEXSeq^80^ was used to translate Ensemble v84 GTF file to a GTF file with collapsed exon counting bins. The same GTF file was later used to calculate exon-level expression and the Percent Spliced In (PSI) index. PSI was defined as the ratio between reads including or excluding an exon and calculated following a previous published protocol^39^. For a given exon, PSI = 1 means this exon is 100% spliced in; while PSI = 0 means this exon is 100% spliced out. Pearson and Spearman correlations between RBFOX2 expression (log2 TPM) and target exon PSI were calculated using R 4.0.3. Permutation testing was performed to compare the average absolute correlation of the exons of interest observed in the data to the average absolute correlation of a randomly selected collection of same number of exons (repeated 10,000 times). An empirical *p*-value was calculated as the proportion of permutations in which the average correlation generated from randomly selected exons was greater than the observed average correlation. Splicing analysis from TCGA PAAD RNA-seq data was interrogated using SpliceSeq^38^. For data set E-MTAB-6830^35^, FASTQ data were downloaded from the NCBI SRA, and aligned to the Human GRCh38 reference genome using STAR (version 2.7.3a)^81^. Gene-level count data were generated using featureCounts^79^ based on GRCh38 v99 annotation. For both data sets, count data were converted to log_2_ counts per million, and processed in the R computing environment with the Voom normalisation procedure from the limma software package^82^. Limma was also used for all differential expression analyses. For EGAD00001004548 and E-MTAB-6830 datasets, Moffitt subtype information was generated per-sample using the following procedure: data for the 28 genes used to define the Moffitt subtypes were extracted from each data set, and consensus clustering was then used to separate the samples into two distinct clusters (Basal-like and Classical). Cluster identity was confirmed by checking survival associations (in each case the Basal-like subtype was associated with worse prognosis, as per the original paper)^33^. For CPTAC data, we utilized the published subtype designations^34^. Additional clinical information was obtained for the EGAD00001004548 data set by matching sample IDs to those available from the ICGC PACA-CA project (matches were found for 197 samples).

### Splicing analysis using real-time PCR

1 ug RNA was used to prepare cDNA from PDAC cell lines replete or depleted for RBFOX2 using the Vilo cDNA kit (Invitrogen Cat. No. 11755050). PCR amplification was performed with Roche Taq (Cat. No. 11146173001) and PCR products were visualized on 12% acrylamide gels stained with 5 ug/mL ethidium bromide. Gel images were captured using Licor Odyssey FC version 1.0.36 and PCR bands quantified using Image Studio Lite software version 5.2.5. A minimum of 3 splicing PCRs were run per cell line per target exon. PSI calculations were performed as previously described^8,14^. Primer sequences are provided in Supplemental Table 1.

### Mice

Human 4039 (5 × 10^4^) and Panc1 (1 × 10^5^) cells were prepared in serum-free media with 0.1% Matrigel and orthotopically introduced into the tail of the pancreas of male and female NSG recipient mice (JAX 005557) according to methods described previously ^83^. Murine KPC (1 × 10^3^) cells were orthotopically introduced into the pancreas or injected via tail vein into C57Bl/6 J mice (JAX 000664). Isogenic cell lines (knockdown vs. control) were matched for cell passage, implanted into litter mates at 8–16 weeks of age and mice aged for 45 days. Mice were maintained on a 12/12 light:dark cycle, with food and water ad libitum. Primary tumor volumes, metastatic incidence and lesion sizes were measured at necropsy. No differences were observed between males and females and the data from both sexes was combined for statistical analysis of tumor volumes and determination of metastatic incidence and lesion sizes. Pancreas tumors were formalin fixed and embedded with the spleen attached for orientation. A portion of the tumor was snap frozen for molecular analysis. Metastatic lesions greater than 2 mm were formalin-fixed for histology. All procedures were conducted under approved IACUC protocols and following AALAC guidelines. The maximal tumor size of 20 cm in any direction or 10% of mouse body weight was not exceeded for these studies.

### Statistical analysis

Statistical analysis was carried out using R or GraphPad PRISM software 10.0.2. Measurements were taken from distinct samples except for wound healing assays, where individual wells were measured repeatedly for the duration of the assay. Individual statistical tests and test statistics are referenced within figure legends. The mean and standard deviation (depicted by error bars) are included for dot plots, and the parameters for box and whiskers plots are defined within the figure legends. Adjustments for multiple comparisons were performed where noted. Oncoprints were generated from published datasets from the Sleeping Beauty Cancer Driver Database (SBCDDB,^22^) using the cBioPortal Oncoprinter tool.

### Reporting summary

Further information on research design is available in the Nature Portfolio Reporting Summary linked to this article.

### Supplementary information

Supplementary Information

Description of Additional Supplementary Files

Supplementary Data 1

Supplementary Data 2

Reporting Summary

### Source data

Source Data

## Data Availability

The CEL files for Clariom D Exon Arrays generated in this study have been deposited in GEO under accession code GSE211435 (https://www.ncbi.nlm.nih.gov/geo/query/acc.cgi?acc=GSE211435). The publicly available microarray datasets for pancreatic tumors and normal pancreas were downloaded from GEO under accession code GSE16515 (https://www.ncbi.nlm.nih.gov/geo/query/acc.cgi?acc=gse16515)^84^ and GSE28735 (https://www.ncbi.nlm.nih.gov/geo/query/acc.cgi?acc=GSE28735) ^27^. The publicly available RNA-seq data from patient samples are available at the European Genome Phenome Archive (https://www.ebi.ac.uk/ega) for dataset IDs EGAD00001003584 and EGAD00001004548^1,49–51^. The publicly available CPTAC RNA-seq data from patient samples are available from Genomic Data Commons (https://gdc.cancer.gov) using dbGAP ^34^. The publicly available RNA-seq data from patient samples for dataset E-MTAB-6830 are available from the European Bioinformatics Institute (https://www.ebi.ac.uk) ^35^. The publicly available Sleeping Beauty Cancer Driver database is accessible here: http://sbcddb.moffitt.org^22^. Source data are provided with this paper. High-quality images for RBFOX2 IHC and confocal images can be found here 10.6084/m9.figshare.24212682. The remaining data are available within the Article, Supplementary Information or Source Data files. Source data are provided with this paper.
