# Supplementary material for: RBFOX2 deregulation promotes pancreatic cancer progression and metastasis through alternative splicing
Source: Nat Commun. 2023 Dec 19;14:8444. doi: 10.1038/s41467-023-44126-w (PMC10730836; doi:10.1038/s41467-023-44126-w)

Supplemental Figure 1.

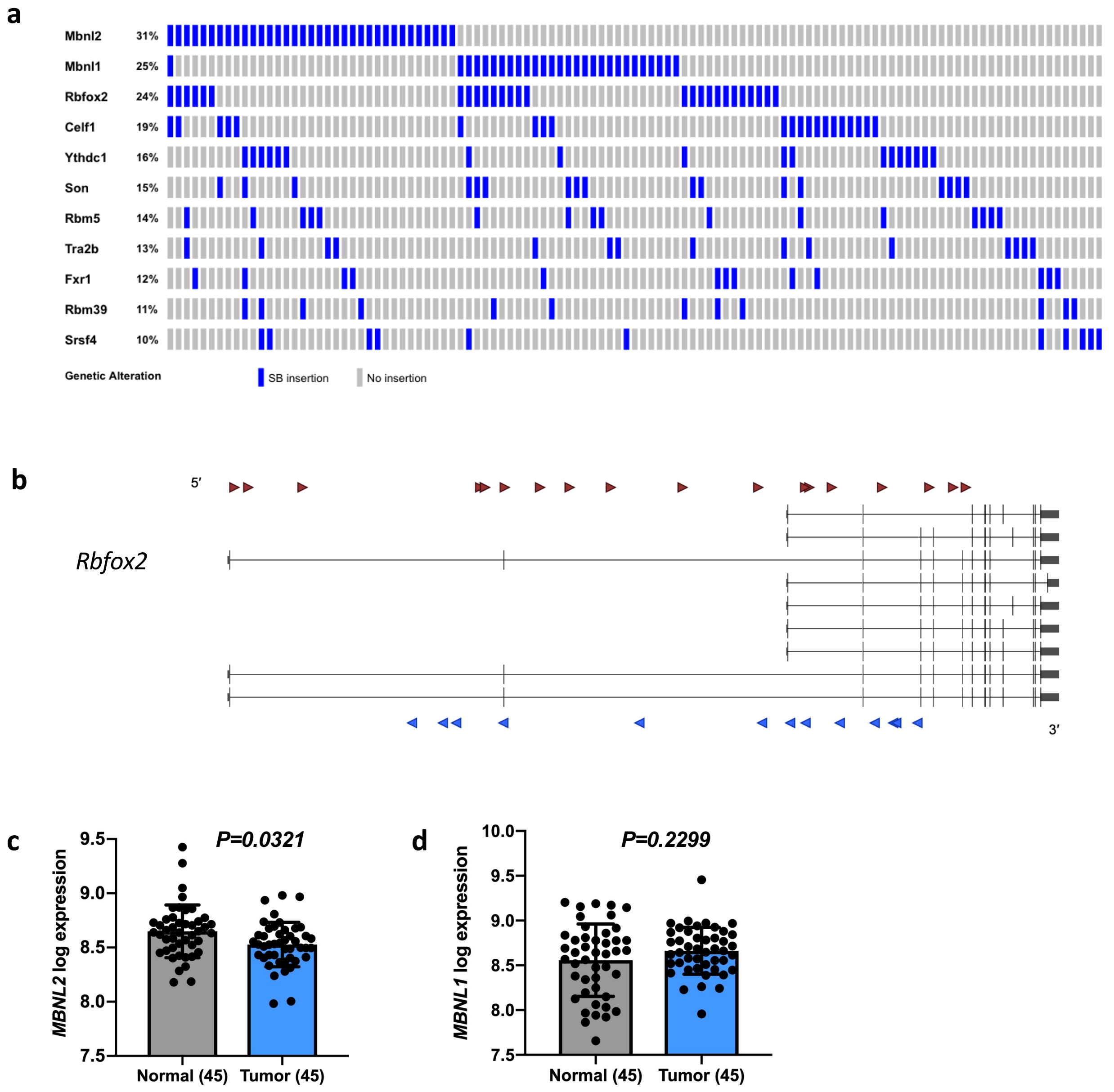

Supplemental Figure 1. Identification of *Rbfox2* as a progression driver in a Sleeping Beauty mouse model of pancreatic cancer. Progression driver genes identified in an *in vivo* forward genetic screen using *Sleeping Beauty* (SB) insertional mutagenesis in a GEMM model of pancreatic cancer [1, 2] are statistically enriched for regulation of mRNA splicing via the spliceosome. An oncoprint of the SB transposon insertion frequencies in regulators of mRNA splicing identified *Mbnl2*, *Mbnl1* and *Rbfox2* as the most frequent “hits” (**a**). Each blue bar indicates the occurrence of an SB insertion in the coding region of the indicated gene in an individual tumor. An insertion map for SB integrations in the *Rbfox2* coding region (**b**) predicted *Rbfox2* gene inactivation; red triangles indicate individual insertions in the sense direction of the coding region, while blue triangles indicate insertions in the anti-sense direction. Expression analysis of *MBNL2* in tumor (n=45) vs. normal (n=45) pancreas from GEO dataset GSE28735 [3] showed a significant decrease in expression in tumors compared to normal pancreas (**c,** FDR adj. *P*=0.0321), while *MBNL1* showed no significant difference in gene expression between tumor (n=45) and normal (n=45) pancreas (**d**, FDR adj. *P*=0.2299). Data are presented as mean values +/- SD.

Supplemental Figure 2.

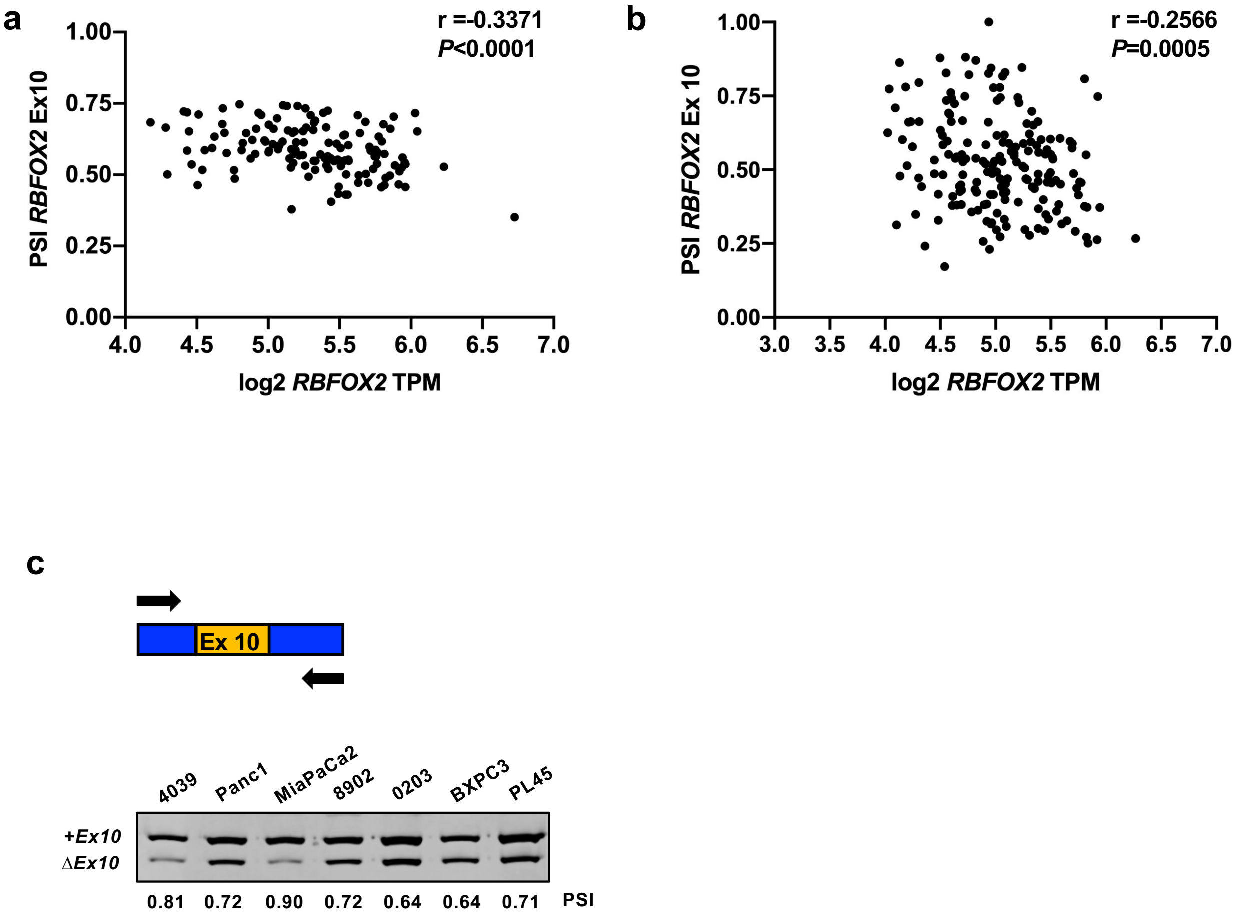

Supplemental Figure 2. *RBFOX2* exon 10 splicing is negatively correlated with *RBFOX2* expression in PDAC. *RBFOX2* exon 10 PSI is negatively correlated with *RBFOX2* expression in PDAC tumors for both the CPTAC dataset [4] (n=136 XY pairs) (**a,** Pearson's correlation *P*<0.0001, 95% confidence interval -0.4783 to -0.1789) and the EGAD00001004548 [5-8] dataset (n= 182 XY pairs) (**b**, Pearson's correlation *P*=0.005, 95% confidence interval -0.3876 to -0.1154). RT-PCR to detect inclusion of exon 10 in *RBFOX2* transcripts using primers in adjacent exons showed exon 10 is predominantly included in *RBFOX2* transcripts across PDAC cell lines, with a lower PSI in epithelial-like lines (**c**). Splicing data is representative of 3 independent experiments.

Supplemental Figure 3.

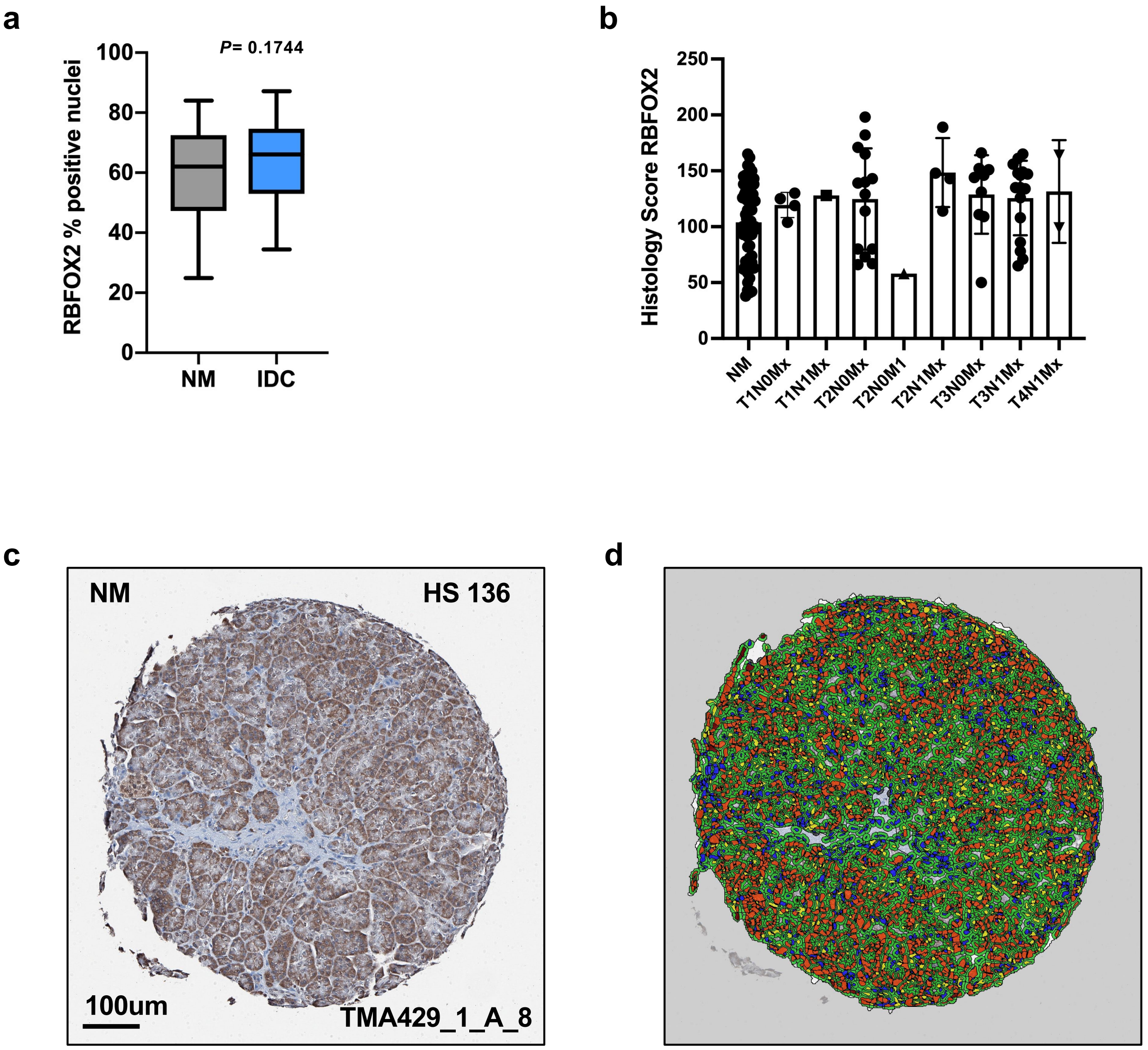

Positive/total nuclei: 3127/4102

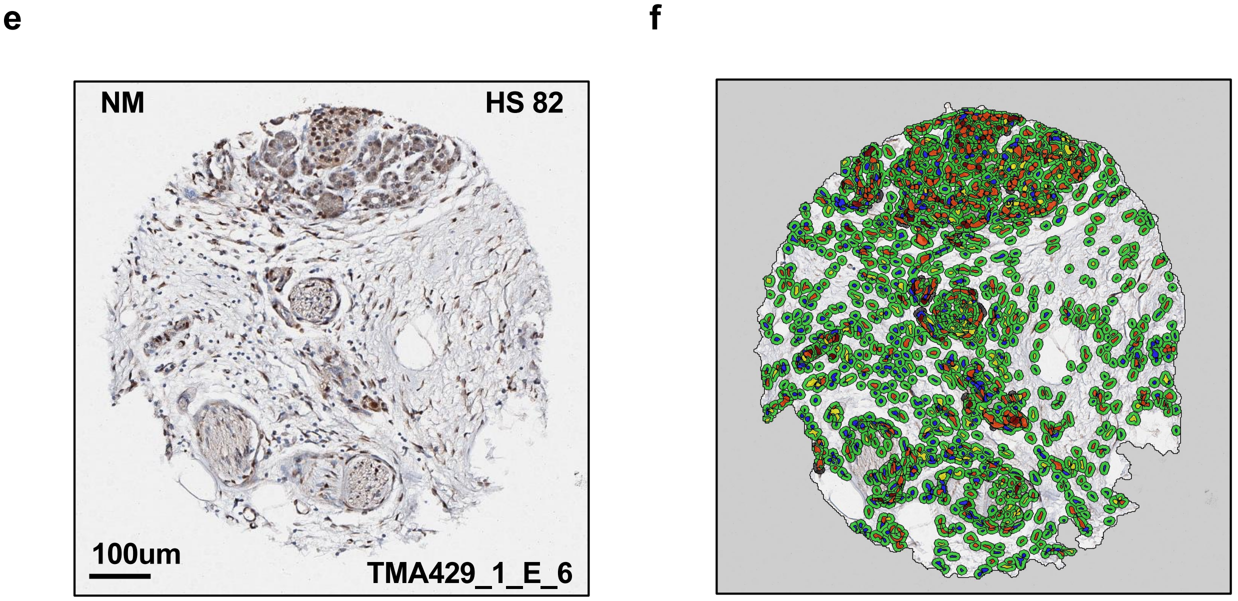

Positive/total nuclei: 921/1944

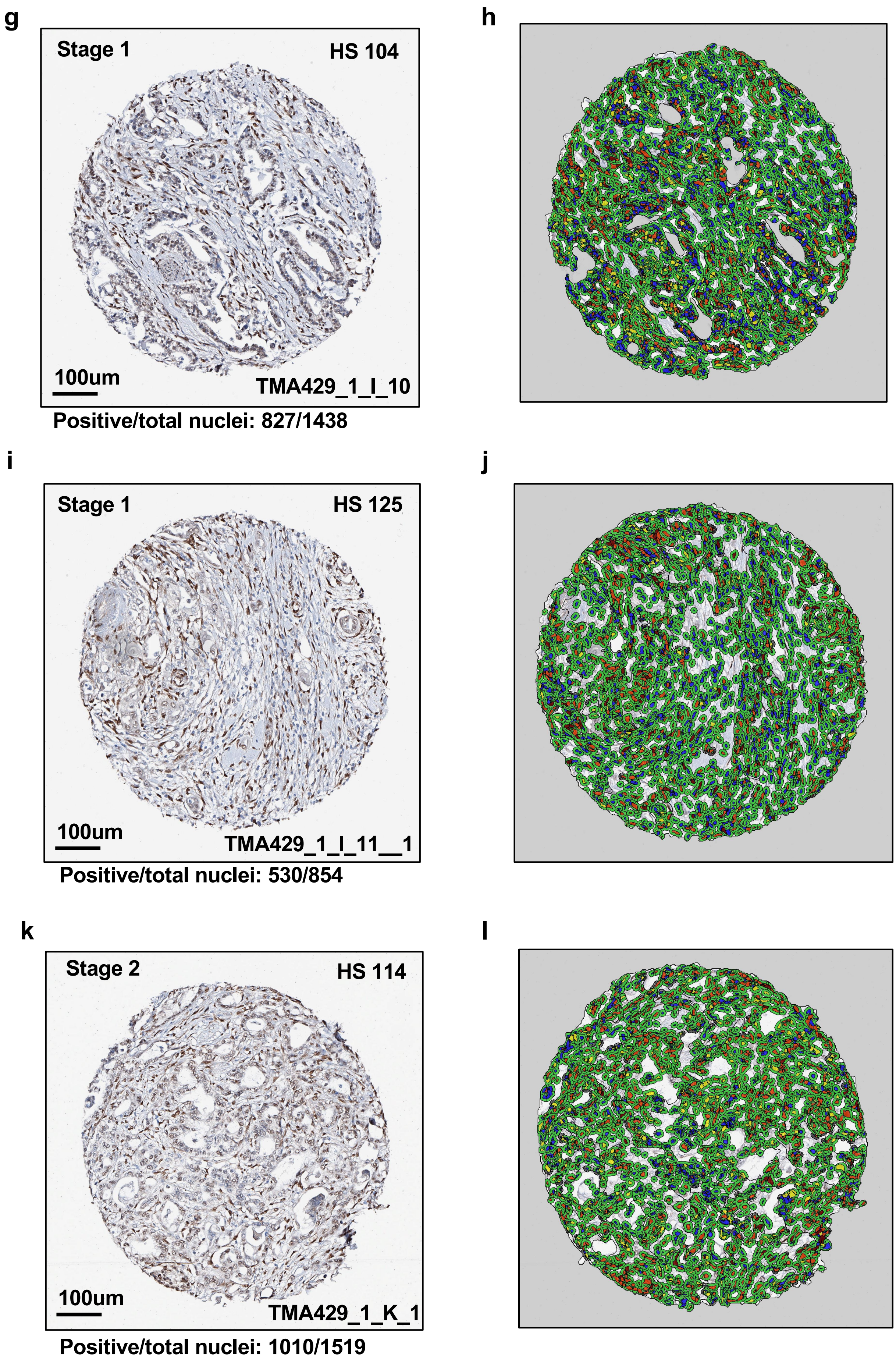

g
Stage 1
HS 104
100um
TMA429_1_I_10
Positive/total nuclei: 827/1438
h
i
Stage 1
HS 125
100um
TMA429_1_I_11__1
Positive/total nuclei: 530/854
j
k
Stage 2
HS 114
100um
TMA429_1_K_1
Positive/total nuclei: 1010/1519
l

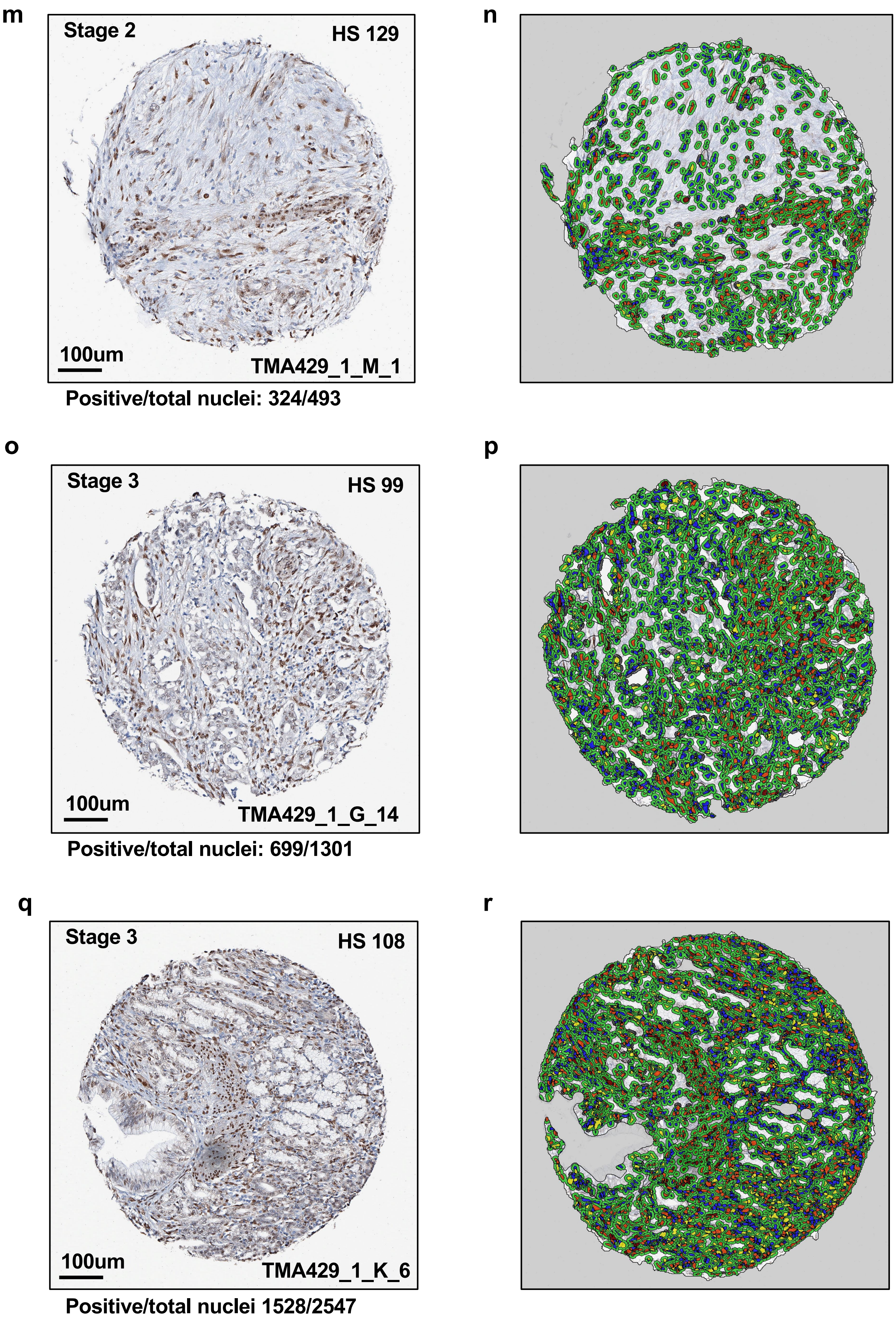
m
Stage 2
HS 129
100um
TMA429_1_M_1
Positive/total nuclei: 324/493
n
o
Stage 3
HS 99
100um
TMA429_1_G_14
Positive/total nuclei: 699/1301
p
q
Stage 3
HS 108
100um
TMA429_1_K_6
Positive/total nuclei 1528/2547
r

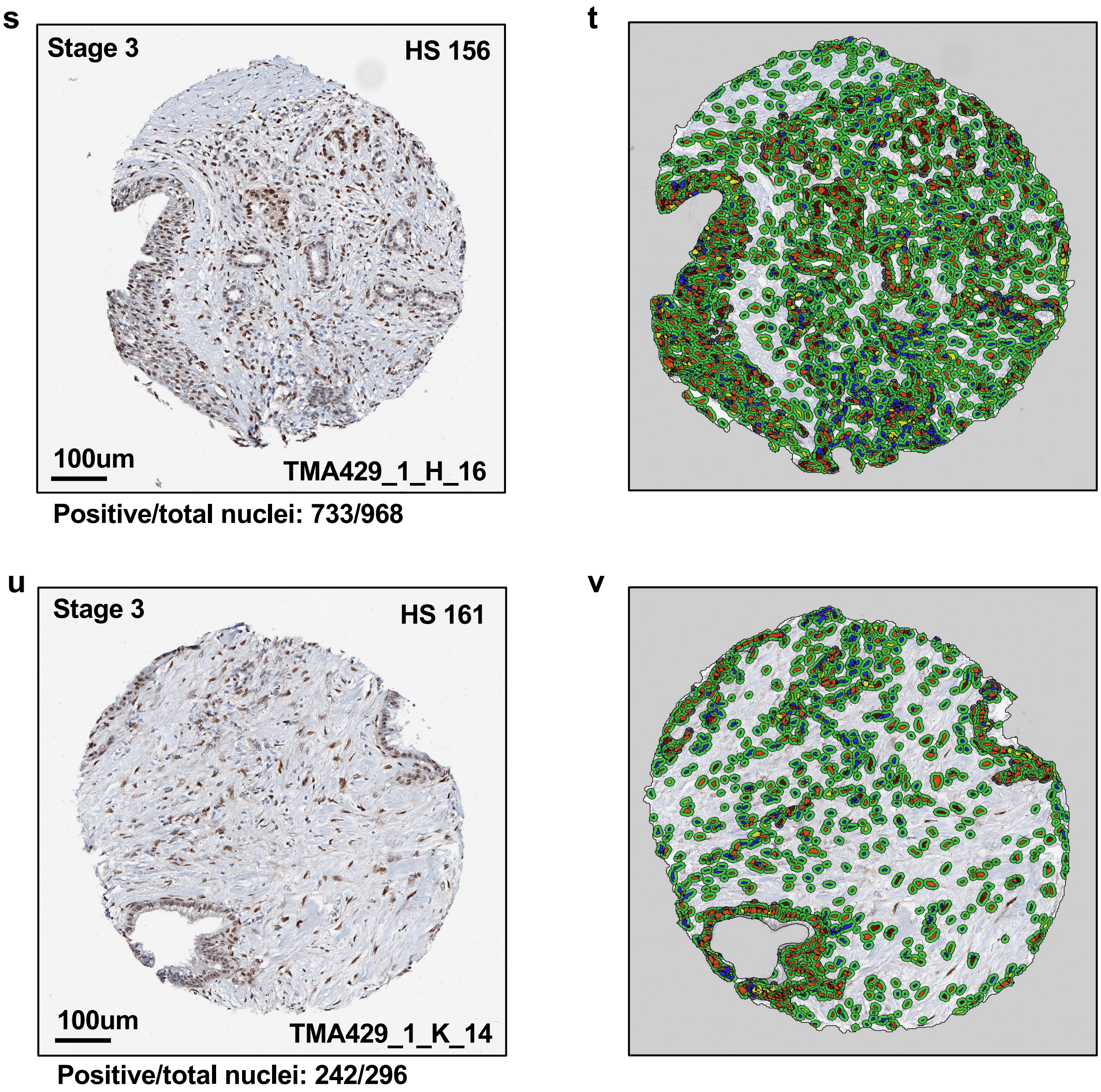
s
Stage 3
HS 156
100um
TMA429_1_H_16
Positive/total nuclei: 733/968
t
u
Stage 3
HS 161
100um
TMA429_1_K_14
Positive/total nuclei: 242/296
v

Supplemental Figure 3. Quantitation of RBFOX2 nuclear abundance in human PDAC TMAs. Population analysis of RBFOX2 nuclear abundance in TMAs from resected PDAC and non-malignant pancreas. On average, 60% of cells from non-malignant (NM) pancreas (n=60) or invasive ductal carcinoma (IDC, n=59) are positive for RBFOX2 and were not significantly different (**a**, adj *P*=0.1744, 2-tailed t-test, t=1.365, df=149). Quantitation of total RBFOX2 nuclear intensity, defined as the histology score, is plotted for non-malignant (NM) pancreas and for all stages of PDAC represented in the TMAs (**b**). A stromal filter was applied to tumor tissue cores, and the staining intensity of RBFOX2 was quantified only in tumor cells. Immunohistochemistry of RBFOX2 in tissue cores representative of NM and PDAC stages is shown (panels **c, e, g, i, k, m, o, q, s, u**) with accompanying digital images used for quantitation of the nuclear intensity (panels **d, f, h, j, l, n,p, r, t, v**) designated as histology score. For histological images, the stage, histology score (**HS**, upper right-hand corner of images), de-identified core designation and the ratio of positive to total nuclei scored is shown. For digital images, detected nuclei are outlined in green. RBFOX2 staining intensity is color-coded, where dark red=high; light red=moderate; yellow=low and blue=absent. Some cores from nonmalignant pancreas are composed of dense regions of acinar cells with relatively uniform and abundant RBFOX2 staining in most scored nuclei (**c**, 76% positive nuclei), while other NM cores show regions with stroma infiltration and early neoplastic lesions with variable RBFOX2 staining intensities (**e,** 40% positive nuclei). The nuclear intensity of RBFOX2 in tumor cells was not significantly different for stage 1 (**panels i** and **k**) and stage 2 (**panels k** and **m**), although the number of tumor nuclei per core varied. RBFOX2 histology scores and number of tumor nuclei for cores of stage 3 tumors (**panels o, q, s and u**) was variable, with observed high RBFOX2 staining in early neoplastic lesions present within the core (**panel s**). The box plots define the 25th and 75th percentiles (box), median (solid line) and the minima and maxima (whiskers). Data in panel b are presented as mean values +/- SD. Scale bar is 100um. Panels c-v are 20X scanned images.

Supplemental Figure 4.

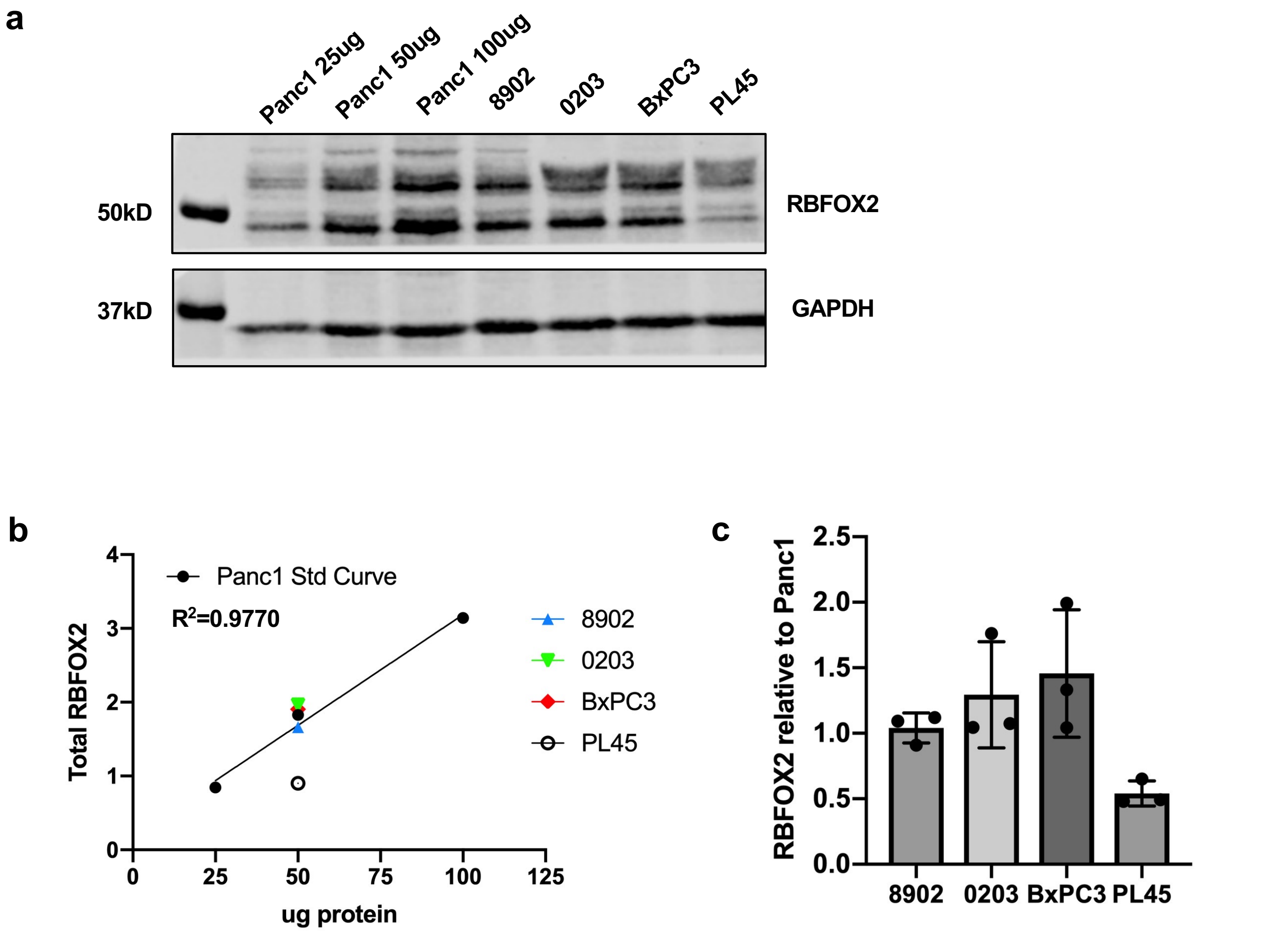

Supplemental Figure 4. Quantitation of total RBFOX2. Western blot analysis of total RBFOX2 in 50ug whole cell lysates from epithelial-like cell lines relative to a Panc1 standard curve (**a**). Panc1 standard curve for total RBFOX2 and sample cell lines (**b**). Quantitation of RBFOX2 relative to Panc1 representative of 3 independent experiments (**c**). Data in panels a and b are representative of three independent experiments generated across three cell passages. Data in panel c are presented as the mean values +/- SD.

Supplemental Figure 5.

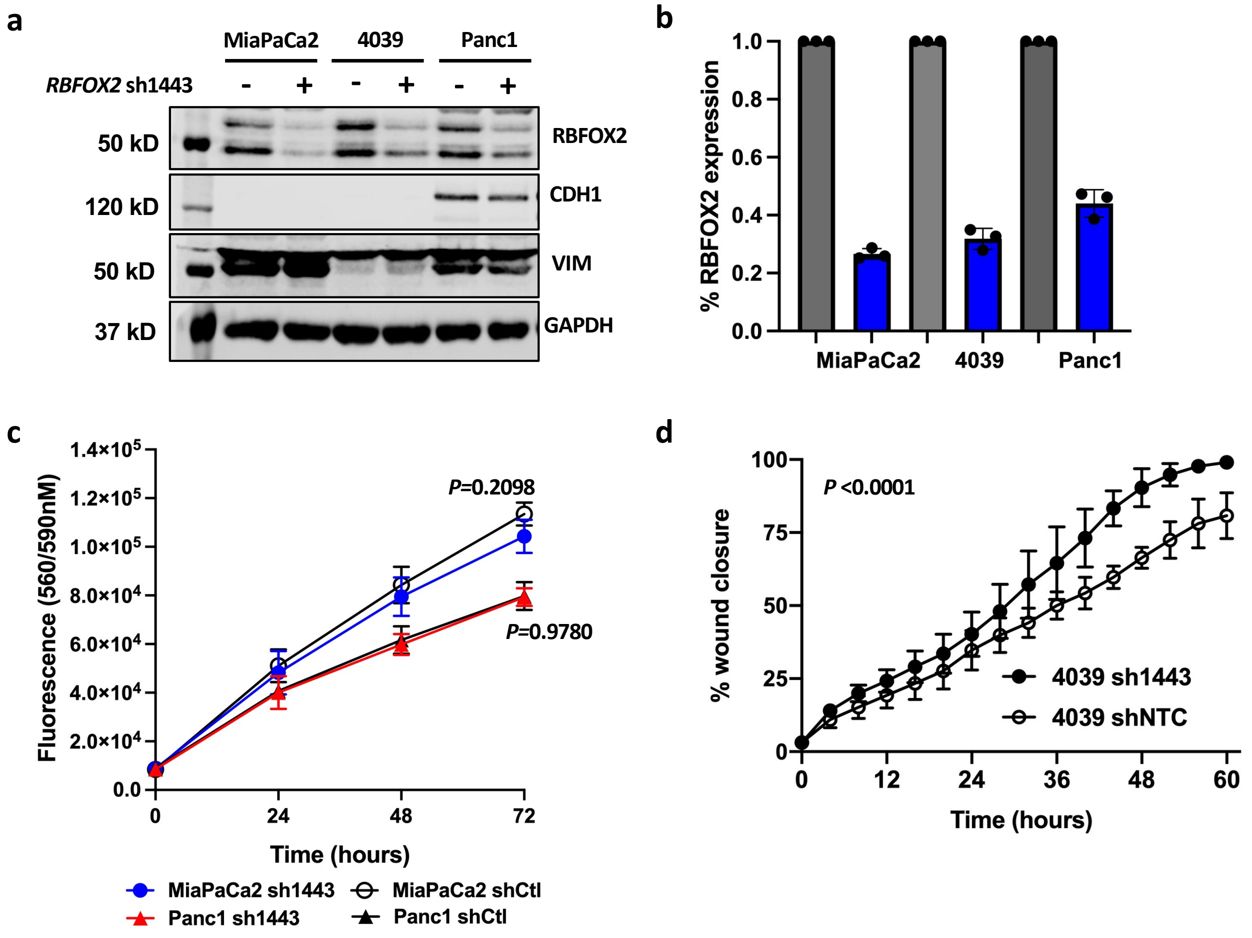

Supplemental Figure 5. Inducible *RBFOX2* knockdown recapitulates cellular phenotypes. A DOX-inducible shRNA targeting *RBFOX2* decreased RBFOX2 protein expression (**a**) in the absence of changes in mesenchymal-like properties of the cells as assessed by expression of CDH1 (e-cadherin) and vimentin (VIM). Quantification of RBFOX2 protein reduction for cells with inducible *RBFOX2* knockdown compared to inducible non-targeting shRNA control was determined from an average of 3 westerns (**b**). Inducible *RBFOX2* depletion does not change cellular growth under adherent conditions (**c**) for MiaPaCa2 cells (P=0.2098; DF=3; F=1.601) or for Panc1 cells (P=0.9780; DF=3; F=0.065). 4039 cells show significantly increased cellular migration with inducible RBFOX2 depletion (**d**, 2-factor ANOVA P<0.0001; DF=15; F=9.169). Cells were treated with doxycycline for 5 days prior to performing cellular-based assays. Data in panels a and b are representative of three independent experiments generated across multiple cell passages. Data in panel b are presented as the mean values +/- SD. Data in panels c and d are from individual representative experiments, with 6-8 technical replicates plated per line per experiment. Three independent experiments were performed across three cell passages. Statistical analysis was performed using PRISM and the P-values reported for the indicated statistical tests are shown.

Supplemental Figure 6.

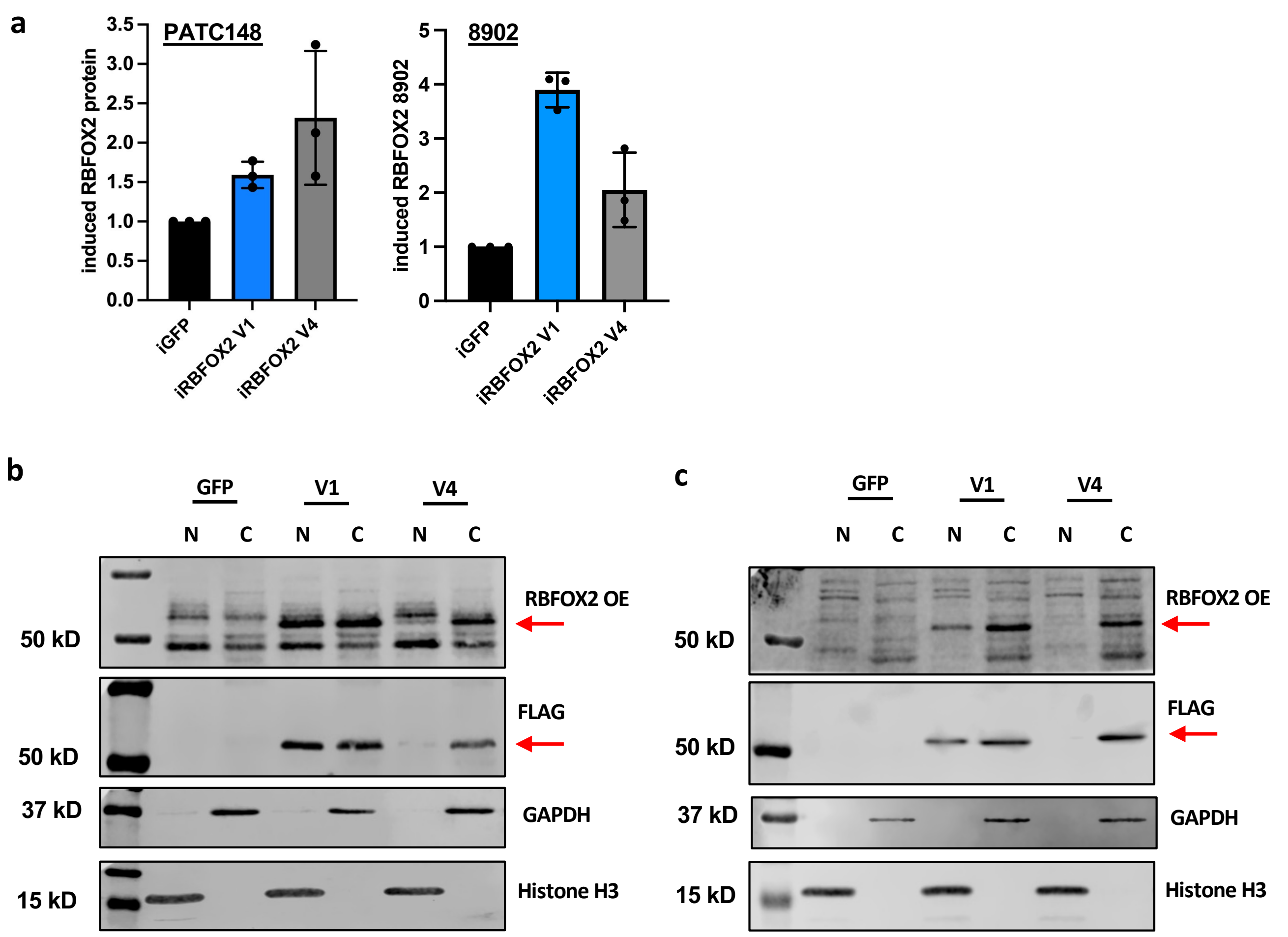

Supplemental Figure 6. Quantification and cellular localization of RBFOX2 isoforms. Relative quantification of induced RBFOX2 V1 and V4 isoforms from whole cell lysates of PATC148 and 8902 cells compared to GFP control shows similar expression levels of the different isoforms in each isogenic series (**a**). Cell fractionation of PATC148 cells (**b**) and 8902 cells (**c**) with an induced GFP control vector ("GFP") confirms that endogenous RBFOX2 is expressed in both nuclear ("N") and cytoplasmic ("C") fractions. Induced over-expression of FLAG-tagged RBFOX2 V1 protein is detected in both the nuclear ("N") and cytoplasmic ("C") fractions marked by the expression of the FLAG tag in both fractions (red arrows). Induced overexpression of the V4 isoform is detected only in the cytoplasmic fraction. Western blot detection of GAPDH only in the cytoplasmic ("C") fraction and Histone H3 only in the nuclear ("N") fraction confirms clean cellular fractionation. Data in panel a are presented as mean values +/- SD. Data in panels b and c are from individual experiments and are representative of the data generated from three independent experiments across multiple cell passages.

Supplemental Figure 7.

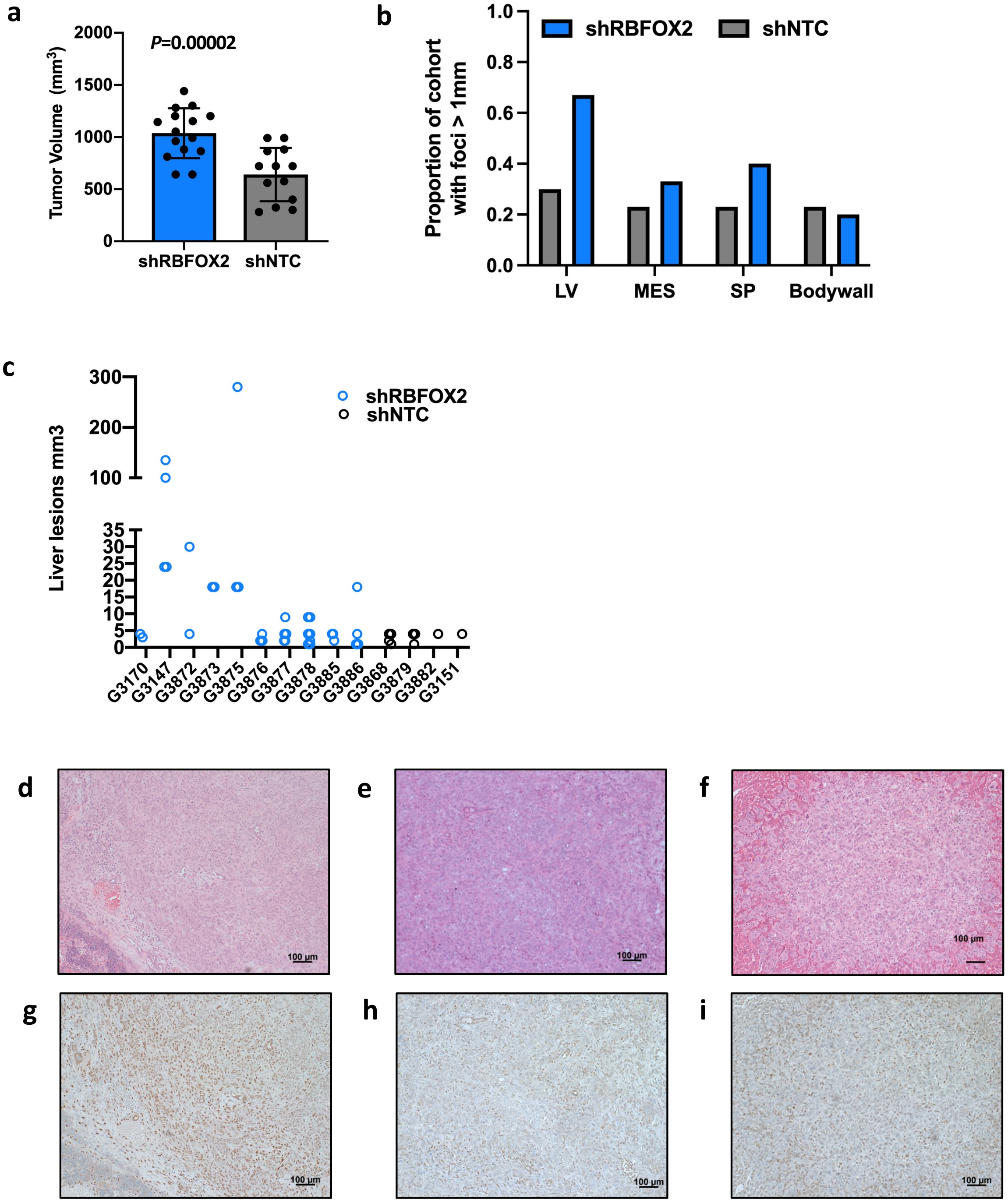

Supplemental Figure 7. RBFOX2 depletion in Panc1 cells promotes PDAC metastasis. Pancreas tumor volumes were significantly increased in animals with RBFOX2-depleted cells (shRBFOX2, n=15) compared to animals injected with RBFOX2-replete cells (shNTC, n=13) (**a**, *P*=0.0002, unpaired t-test; t=4.241, df=26). Data are combined from three independent experiments with randomly assigned male and female mice. RBFOX2 depletion increased the incidence of macro metastasis to the liver (LV) and spleen (SP) compared to Panc1 cells replete for RBFOX2, represented as the proportion of the cohort with at least one focus in the target organ measured as greater than 1 mm at necropsy (**b**), with similar spread to the body wall adjacent to the pancreas for both models. The volume of metastatic lesions (mm^3) collected at necropsy is graphed on a per-animal basis (**c**). Four mice from the control cohort exhibited liver lesions of comparable size (less than 5 mm^3) while the Panc1 RBFOX2 depleted cohort exhibited a range of sizes and density of liver lesions, with only those that could be independently measured shown. H&E staining of a pancreas tumor from Panc1 cells expressing the non-targeting shRNA (**d**) shows tumor with surrounding normal pancreas (lower left corner). H&E staining of a pancreas tumor (**e**) and liver metastasis (**f**) from Panc1 cells with shRNA mediated *RBFOX2* knockdown. Analysis of RBFOX2 expression by immunocytochemistry demonstrates robust RBFOX2 expression in tumors from replete cells (**g**) and decreased signal in RBFOX2 depleted pancreas tumor (**h**) and resulting liver metastasis (**i**). Surrounding normal liver expresses low RBFOX2 protein. Scale bar is 100um. Data in panel a are presented as mean values +/- SD.

Supplemental Figure 8.

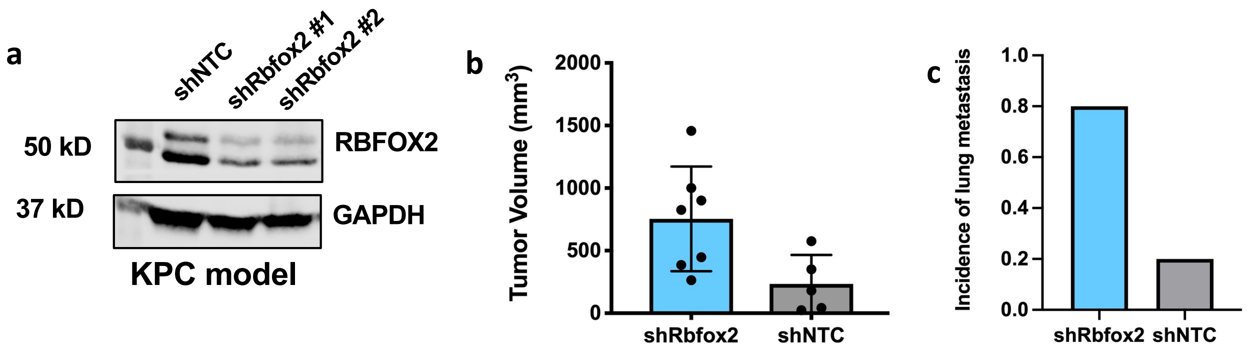

Supplemental Figure 8. RBFOX2 depletion in murine KPC cells significantly increases PDAC metastasis to the lungs in a tail vein model. *Rbfox2* knockdown in a murine cell line derived from the KPC PDAC GEMM (**a**) promotes PDAC growth in a C57Bl/6J immunocompetent orthotopic mouse model (**b**, n=7, sh*Rbfox2* and n=5, shNTC), where only half of the mice with the control vector developed tumors. Data are representative of two independent experiments. RBFOX2-depleted KPC cells demonstrate enhanced colonization in the lung in a tail vein injection model (**c,** n=5, sh*Rbfox2* and n=5, shNTC)**.** Data are presented as mean values +/- SD.

Supplemental Figure 9.

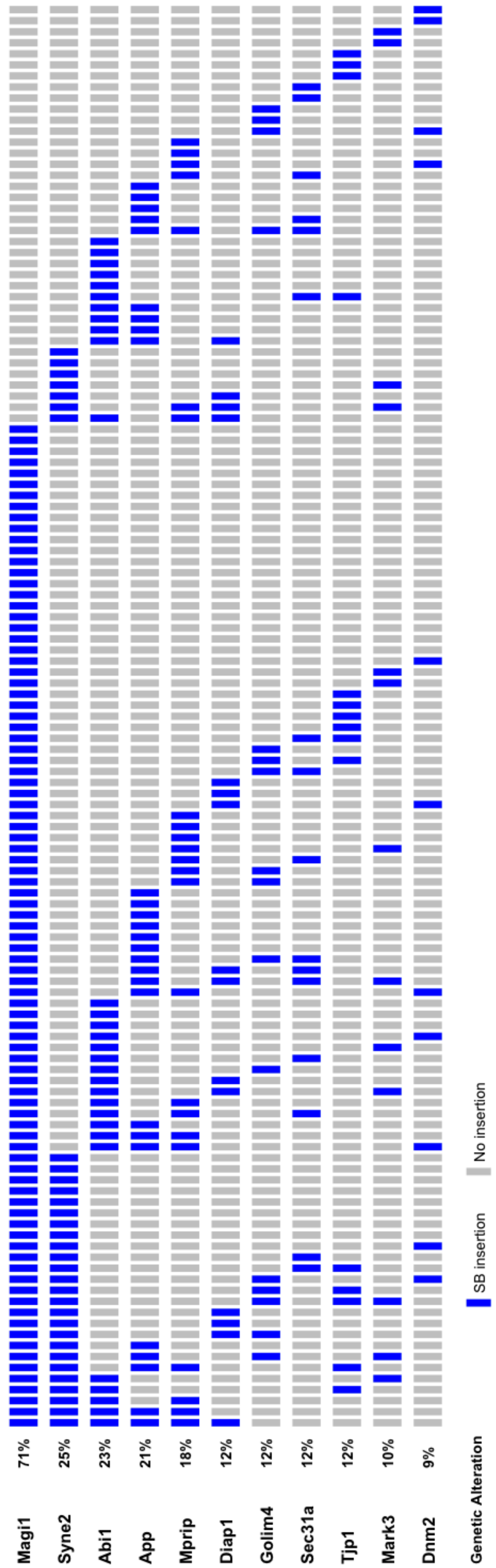

Supplemental Figure 9. RBFOX2 regulates alternative splicing of transcripts encoded by mouse orthologs identified from Sleeping Beauty PDAC mouse model. Eleven exon splicing events controlled by RBFOX2 occur in transcripts encoded by statistically defined cancer genes in a Sleeping Beauty forward genetic screen in a PDAC mouse model [1, 2]. An oncoprint highlights the incidence of SB transposon insertions in these mouse orthologs, with "hits" depicted by blue bars and the percentage of tumors with insertions in a population of 172 tumors highlighted on the left-hand side.

Supplemental Figure 10.

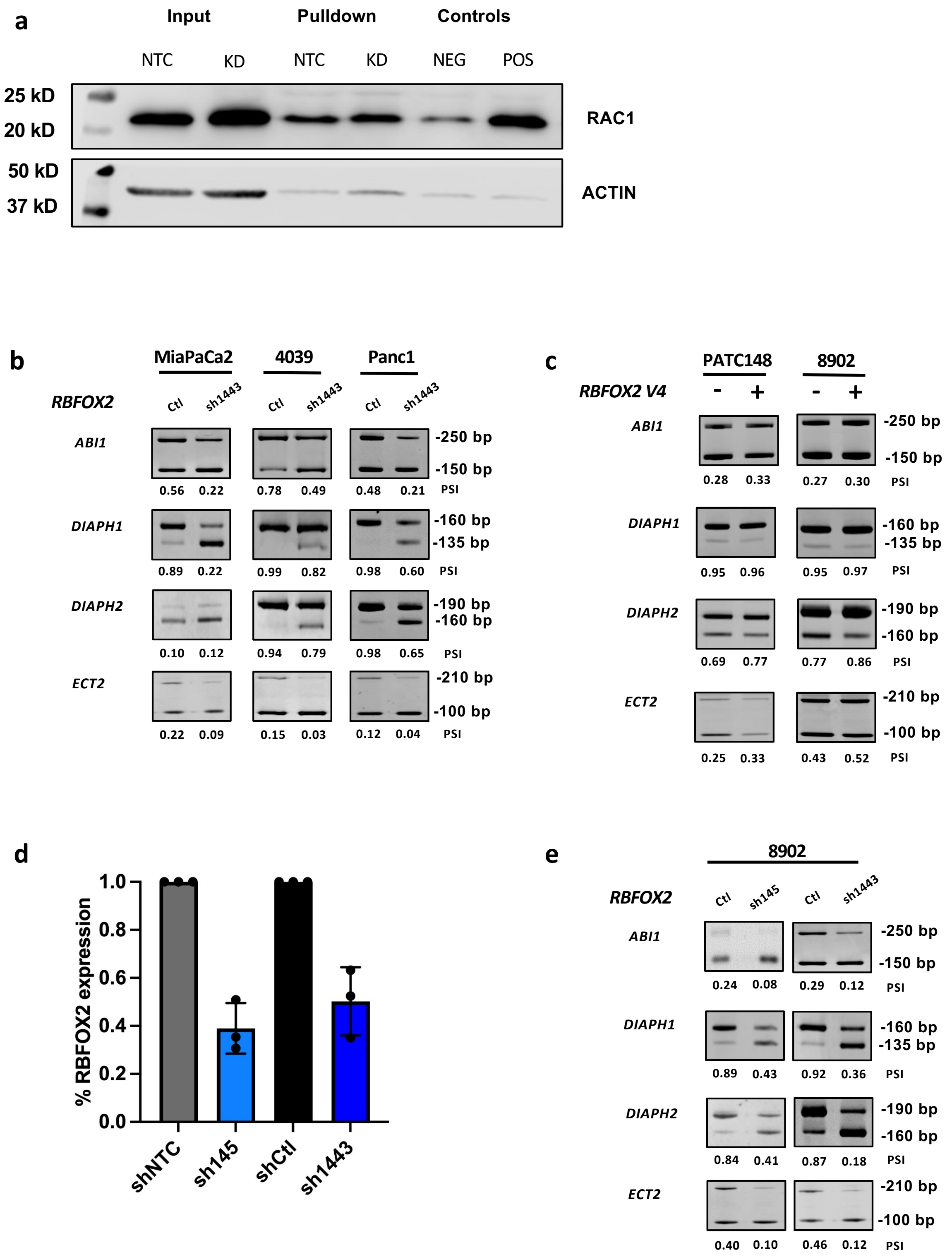

f

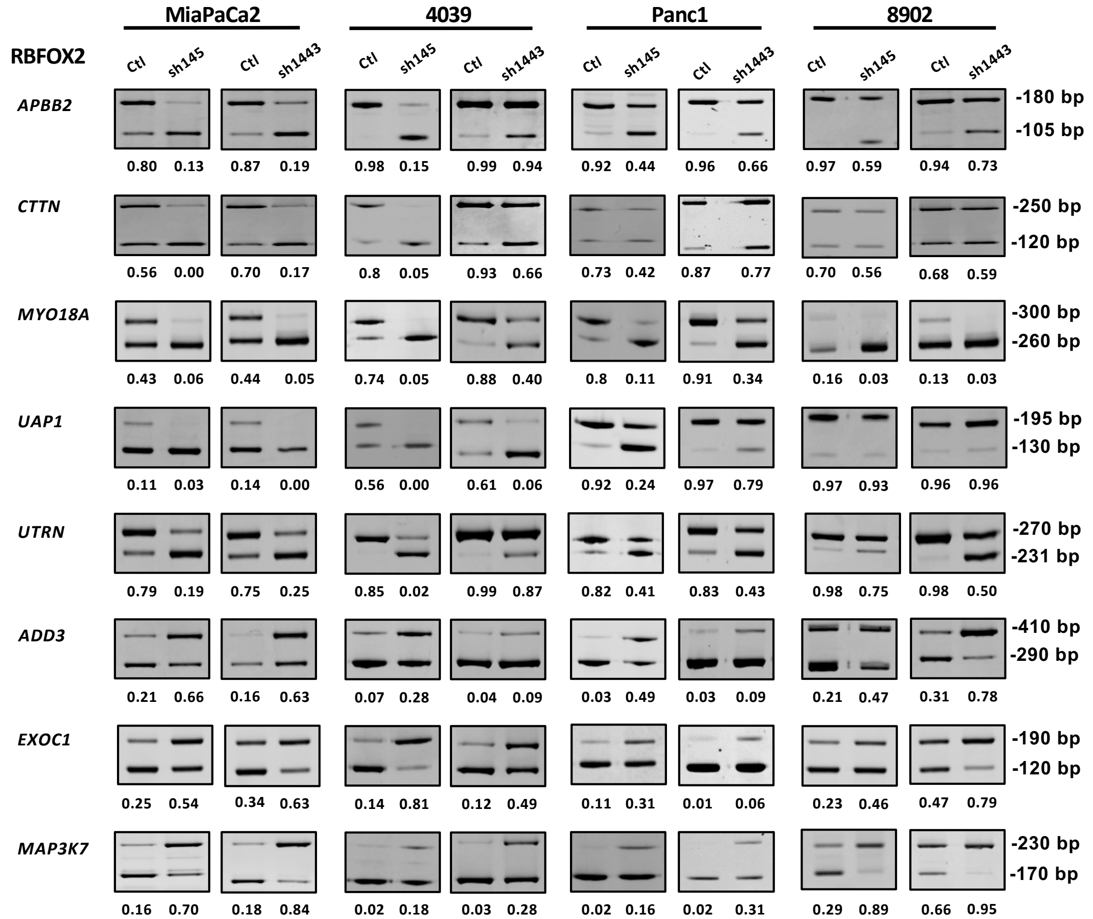

Supplemental Figure 10. RBFOX2 deregulation promotes alternative splicing of RBFOX2 target exons. Levels of GTP-bound RAC1 in 4039 cells replete and depleted for RBFOX2 were unchanged using a GTP pulldown assay (**a**). Data are representative of three independent experiments generated across three cell passages. Real-time PCR (RT-PCR) validation of RBFOX2 target exon usage in PDAC cells with inducible knockdown of RBFOX2 (sh1443) confirms exon skipping of RBFOX2 target exons in *ABI1*, *DIAPH1*, *DIAPH2* and *ECT2* compared to cells with an inducible nontargeting shRNA (Ctl). Percent spliced-in (PSI) values are calculated for RBFOX2 target exons in each isogenic pair (**b**). Induced *RBFOX2* V4 isoform expression in PATC148 and 8902 cells does not change the PSI of spliced target exons in *ABI1*, *DIAPH1*, *DIAPH2* and *ECT2* transcripts compared to control cells (**c**). The percent expression of RBFOX2 protein upon constitutive or induced RBFOX2 knockdown in 8902 cells is shown (**d**). PSI values for RBFOX2 target exons in *ABI1*, *DIAPH1*, *DIAPH2* and *ECT2* decreased in 8902 cells with constitutive (sh145) or inducible (sh1443) RBFOX2 knockdown in 8902 cells compared to controls (**e**). Expanded analysis of RBFOX2 target exon splicing in PDAC by using either the constitutive shRNA targeting RBFOX2 (sh145) and the constitutive non-targeting shRNA control (ctl) or the inducible sh1443 targeting RBFOX2 and the matched inducible non-targeting control (ctl) shows parity for splicing shifts based on PSI values for each isogenic cell line pair (**f**). The degree of exon skipping with RBFOX2 knockdown corresponds with the increased abundance of the lower PCR product and reduced PSI value and is dependent on the degree of RBFOX2 knockdown. The majority of RBFOX2 target exons are skipped in the absence of RBFOX2, while for select transcripts *ADD3*, *EXOC1* and *MAP3K7*, RBFOX2 target exons are included upon reduced RBFOX2 expression. Splicing assays are representative of three independent RT-PCR assays with PSI quantitation across multiple cell passages. Data in panel d are presented as mean values +/- SD.

Supplemental Figure 11.

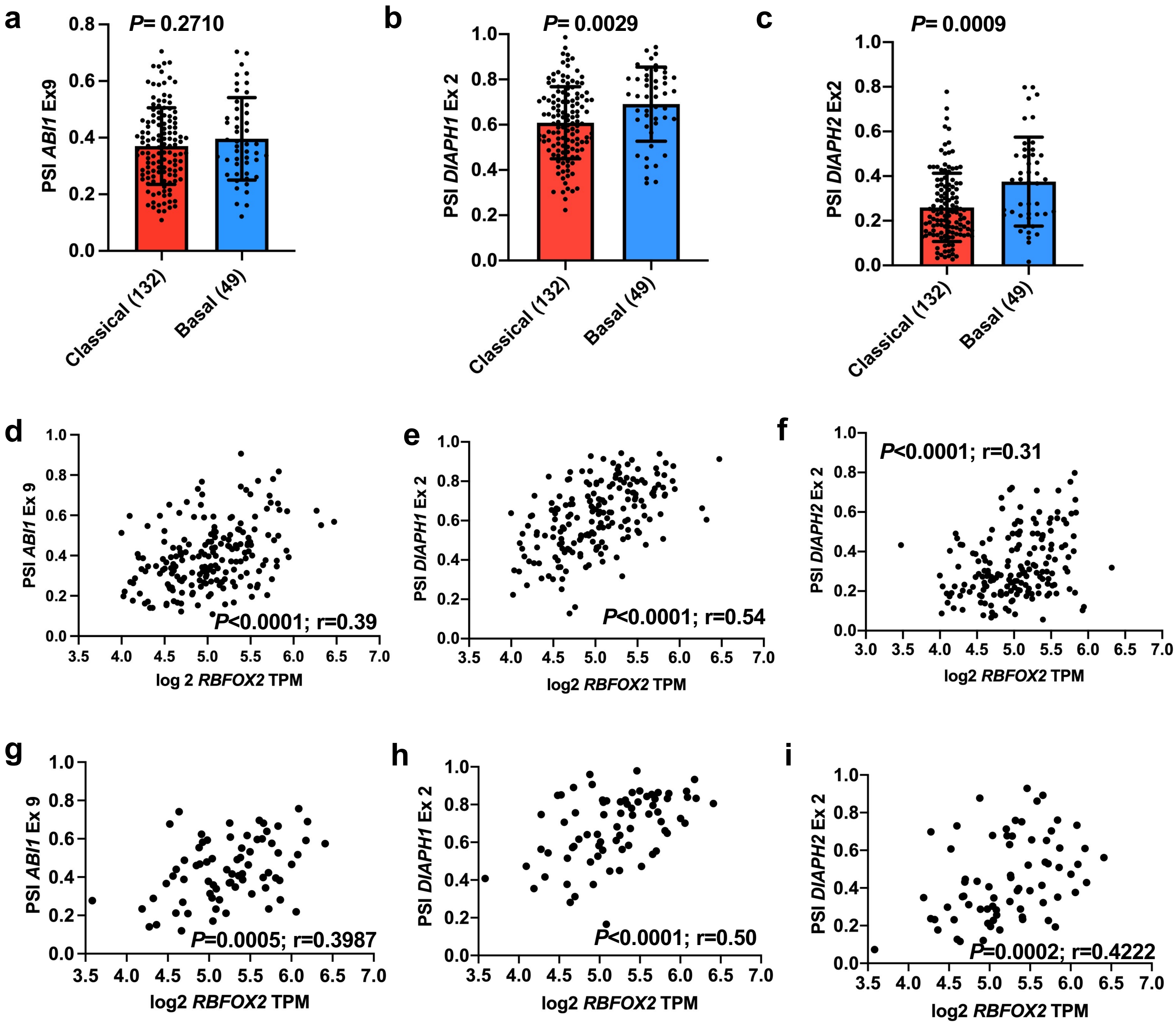

Supplemental Figure 11. RBFOX2 target exon PSI variance in PDAC subtypes. PSI analysis in RNA-seq data set EGAD00001004548 [5-8] shows the mean and population variance in classical (n=132) and basal (n=49) PDAC subtypes for RBFOX2 target exons *ABI1* exon 9 (**a** *P*=0.2710, t=1.104, df=173), *DIAPH1* exon 2 (**b**, *P*=0.0029, t=3.015, df=175) and *DIAPH2* exon 2 (**c**, *P*=0.0009, t=3.495, df=61.89, Welch's t-test). Individual correlation plots for target exon PSI values positively correlate with *RBFOX2* gene expression (log2 TPM) in PDAC tumors using Pearson's statistical correlation for *ABI1* exon 9 (n=191 XY pairs) (**d**, *P*<0.0001; r=0.39, 95% confidence interval 0.2555 to 0.4984), *DIAPH1* exon 2 (n=190 XY pairs) (**e**, *P*<0.0001; r=0.54, 95% confidence interval 0.4389 to 0.6396), and *DIAPH2* exon 2 (n=173 XY pairs) (**f**, *P*<0.0001; r=0.31, 95% confidence interval 0.1684 to 0.4387) and in liver metastases for *ABI1* exon 9 (n=73 XY pairs) (**g**, *P*=0.0005; r=0.3987, 95% confidence interval 0.01857 to 0.5759), *DIAPH1* exon 2 (n=75 XY pairs) (**h**, *P*<0.0001; r=0.50, 95% confidence interval 0.3045 to 0.6507), and *DIAPH2* exon 2 (n=72 XY pairs) (**i**, *P*=0.0002; r=0.4222, 95% confidence interval 0.2112 to 0.5956). Data are presented as mean values +/- SD.

Supplemental Figure 12.

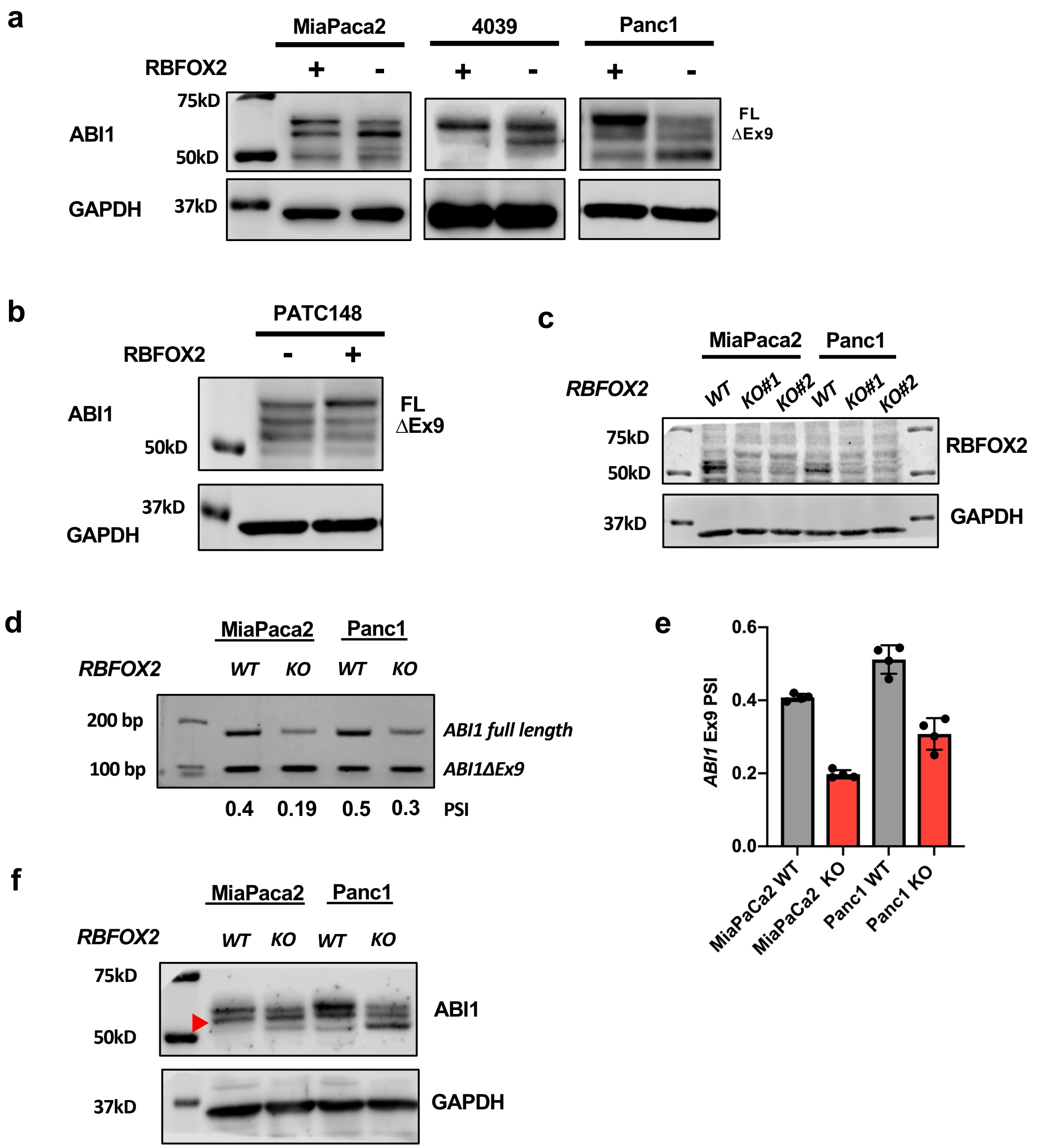

Supplemental Figure 12. **RBFOX2 knockout modulates ABI1 isoforms.** ShRNA-mediated RBFOX2 depletion promotes a shift in ABI1 protein isoform abundance towards the ΔEx9 isoform (**a**). RBFOX2 repletion in PATC148 cells promotes a shift towards increased abundance of the ABI1 full-length protein isoform (**b**). RBFOX2 CRISPR depletion confirmed by western in both MiaPaCa2 and Panc1 cells (**c**) promotes the *ABI1* splice shifts (**d**) we observed with RBFOX2 knockdown via shRNA. Quantitation of *ABI1* splicing shifts with RBFOX2 KO (**e**) shows a 50% reduction in *ABI1* full length mRNAs. Western blot analysis of ABI1 protein isoforms shows increased abundance of the ΔEx9 isoform (**f, red arrowhead**) with CRISPR-mediated RBFOX2 knock-out. Gels are representative of four independent assays from multiple cell line passages. Data in panel e are presented as mean values +/- SD.

Supplemental Figure 13.

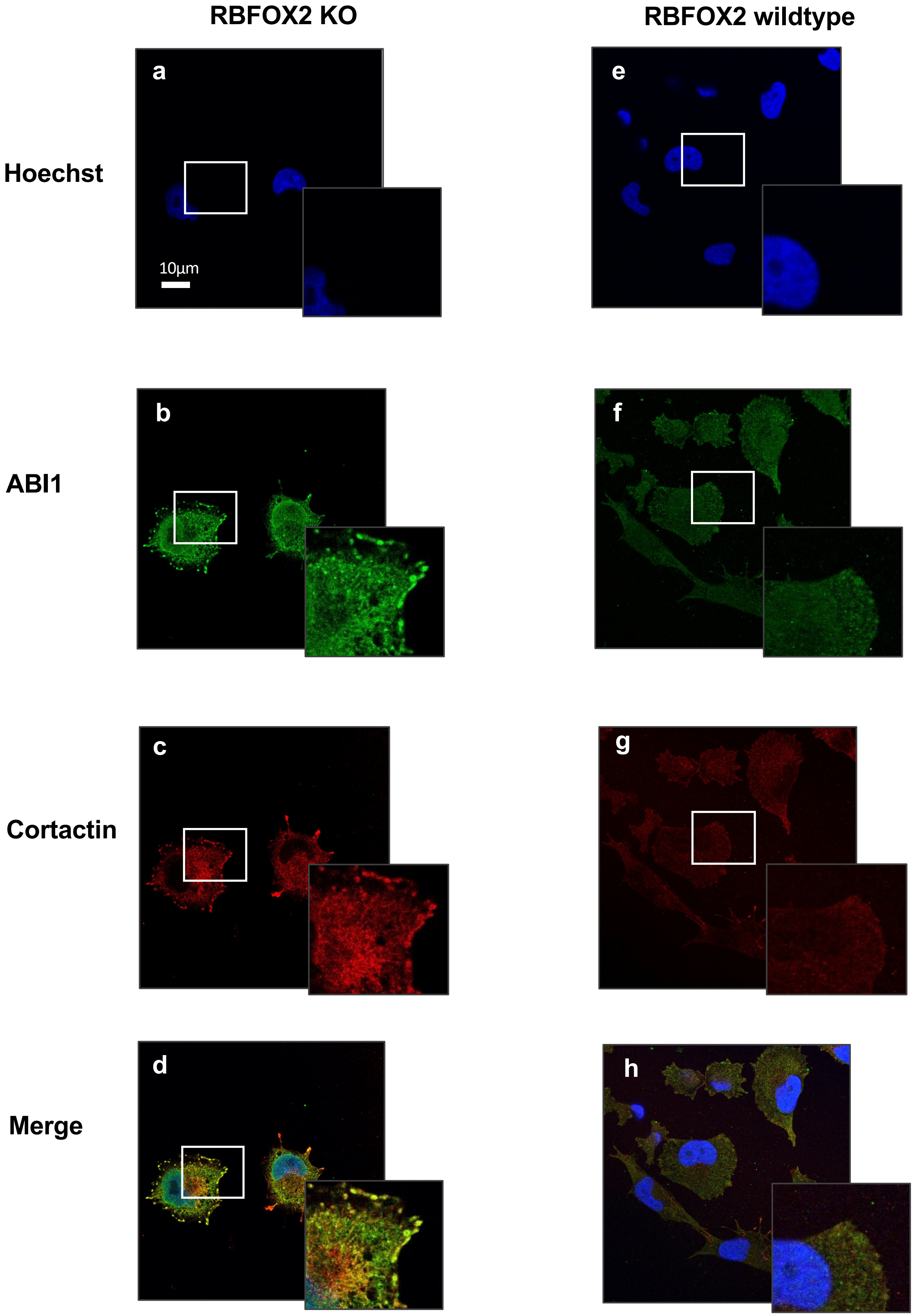

Supplemental Figure 13. RBFOX2 loss promotes ABI1 colocalization with Cortactin at the cell periphery in PDAC cells. MiaPaCa2 cells with CRISPR-mediated RBFOX2 depletion (**a-d**) exhibit robust ABI1 staining and redistribution (**b**) to the cell periphery indicated by Cortactin staining (**c**). The merged image shows ABI1 and Cortactin signals overlap (**d**, see inset). In contrast, RBFOX2-replete cells (**e-h**) exhibit a uniform distribution of ABI1 (**f**) and Cortactin (**g**) throughout the cell. The merged image (**h** and inset) shows less overlap of ABI1 and Cortactin at the cell periphery. Nuclei were stained using Hoechst 33342 (**a, e**). Scale bar is 10um.

Supplemental Table 1

Gene	Assay	Forward Primer Sequence<br>5' to 3'	Reverse Primer Sequence<br>5' to 3'	Reference
ABI1	splicing	CAATGACCAGGCAGATATCTCG	GCGGTGGAGTTGGACTATCA	9
ADD3	splicing	CAGGACCACAATCTCAGTTGC	TCGCTTAGCAAGCTCATCTTC	9
APBB2	splicing	TTCTGTAACGCCATCTCCCACC	TGCATAGCGTAGGGTTGCTCC	9
CTTN	splicing	ACAGACAAGACAAATGTGCCC	TATCCATCCGATCCTTCTGC	9
DIAPH1	splicing	GACAAGAAGAAGGGCCGGAGC	TGAGCAGAATTGGGCTTTTCC	10
DIAPH2	splicing	CCGCCAATGAAGAGGAAACGAA	TGCAGGAAACTCACTCATCGC	9
ECT2	splicing	GTGATATTGGTTCAAGAAGCTGG	CAAATTCTTCCACTGACTCCATC	9
EXOC1	splicing	GACTGGCACAACTAAAGAAAGCA	TCCAACAGGGAAGATGACTGAGA	9
MAP3K7	splicing	TGGGAGCAGTGTGGAGAGCTTG	CTGACCAGGTTCTGTTCCAGTTAC	10
MYO18A	splicing	ATTCAAGCGCATCGGGGAC	TCGTGCTCATCATCTGACCG	9
RBFOX2	splicing	CCCTTTAGTTCCTGGCTTCC	CTGTGCATATCTGTAGGCTG	lab-designed
UAP1	splicing	TCATTGCTGGGTCCTCAATGC	CTCCAGCATAGGAGATAAGAG	10
UTRN	splicing	CAAACACCCTCGACTTGGTT	TGGCAATACTGCTGGATGAG	9
RBFOX2	hairpin	TGCTGTTGACAGTGAGCGATGGCTGTAATTTTCTATGTTATAGTGAAGCCA<br>CAGATGTATAACATAGAAAATTACAGCCAGTGCCTACTGCCTCGGA		11
RBFOX2	sgRNA	GTGGGAATTCCATTCTGCGGNGG		lab-designed

Supplementary References

1. Mann, K.M., et al., *Sleeping Beauty mutagenesis reveals cooperating mutations and pathways in pancreatic adenocarcinoma.* Proceedings of the National Academy of Sciences of the United States of America, 2012. **109**(16): p. 5934-41.
2. Newberg, J.Y., et al., *SBCDDB: Sleeping Beauty Cancer Driver Database for gene discovery in mouse models of human cancers.* Nucleic Acids Res, 2018. **46**(D1): p. D1011-D1017.
3. Zhang, G., et al., *DPEP1 inhibits tumor cell invasiveness, enhances chemosensitivity and predicts clinical outcome in pancreatic ductal adenocarcinoma.* PLoS One, 2012. **7**(2): p. e31507.
4. Cao, L., et al., *Proteogenomic characterization of pancreatic ductal adenocarcinoma.* Cell, 2021. **184**(19): p. 5031-5052 e26.
5. Aung, K.L., et al., *Genomics-Driven Precision Medicine for Advanced Pancreatic Cancer: Early Results from the COMPASS Trial.* Clin Cancer Res, 2018. **24**(6): p. 1344-1354.
6. Connor, A.A., et al., *Integration of Genomic and Transcriptional Features in Pancreatic Cancer Reveals Increased Cell Cycle Progression in Metastases.* Cancer Cell, 2019. **35**(2): p. 267-282 e7.
7. Connor, A.A., et al., *Association of Distinct Mutational Signatures With Correlates of Increased Immune Activity in Pancreatic Ductal Adenocarcinoma.* JAMA Oncol, 2017. **3**(6): p. 774-783.
8. Notta, F., et al., *A renewed model of pancreatic cancer evolution based on genomic rearrangement patterns.* Nature, 2016. **538**(7625): p. 378-382.
9. Venables, J.P., et al., *Cancer-associated regulation of alternative splicing.* Nat Struct Mol Biol, 2009. **16**(6): p. 670-6.
10. Zhang, C., et al., *Defining the regulatory network of the tissue-specific splicing factors Fox-1 and Fox-2.* Genes Dev, 2008. **22**(18): p. 2550-63.
11. Pelossof, R., et al., *Prediction of potent shRNAs with a sequential classification algorithm.* Nat Biotechnol, 2017. **35**(4): p. 350-353.

Supplemental Figure 2 Panel c

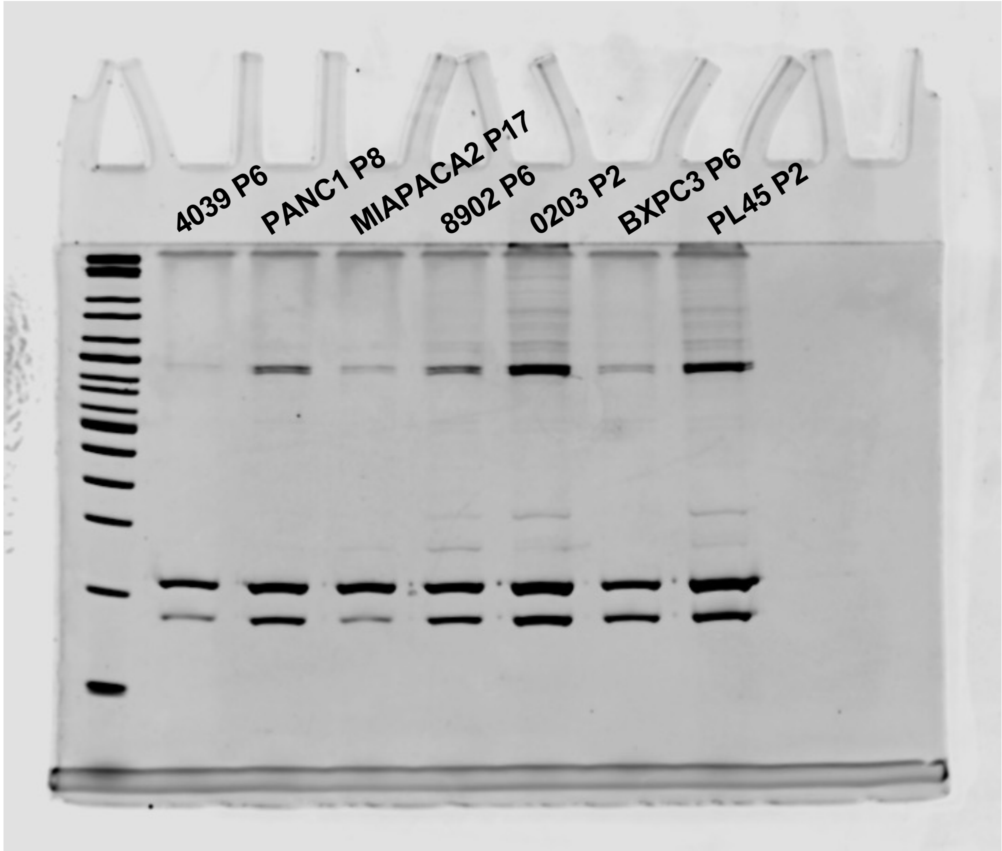

Supplemental Figure 5 panel a

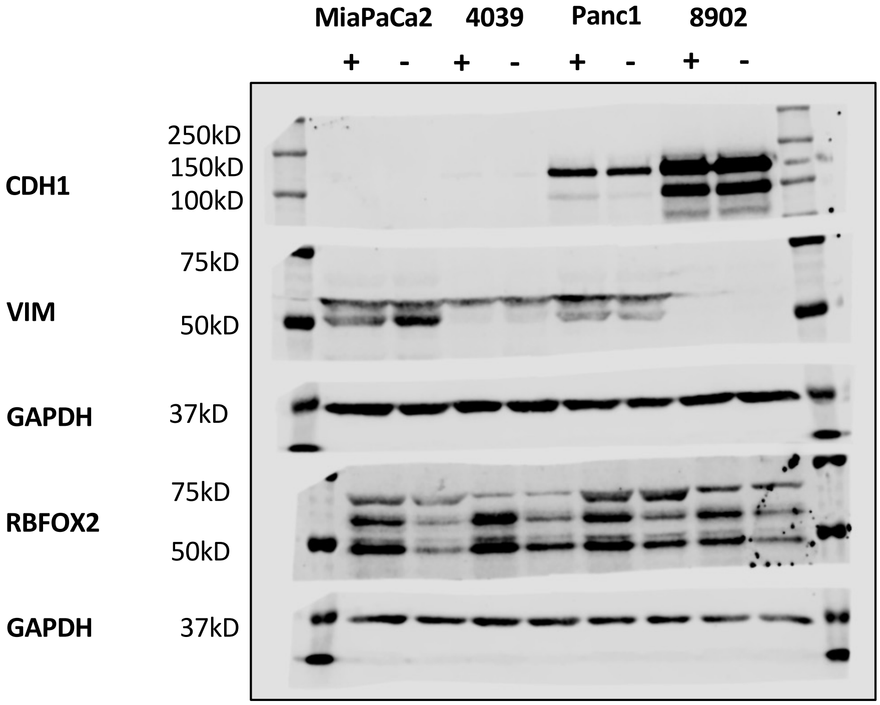

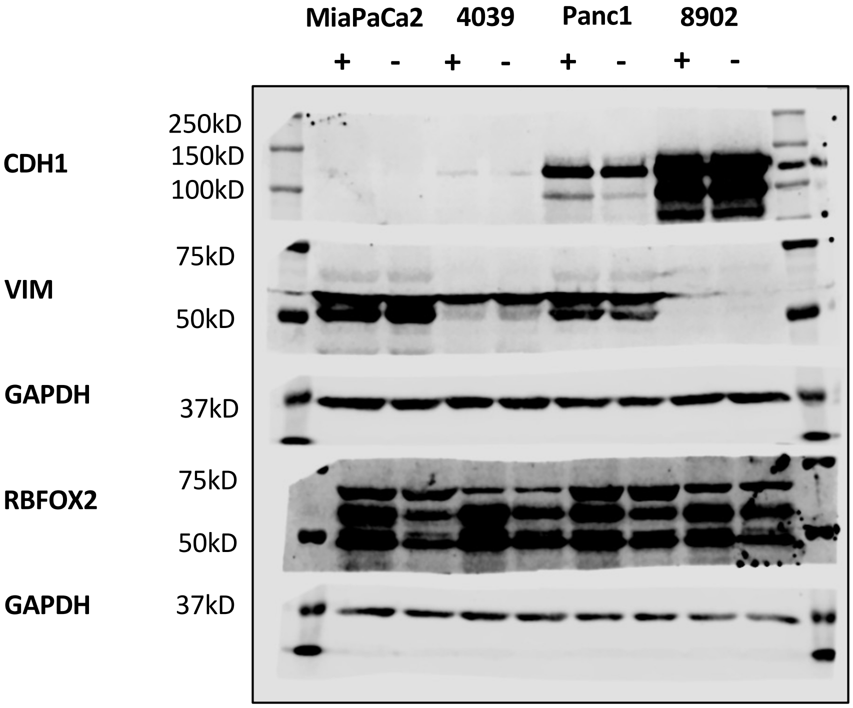

Darker exposure for VIM

Supplemental Figure 10

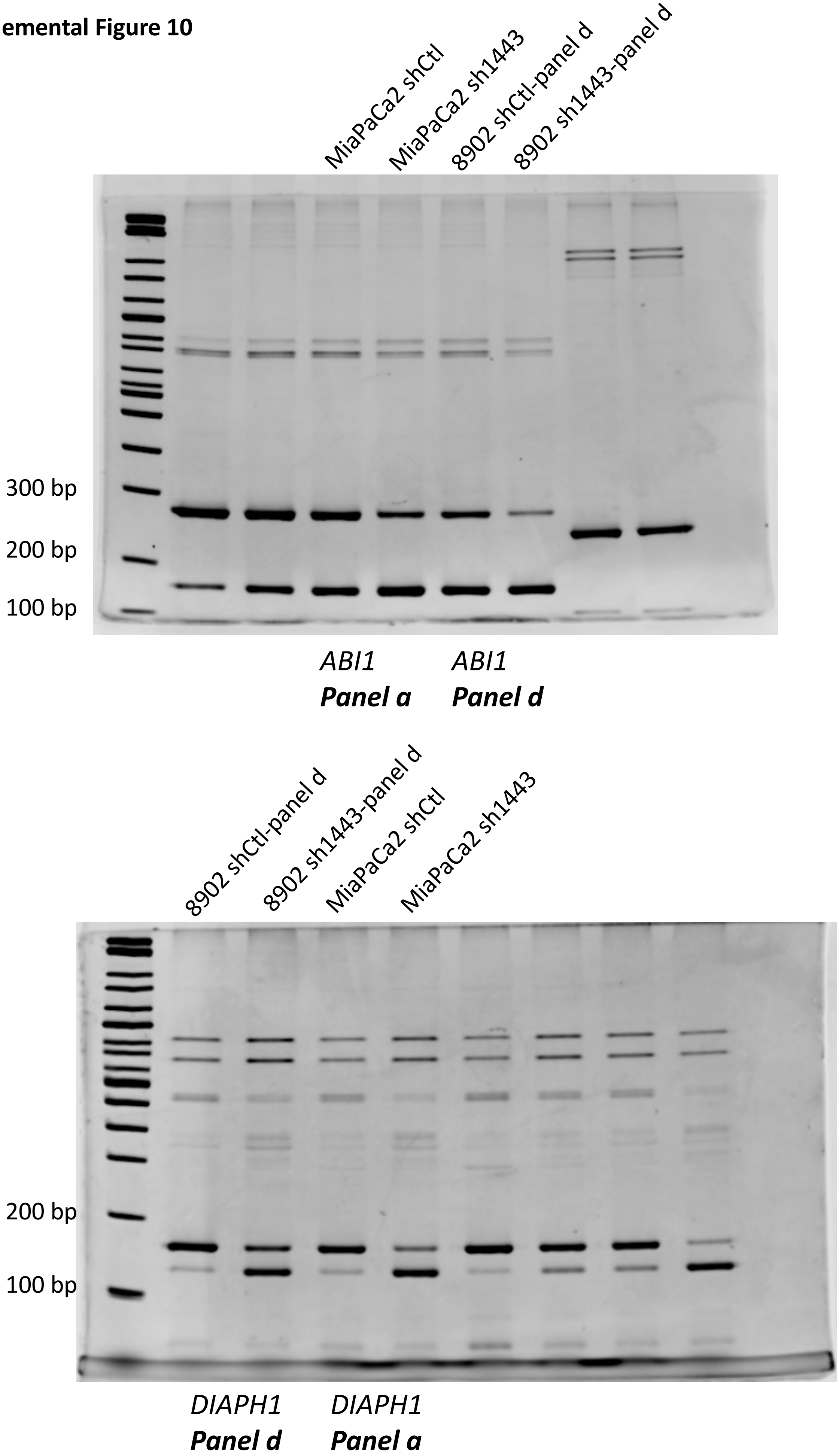

Supplemental Figure 10

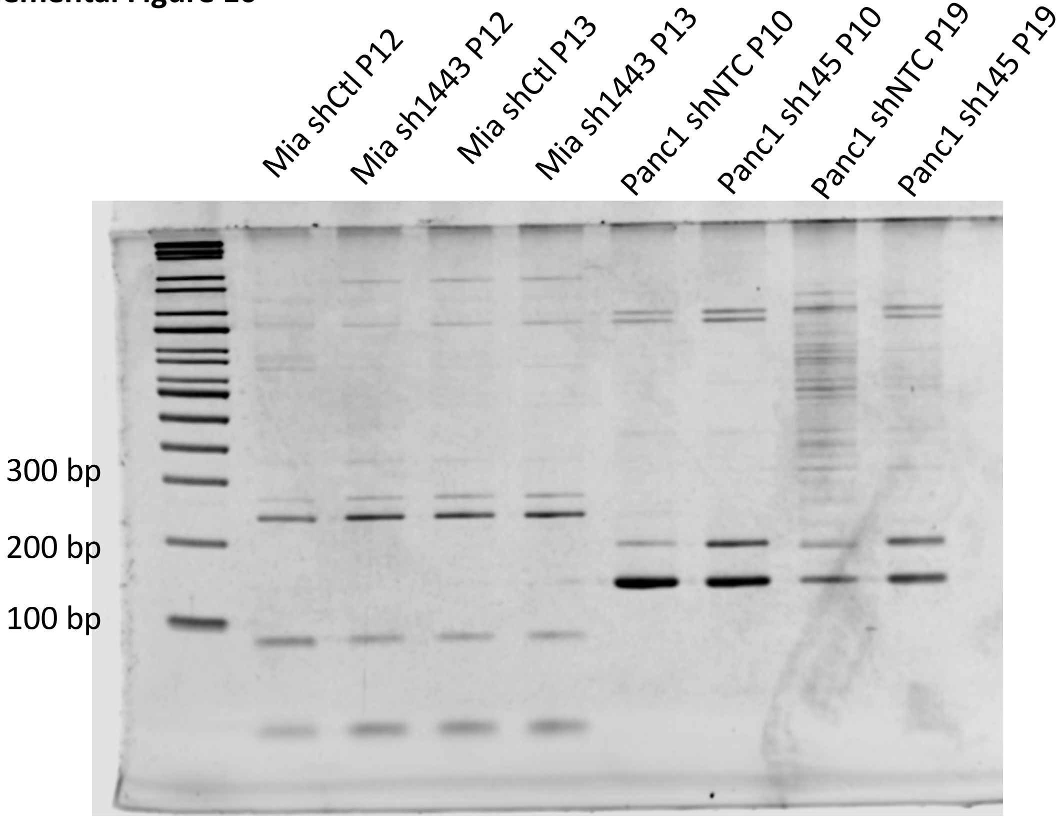

DIAPH2 Panel a

EXOC1 Panel e

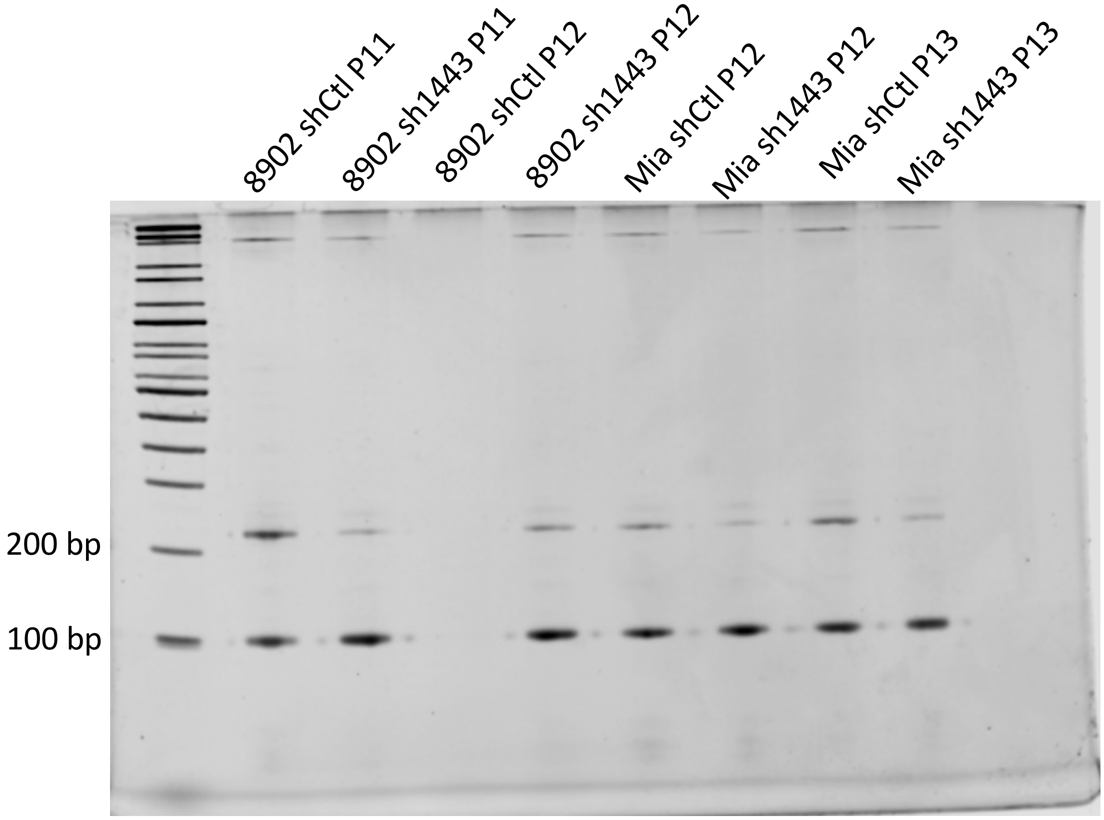

ECT2 Panel d

ECT2 Panel a

Supplemental Figure 10

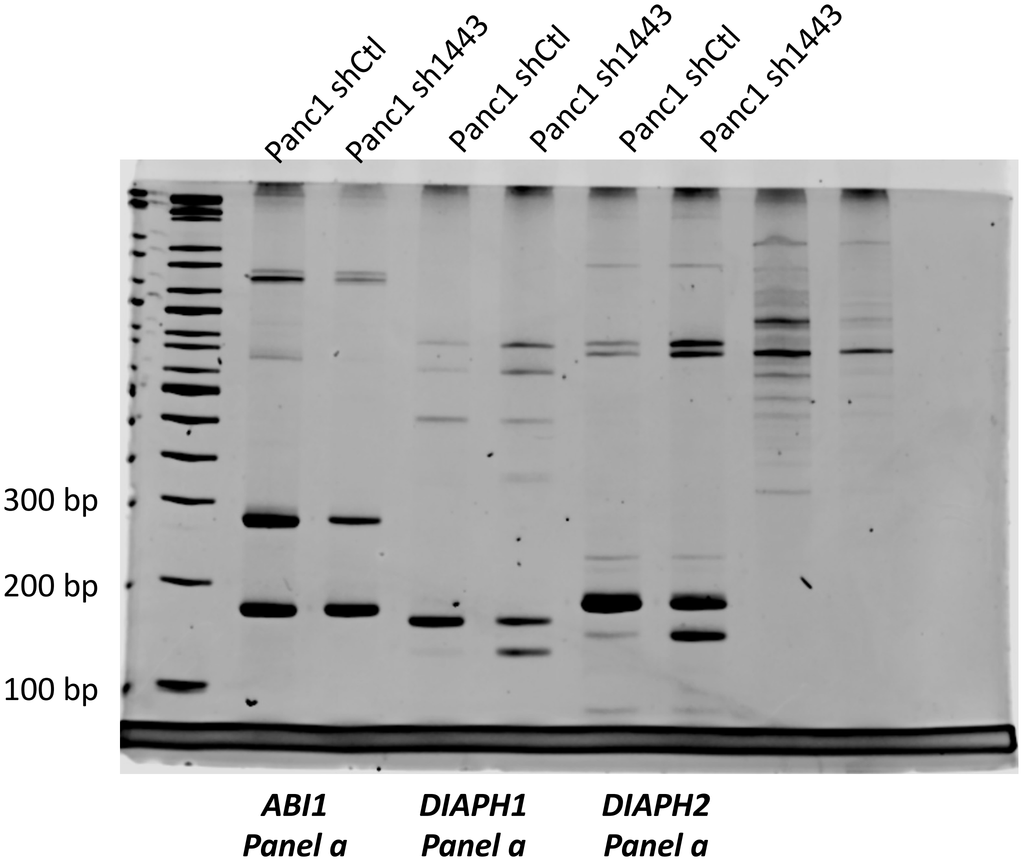

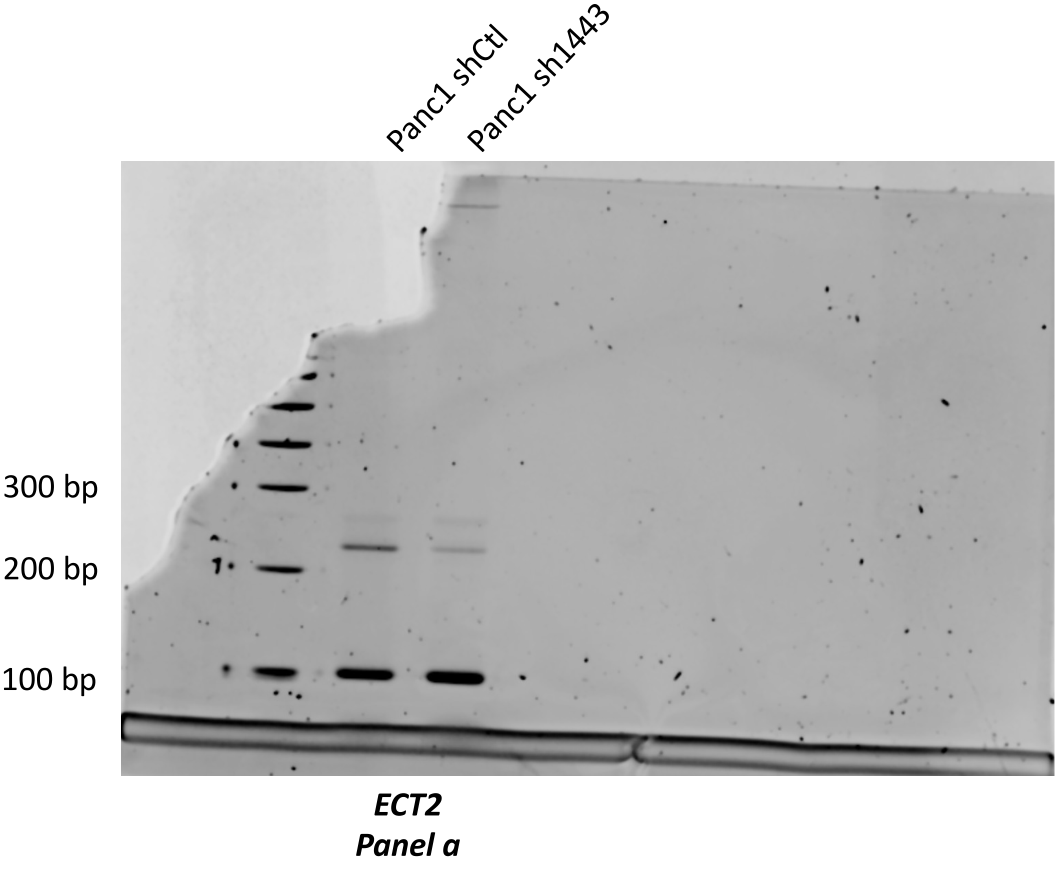

Supplemental Figure 10

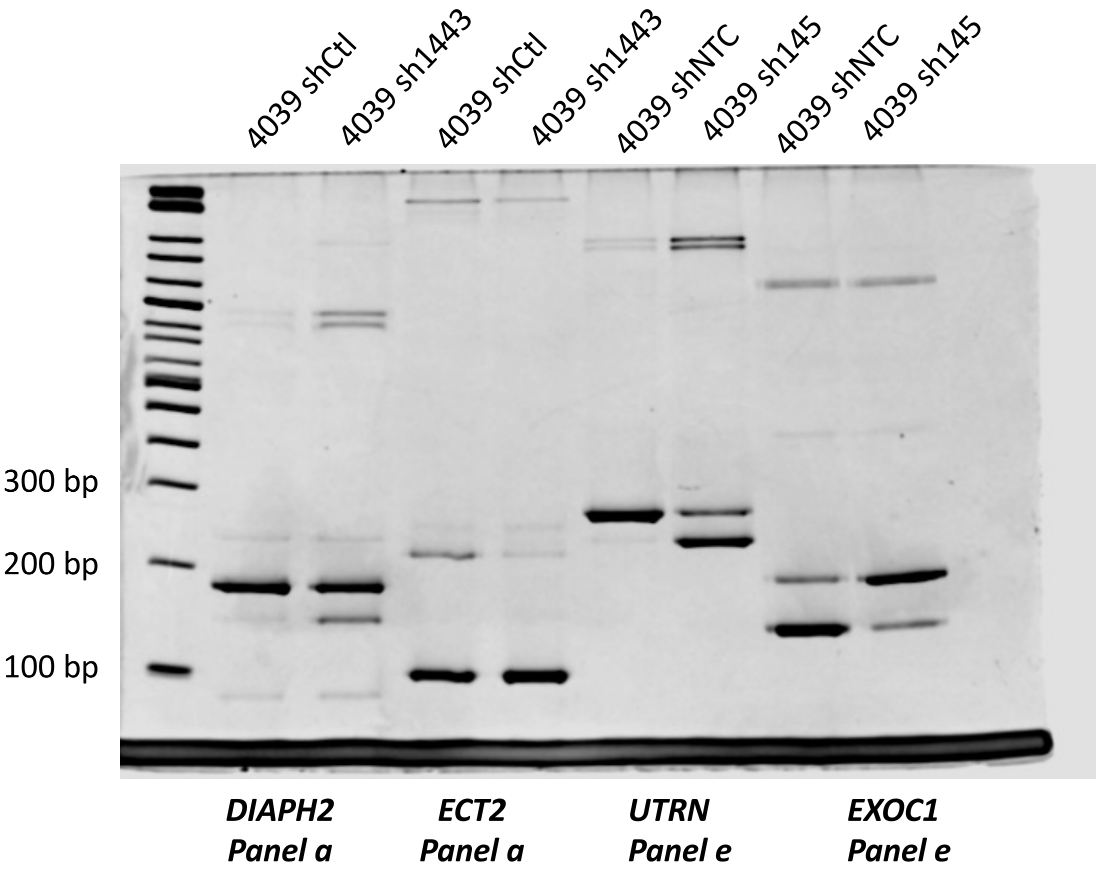

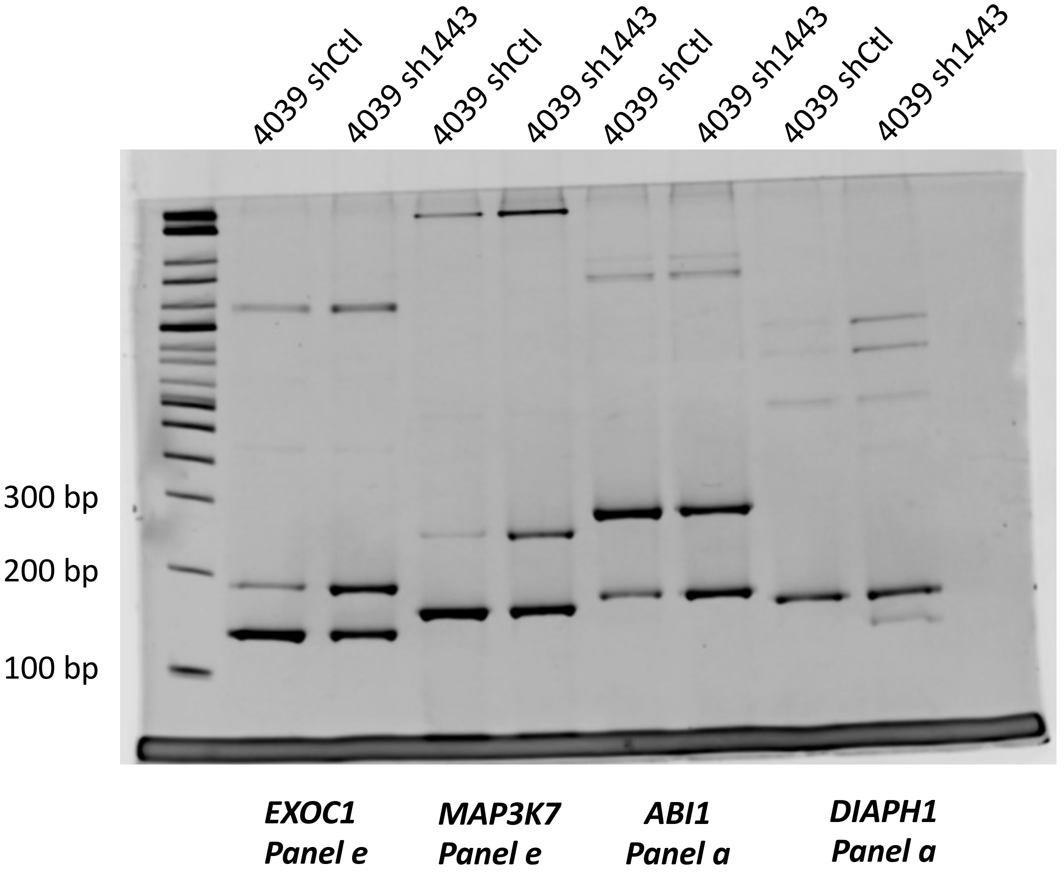

Supplemental Figure 10

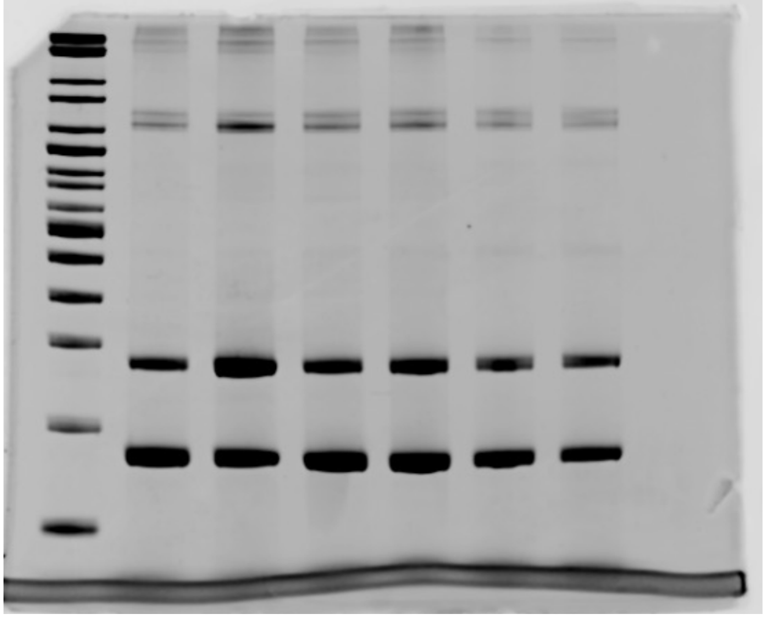

ABI1
Fig 6
panel d

ABI1
Supp Fig 10
panel b

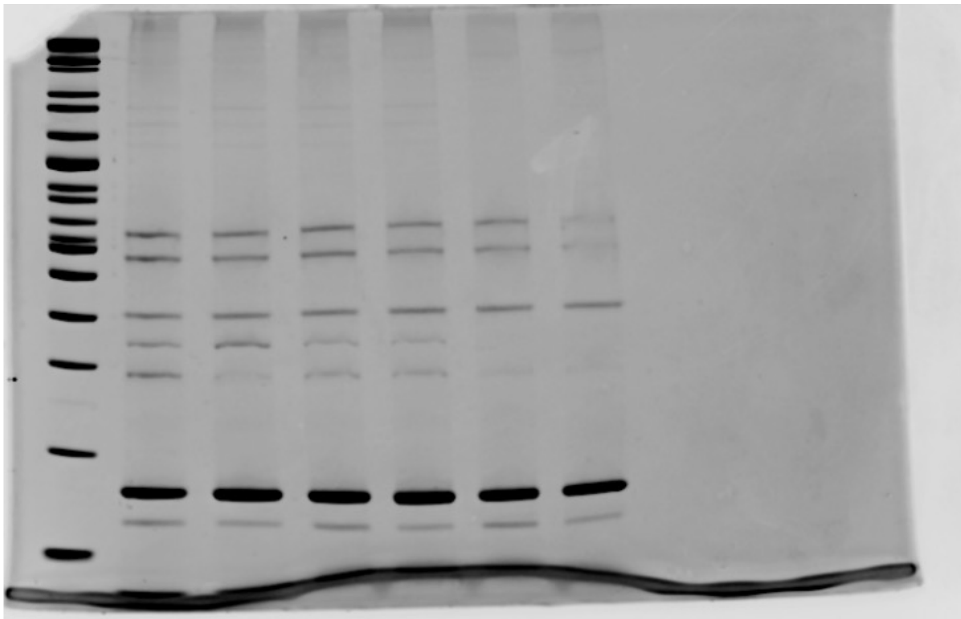

DIAPH1
Fig 6
panel d

DIAPH1
Supp Fig 10
panel b

Supplemental Figure 10

8902 **8902** **PATC148**

Ctl V1 Ctl V4 Ctl V4

300 bp
200 bp
100 bp

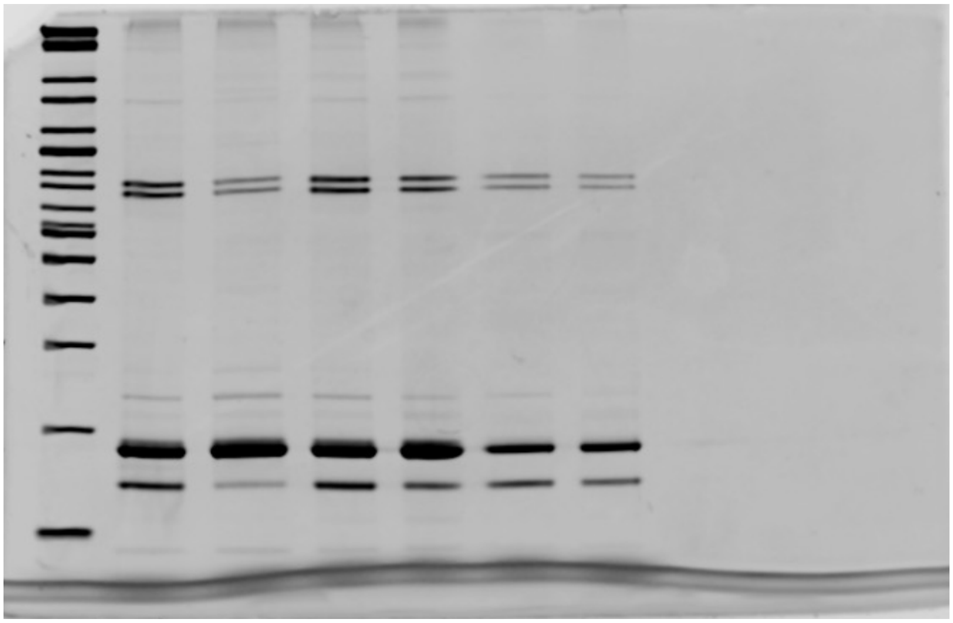

DIAPH2
Fig 6
panel d

DIAPH2
Supp Fig 10
panel b

8902 **8902** **PATC148**

Ctl V1 Ctl V4 Ctl V4

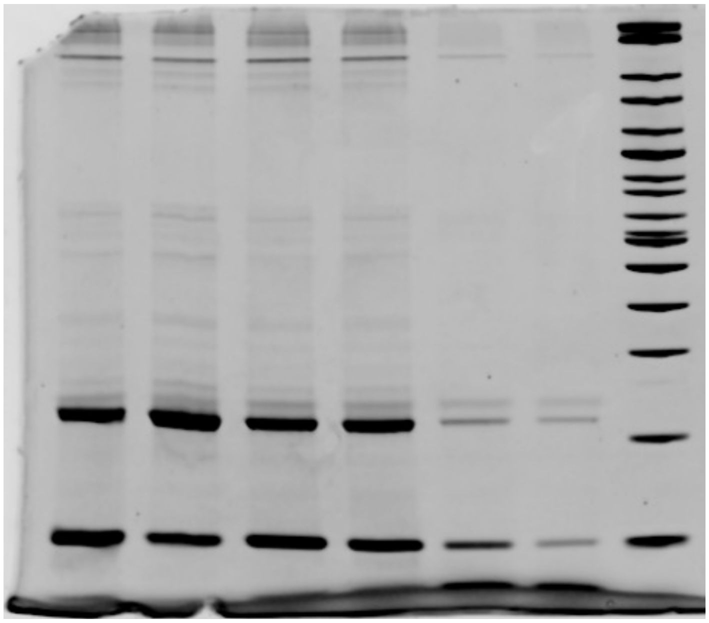

300 bp
200bp
100bp

ECT2
Fig 6
panel d

ECT2
Supp Fig 10
panel b

Supplemental Figure 10

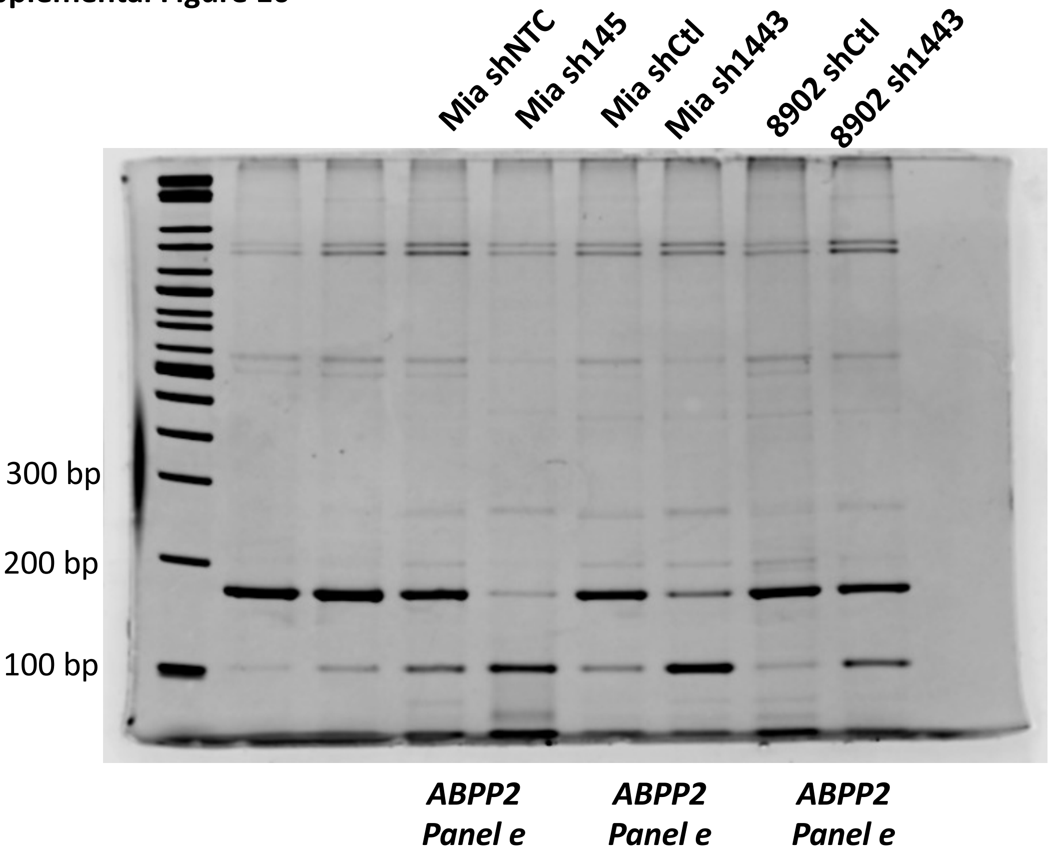

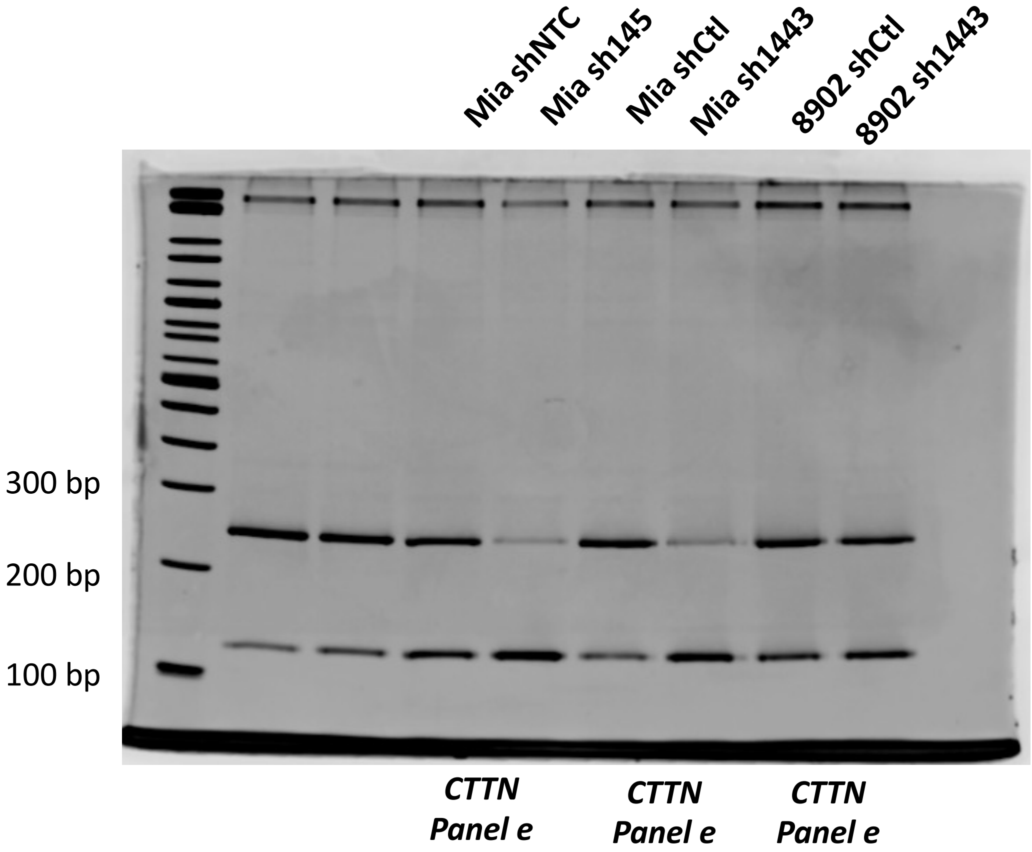

Supplemental Figure 10

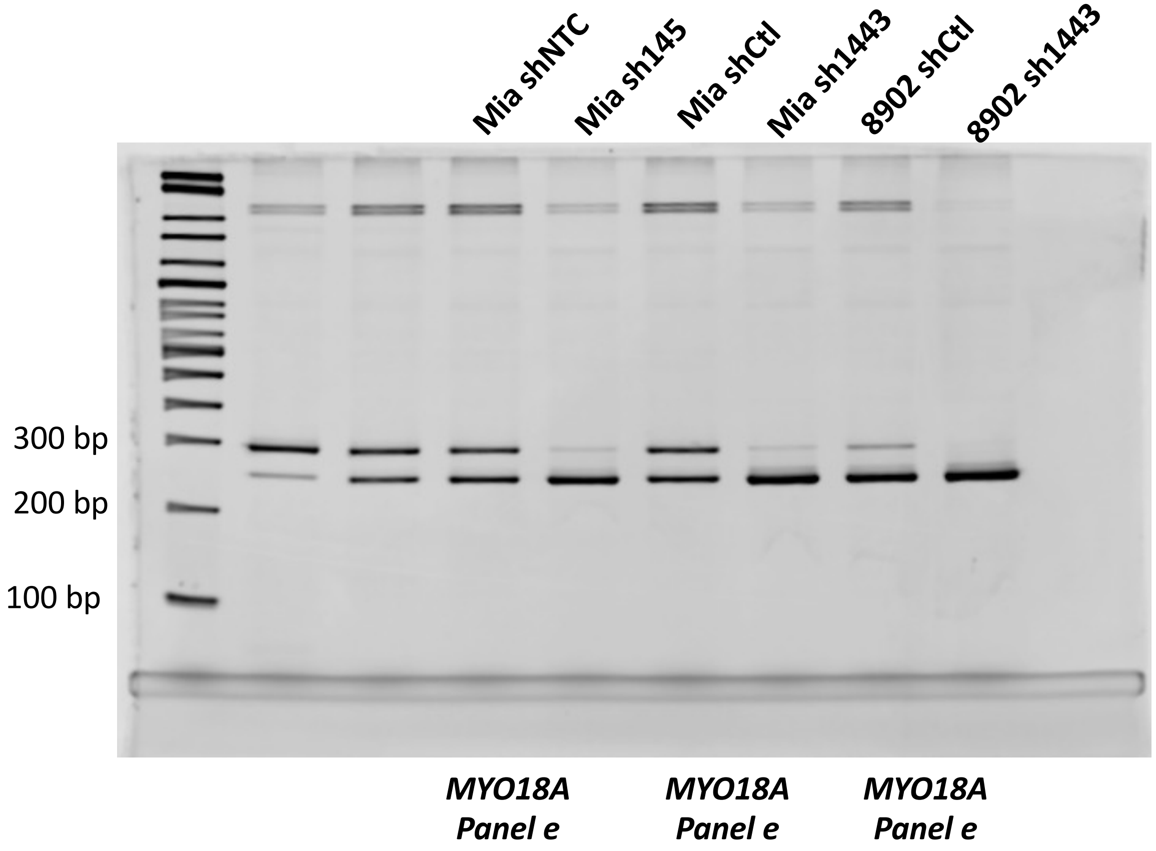

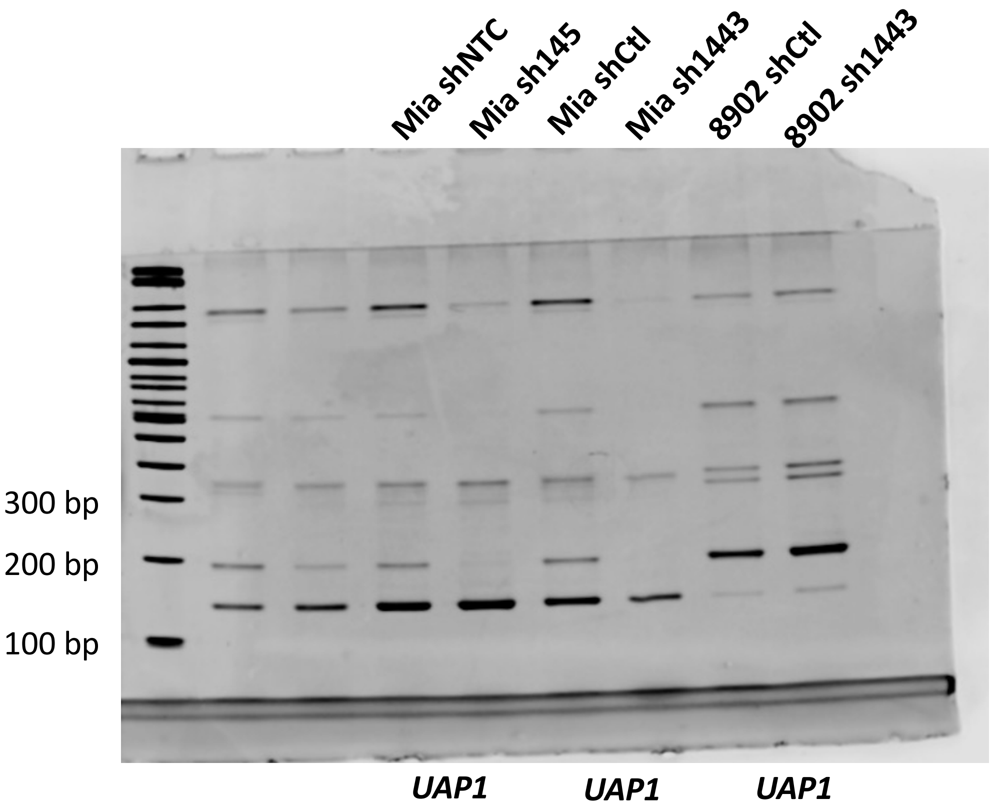

Supplemental Figure 10

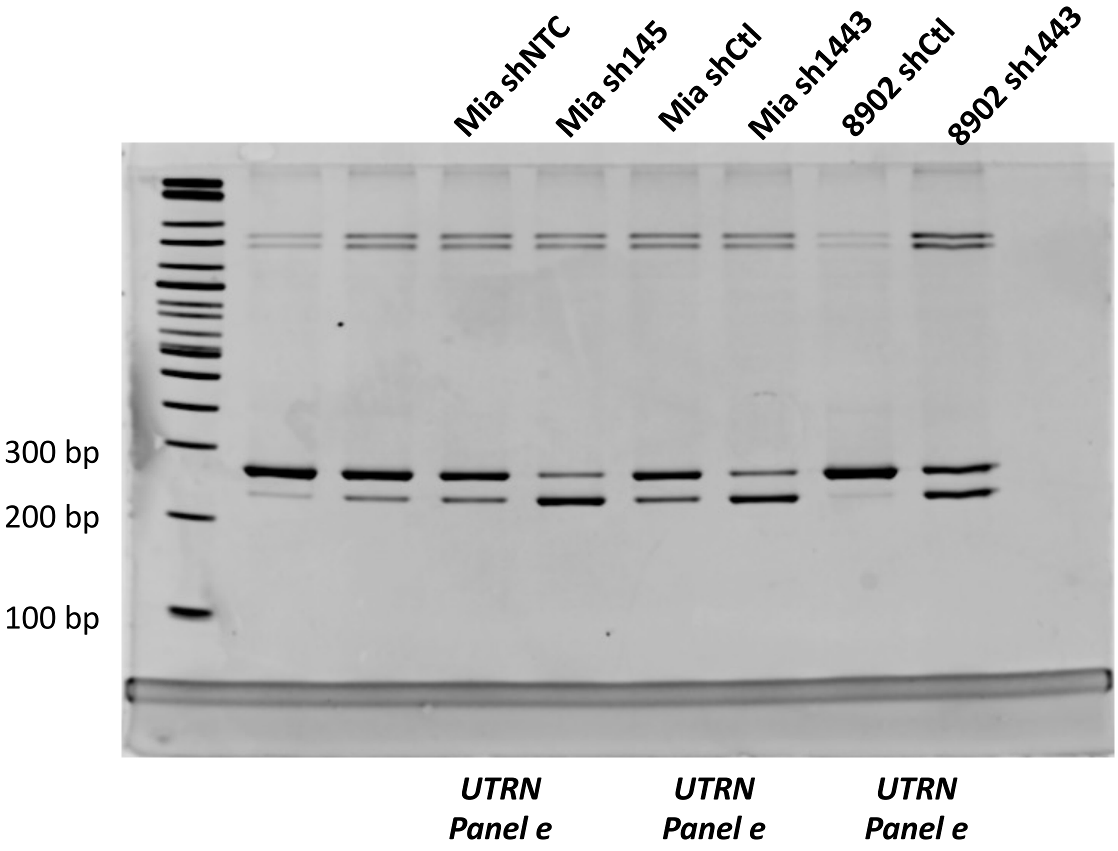

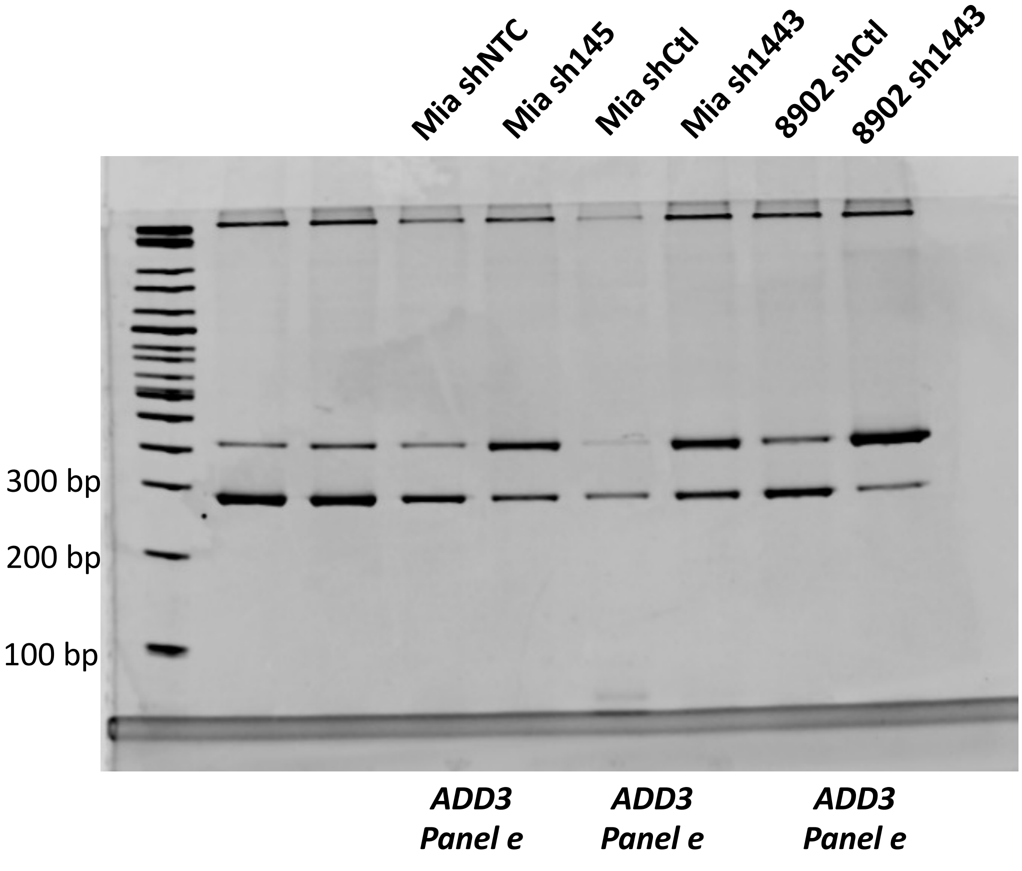

Supplemental Figure 10

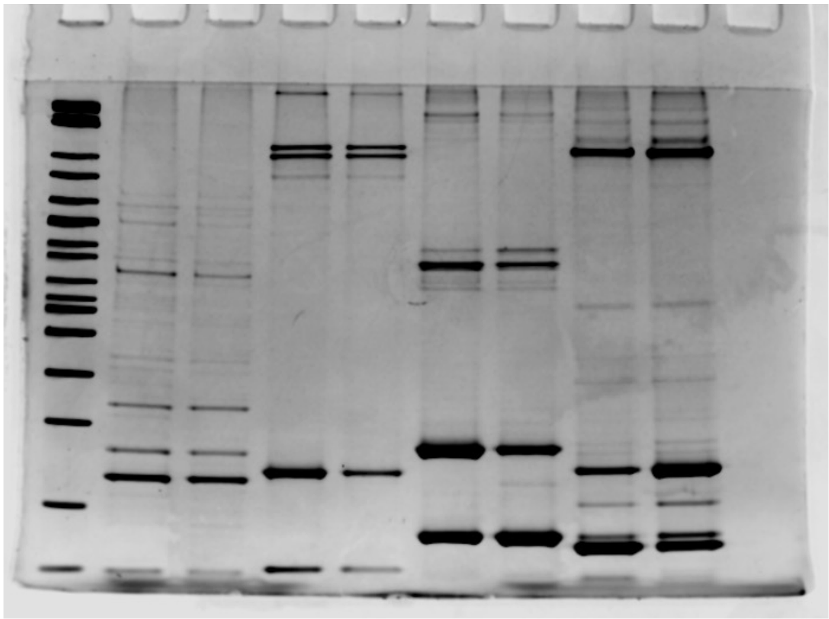

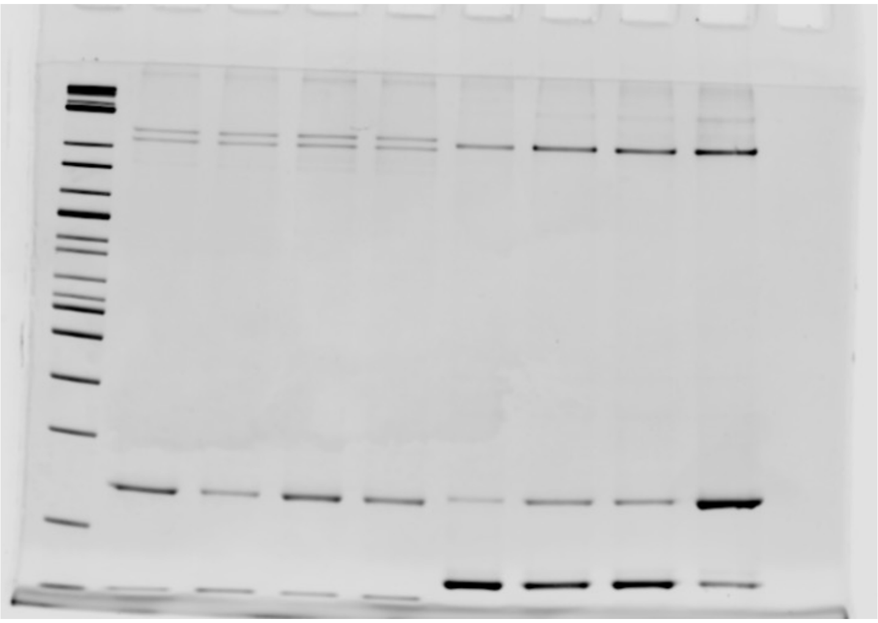

Supplemental Figure 10

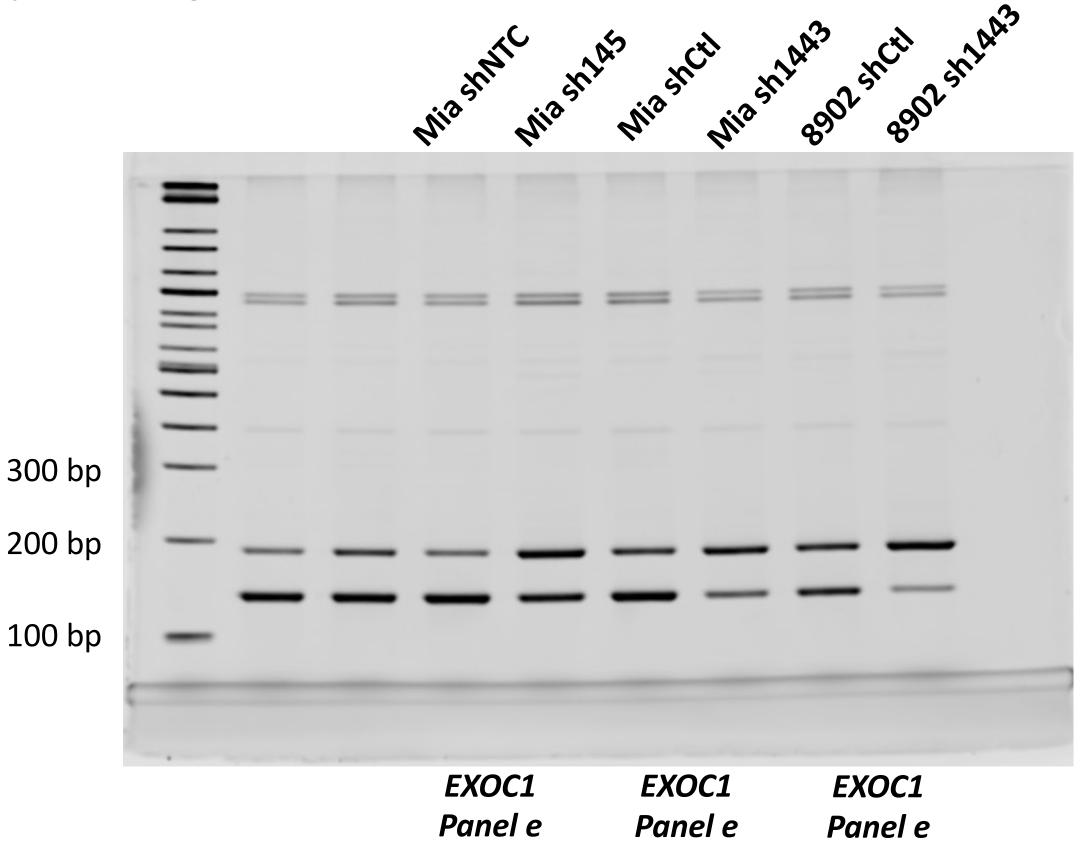

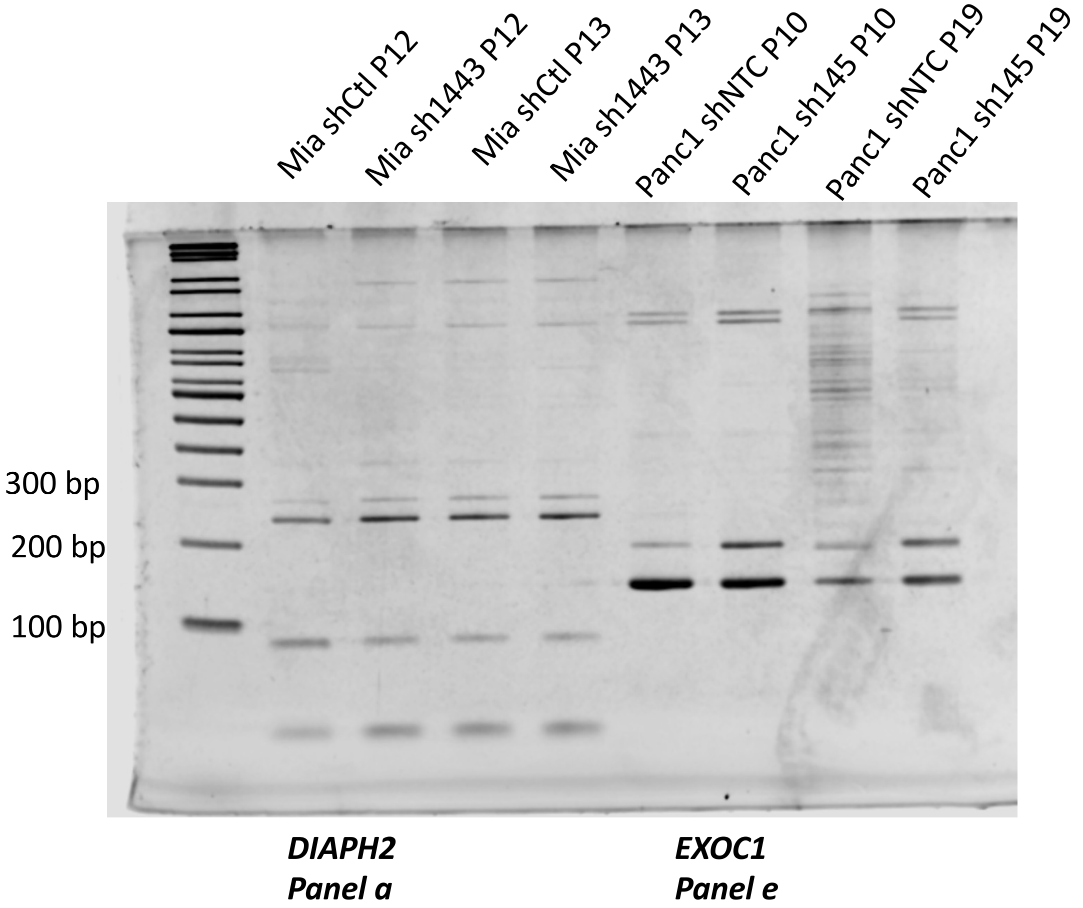

Supplemental Figure 10

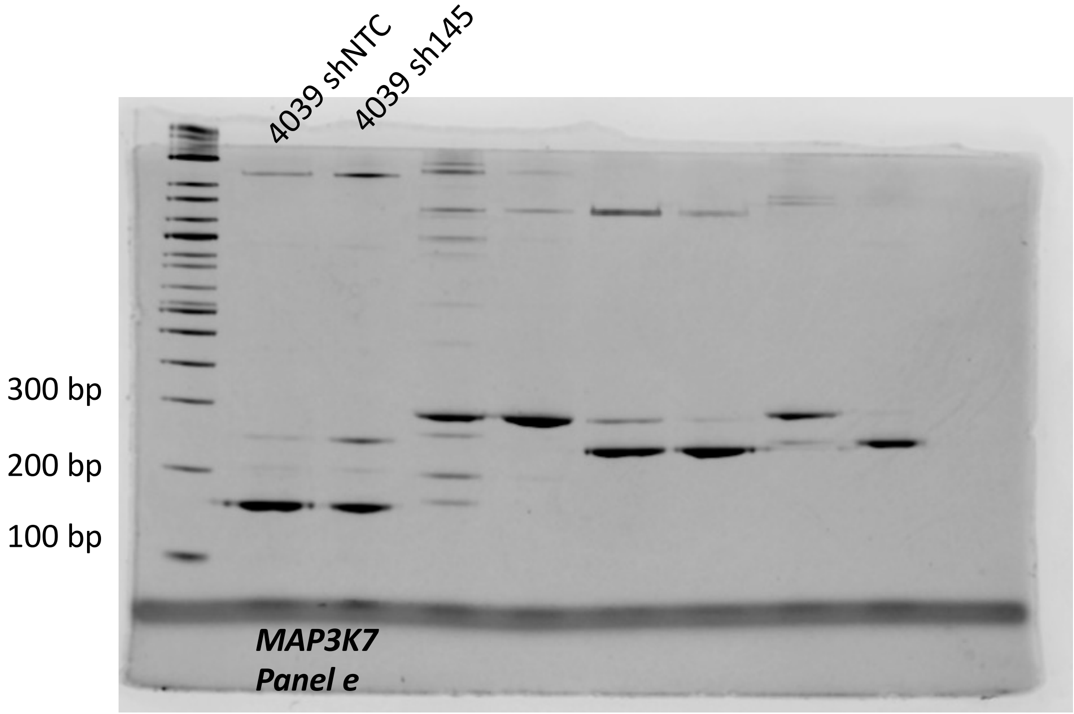

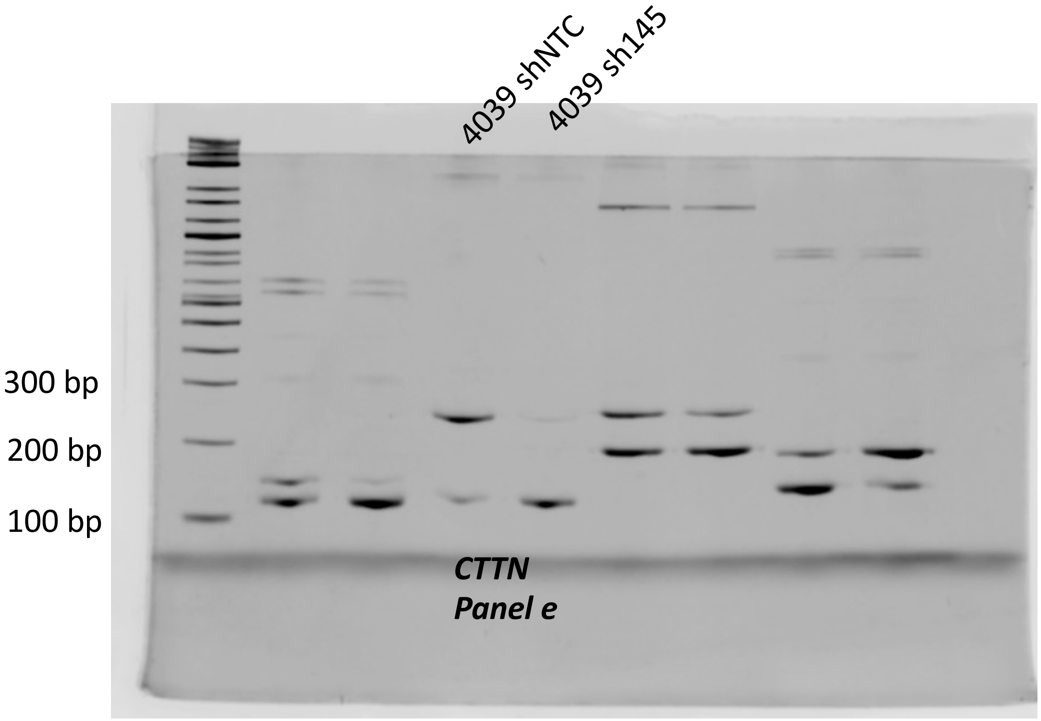

Supplemental Figure 10

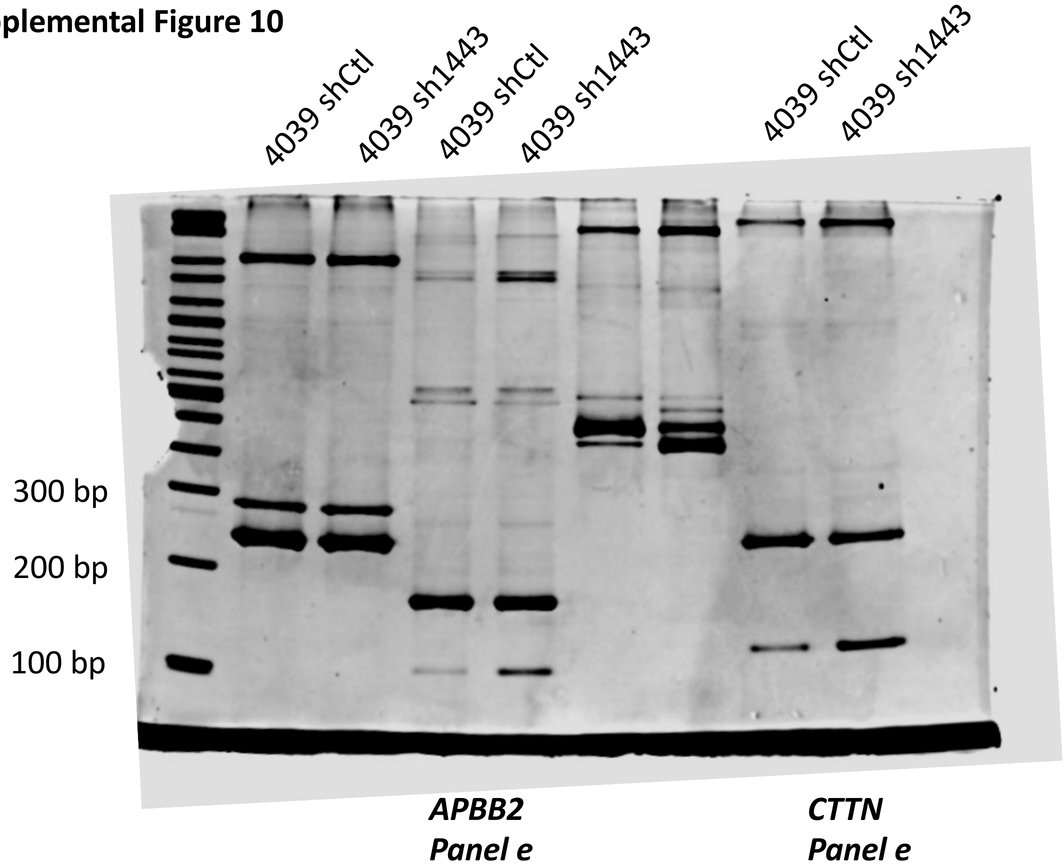

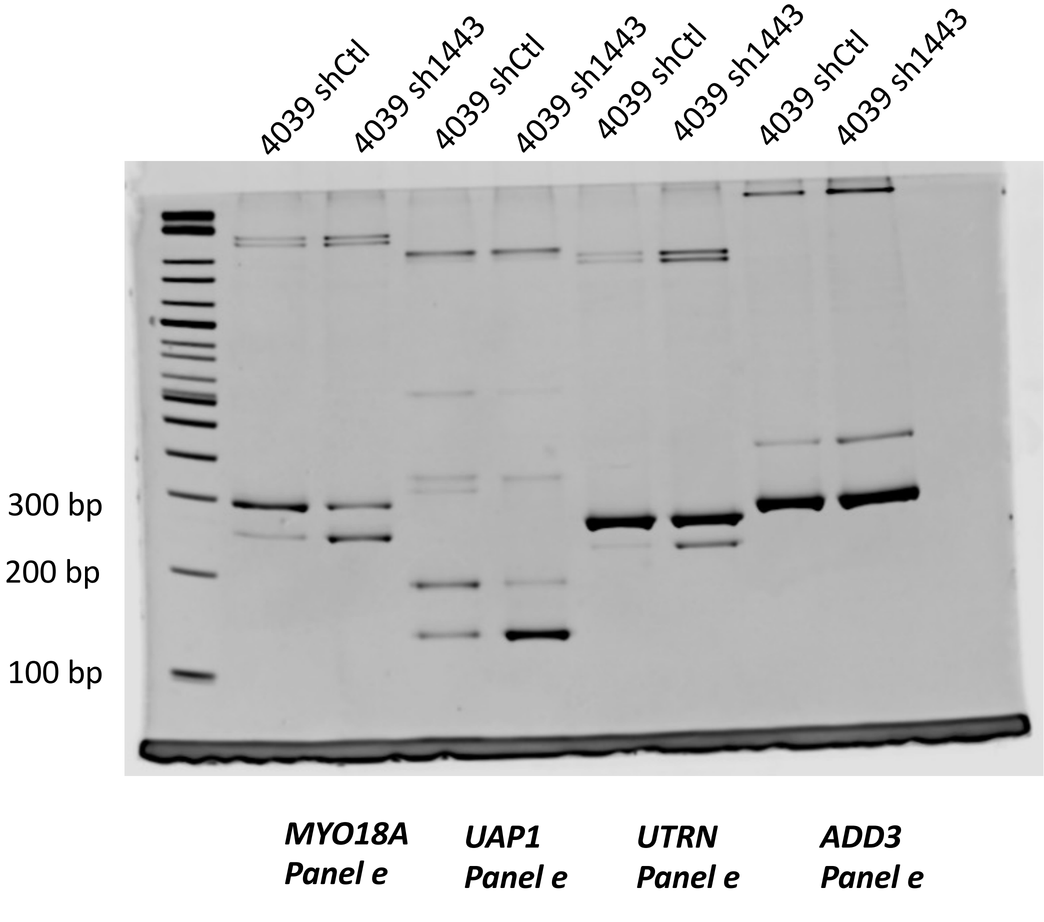

Supplemental Figure 10

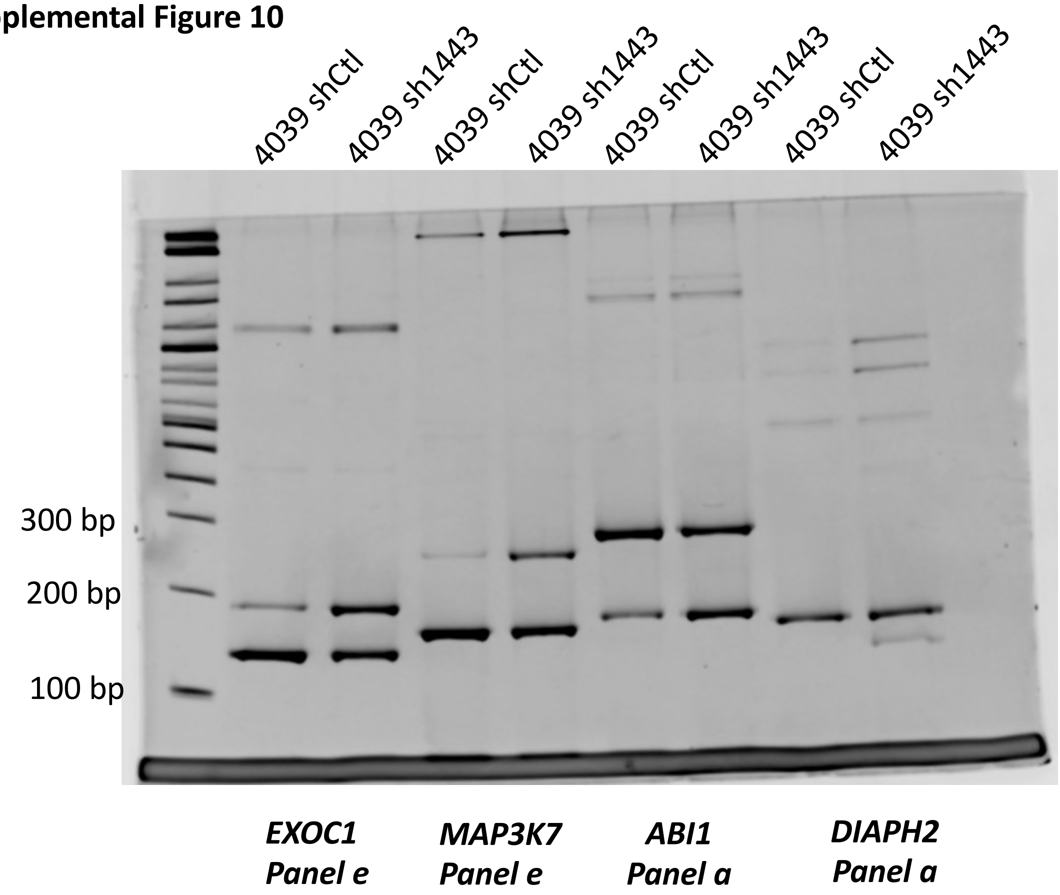

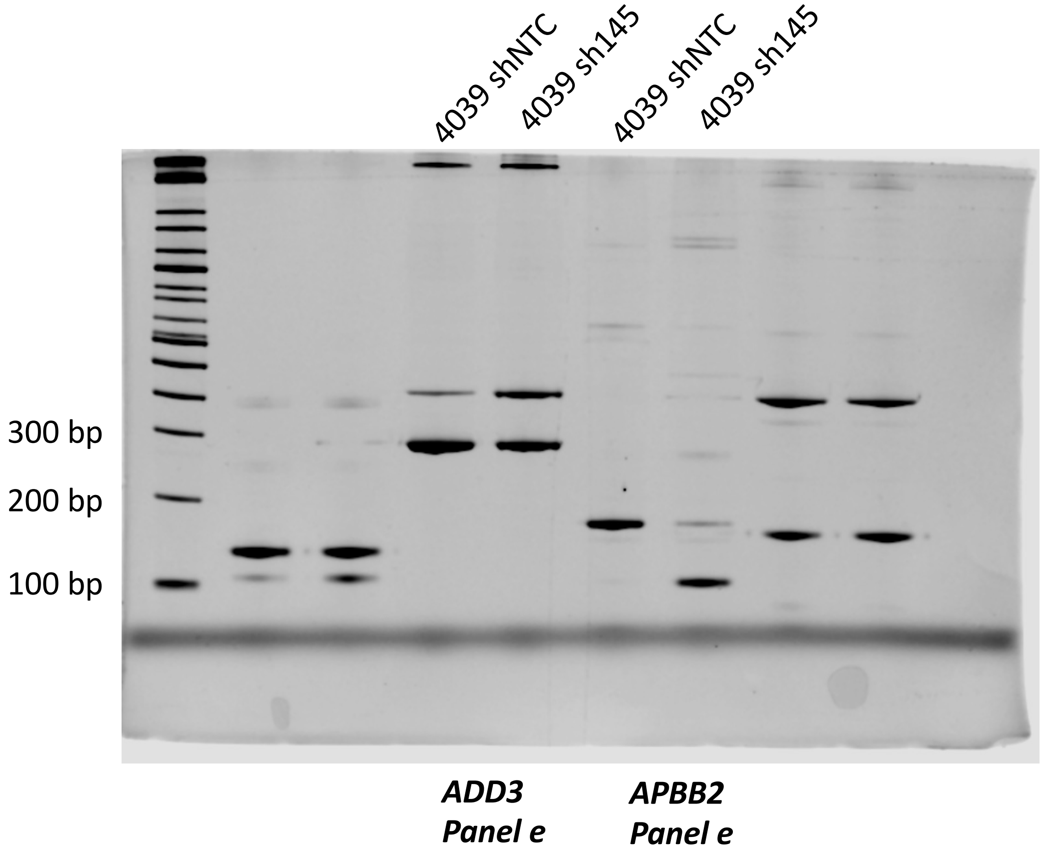

Supplemental Figure 10

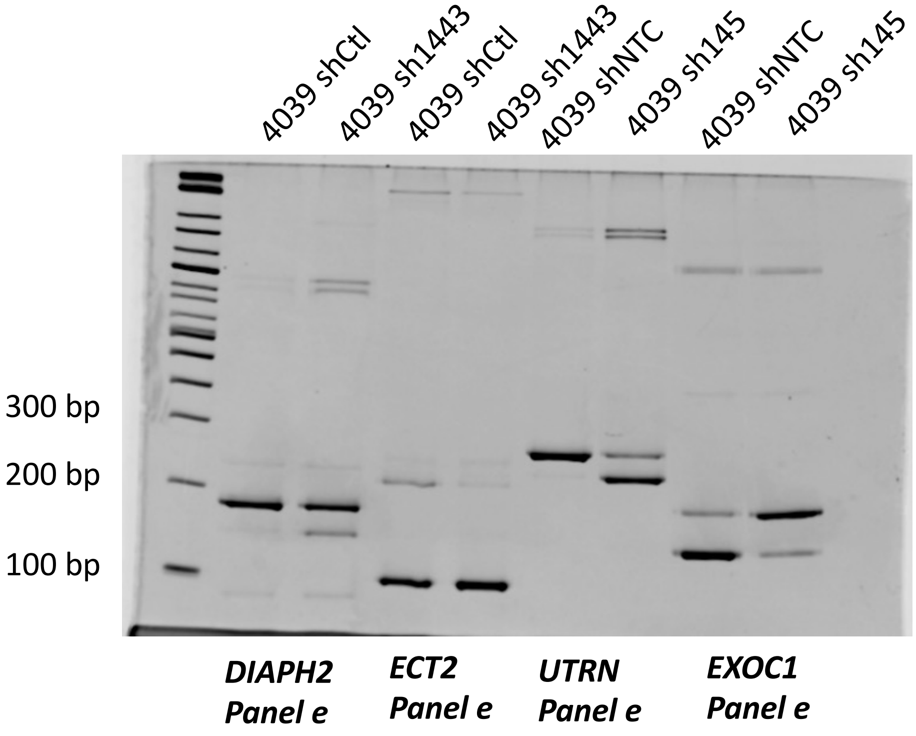

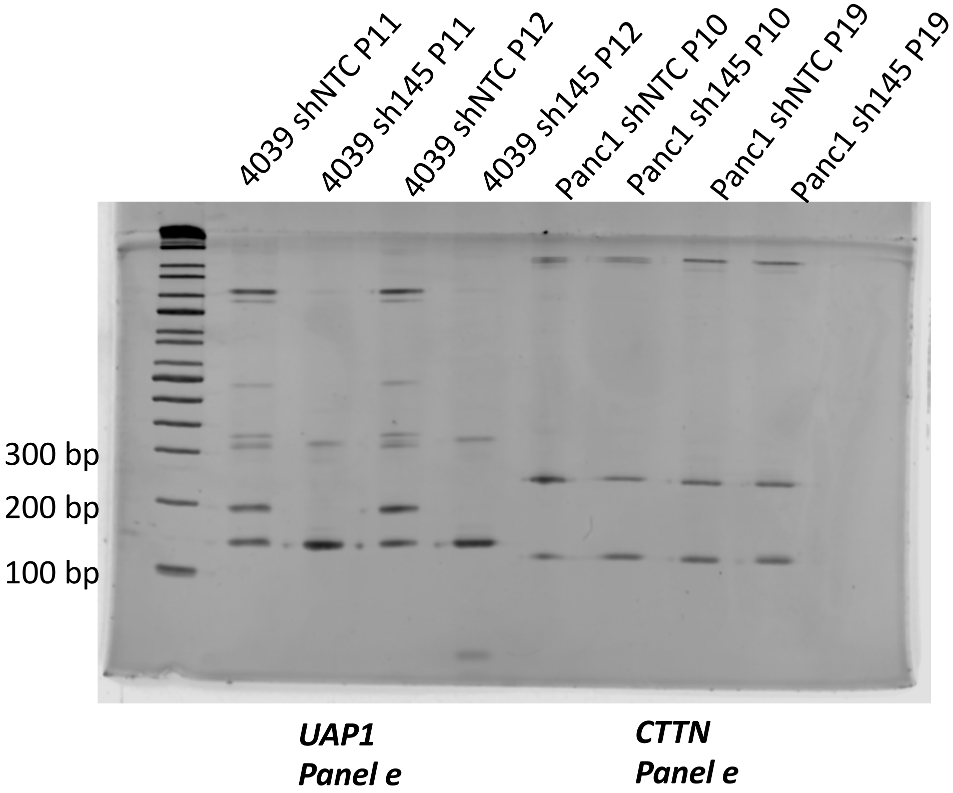

Supplemental Figure 10

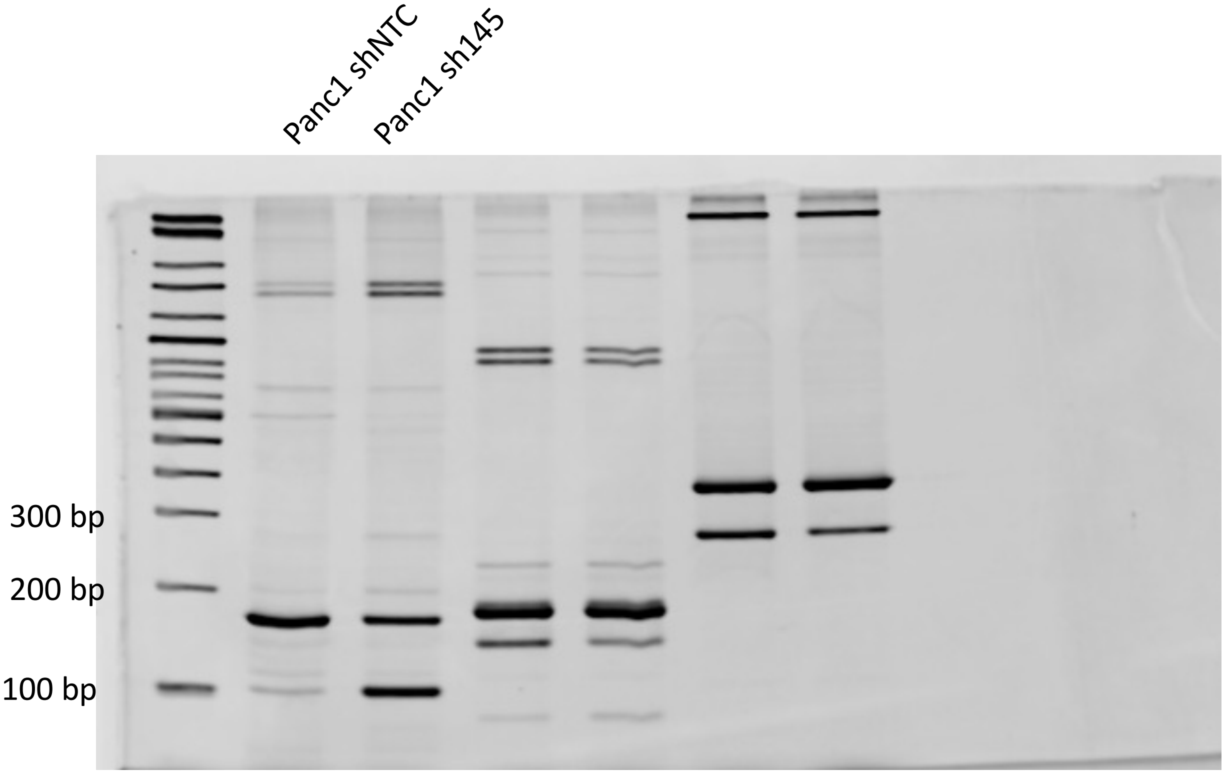

APBB2
Panel d

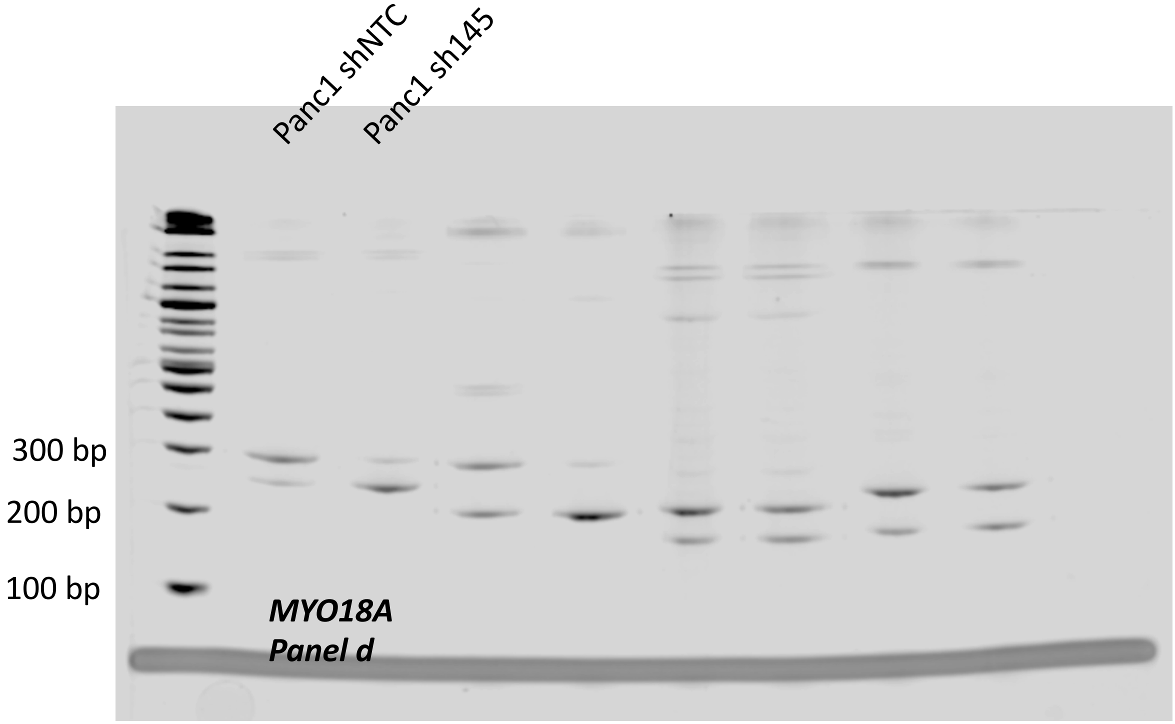

Supplemental Figure 10

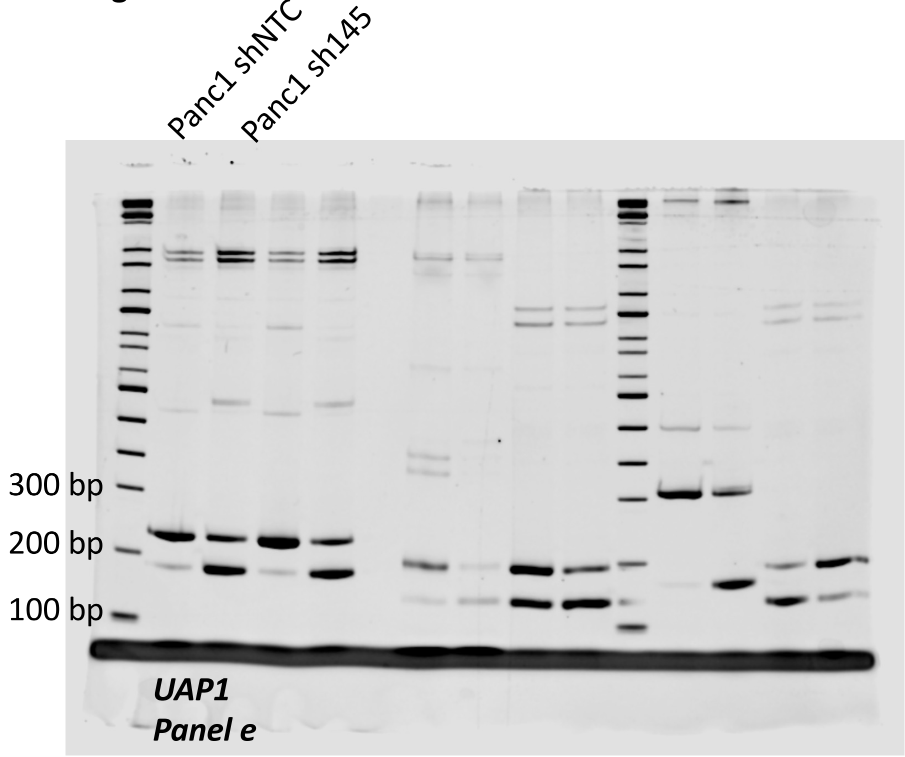

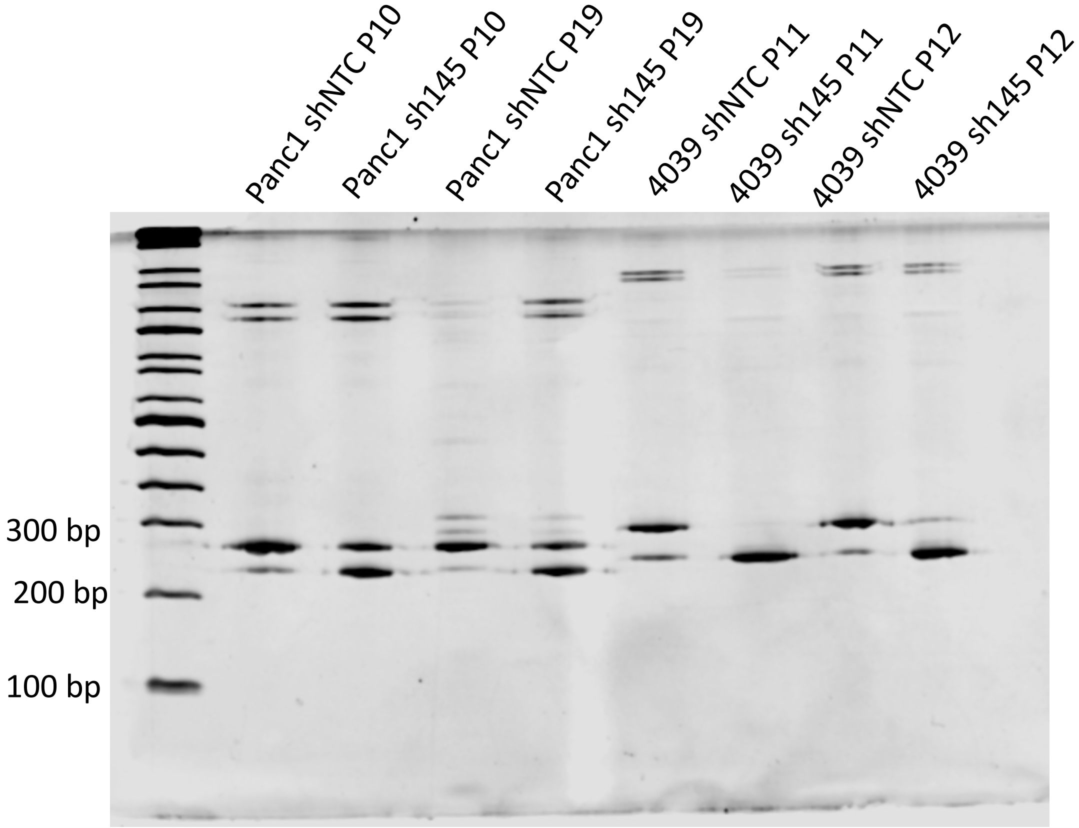

***UTRN* Panel e**

***MYO18A* Panel e**

Supplemental Figure 10

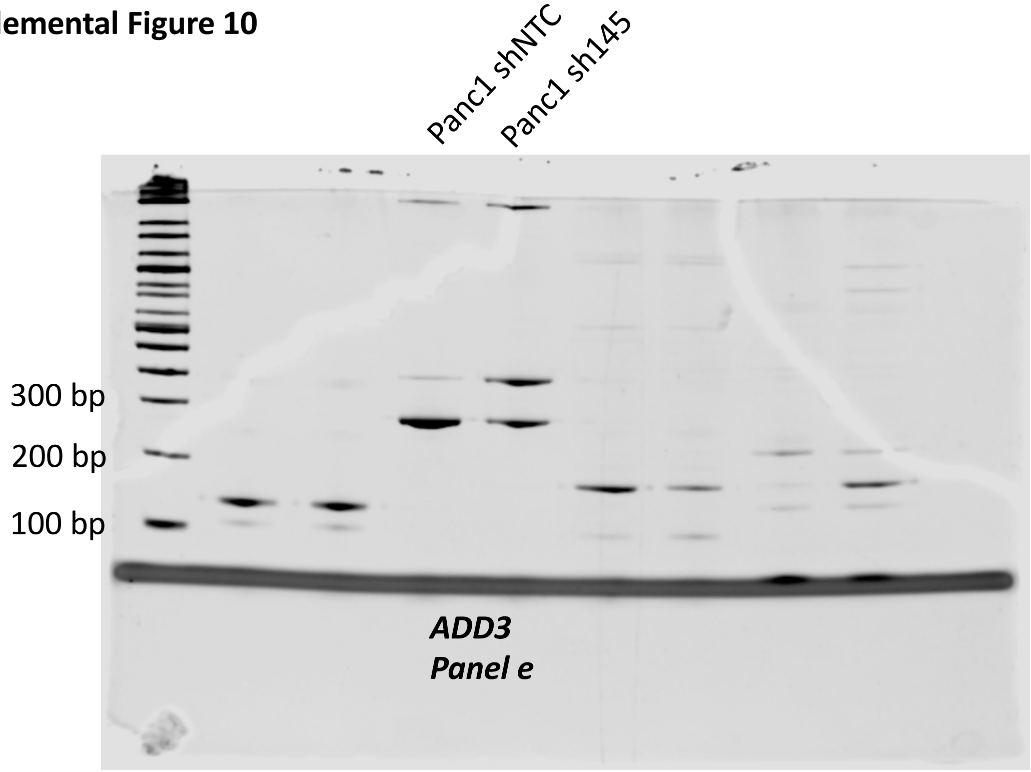

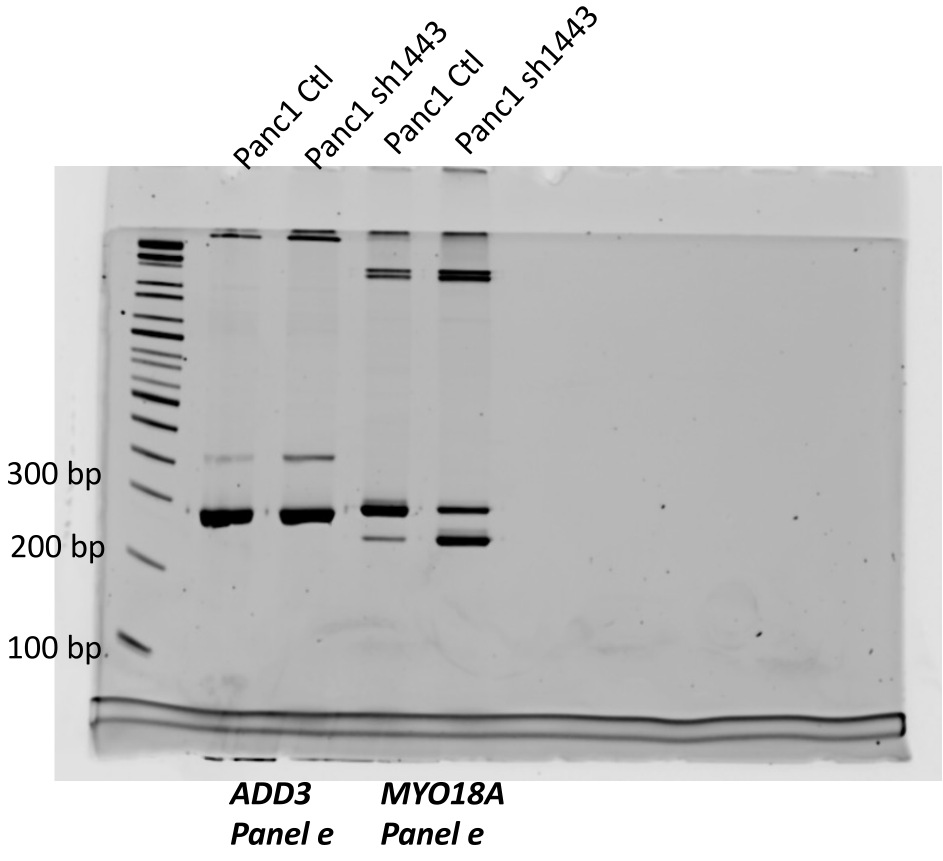

Supplemental Figure 10

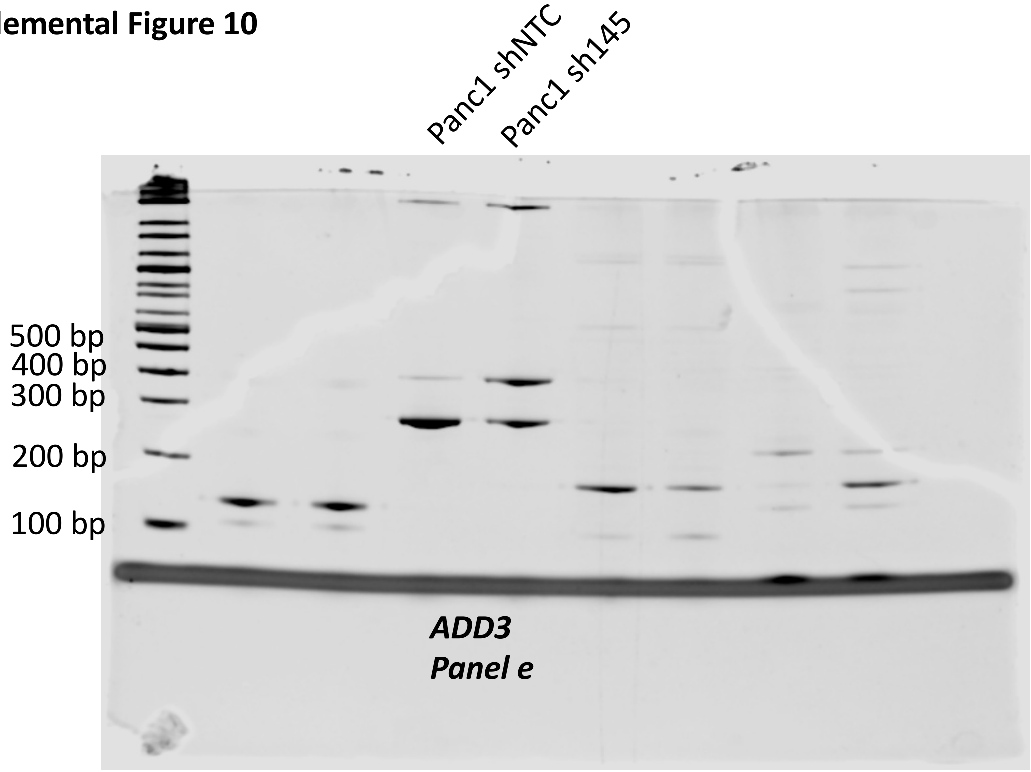

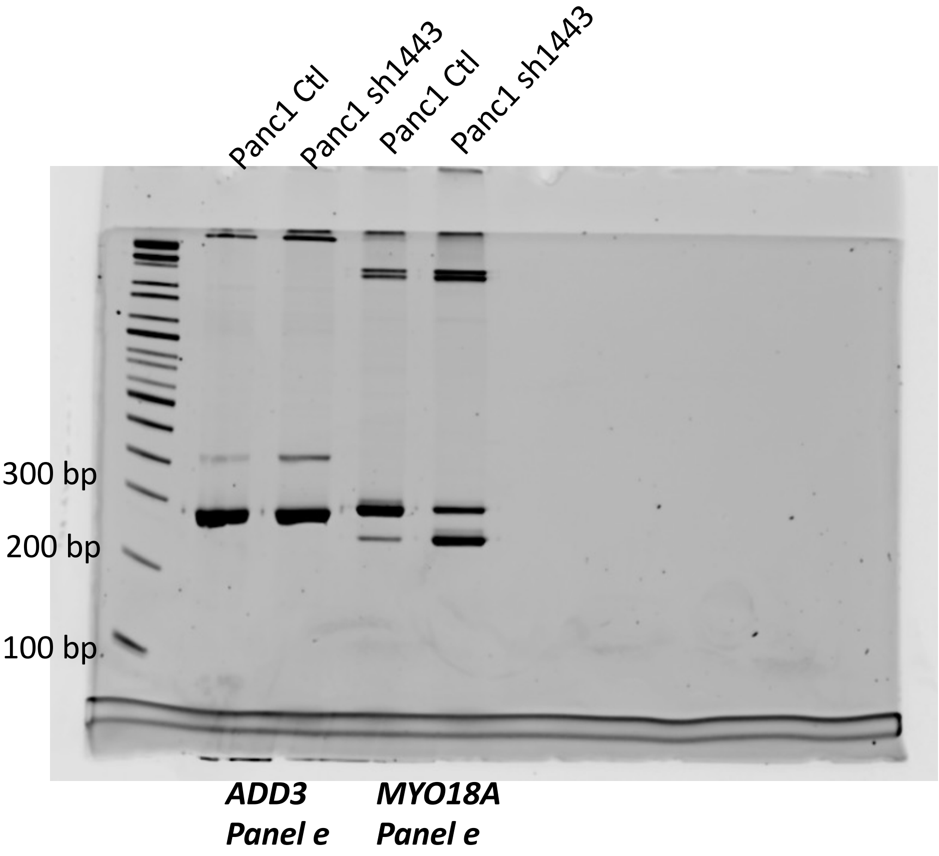

Supplemental Figure 10

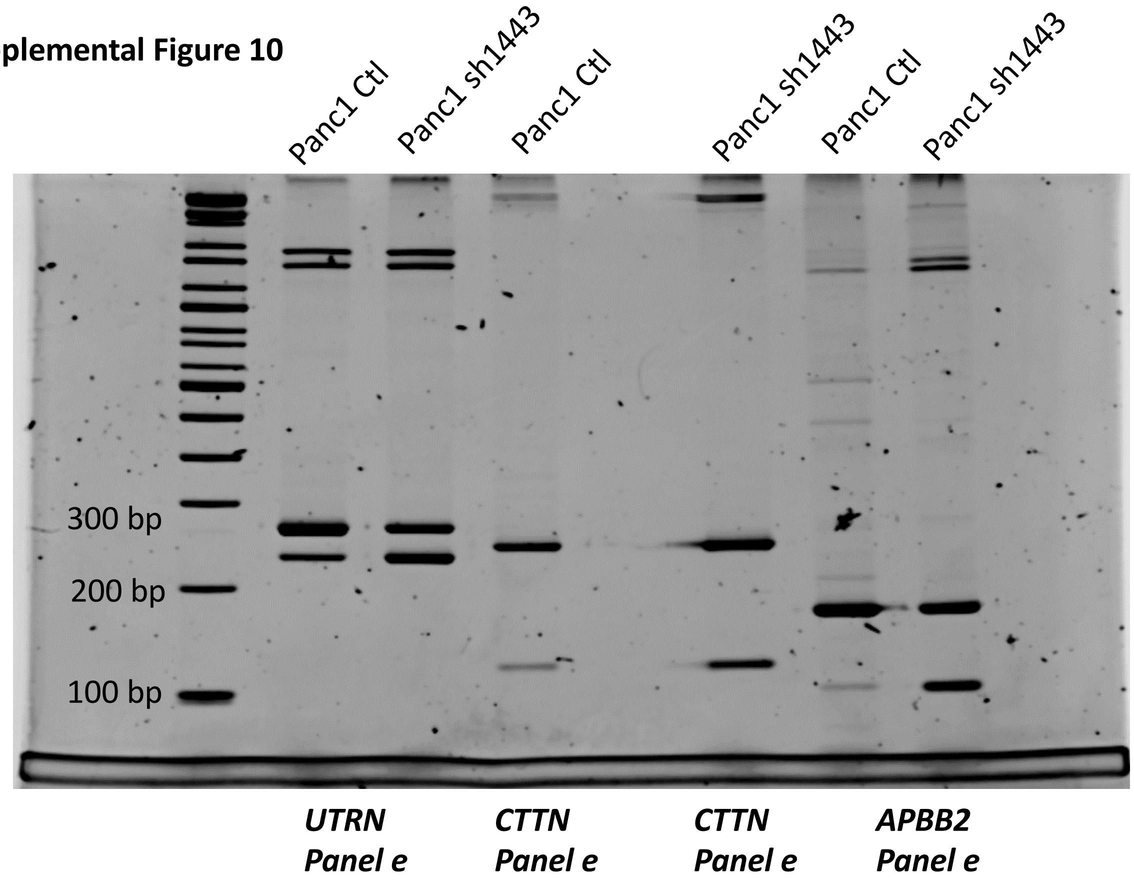

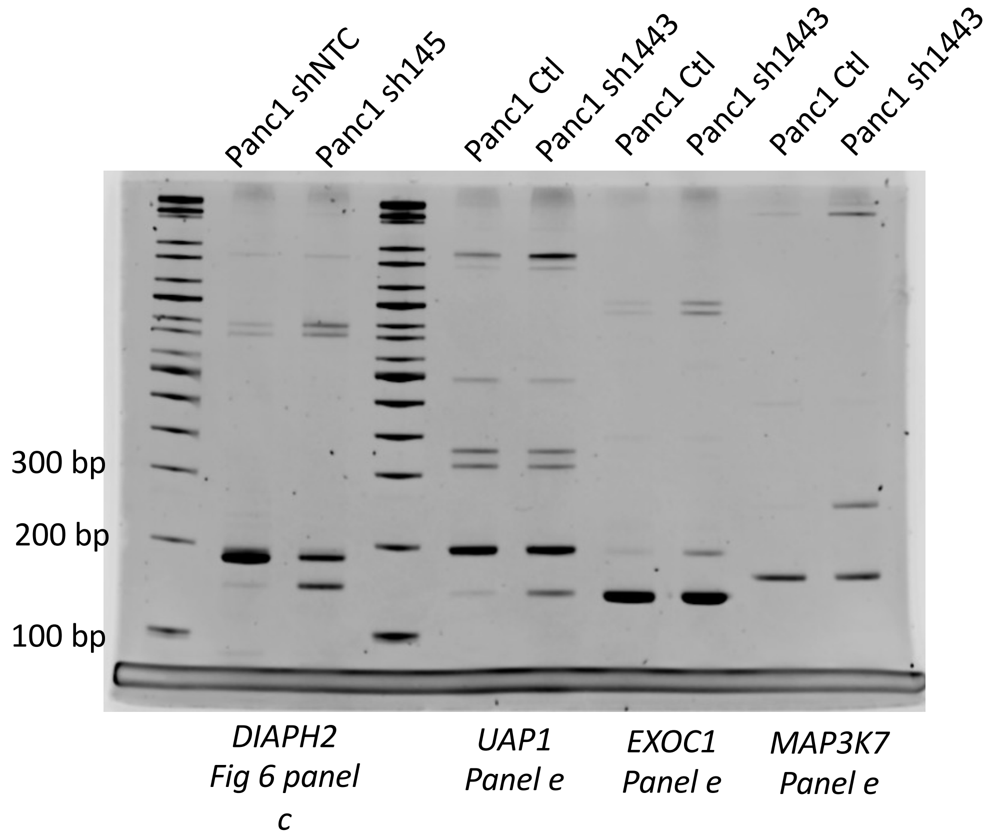

Supplemental Figure 10

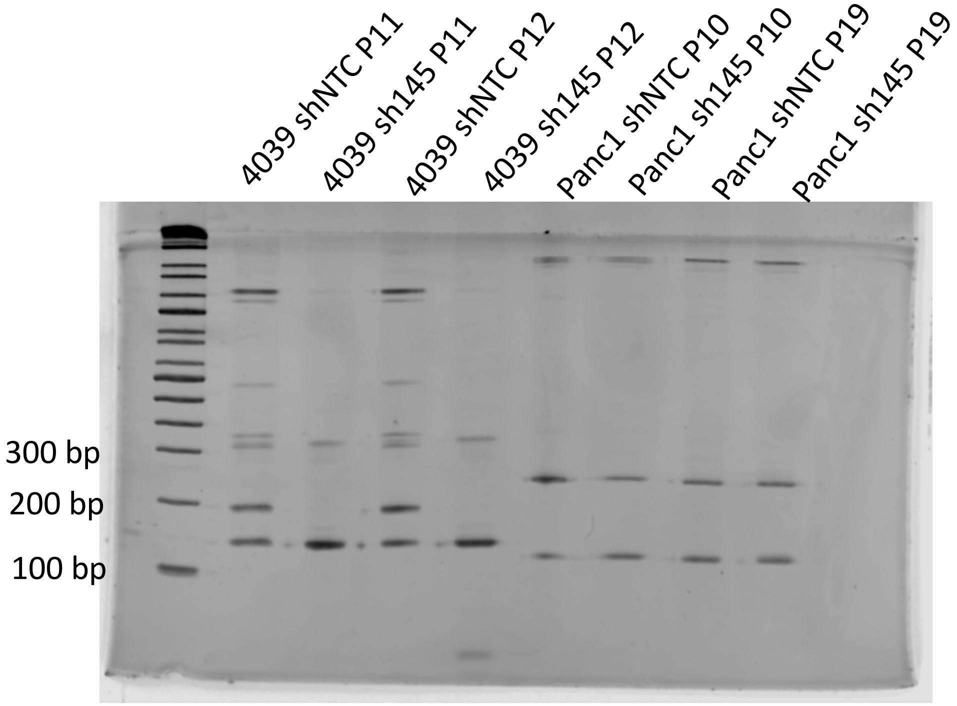

***UAP1* Panel e** ***CTTN* panel e**

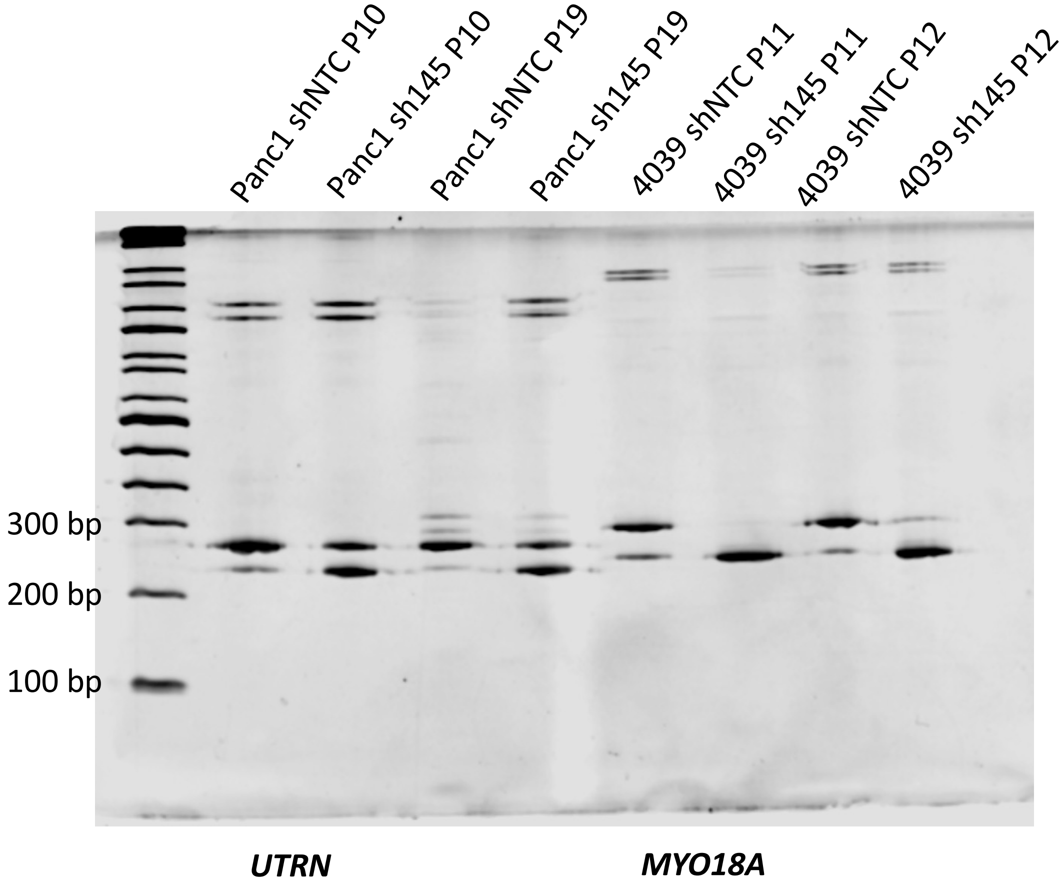

***UTRN* Panel e** ***MYO18A* Panel e**

Supplemental Figure 10

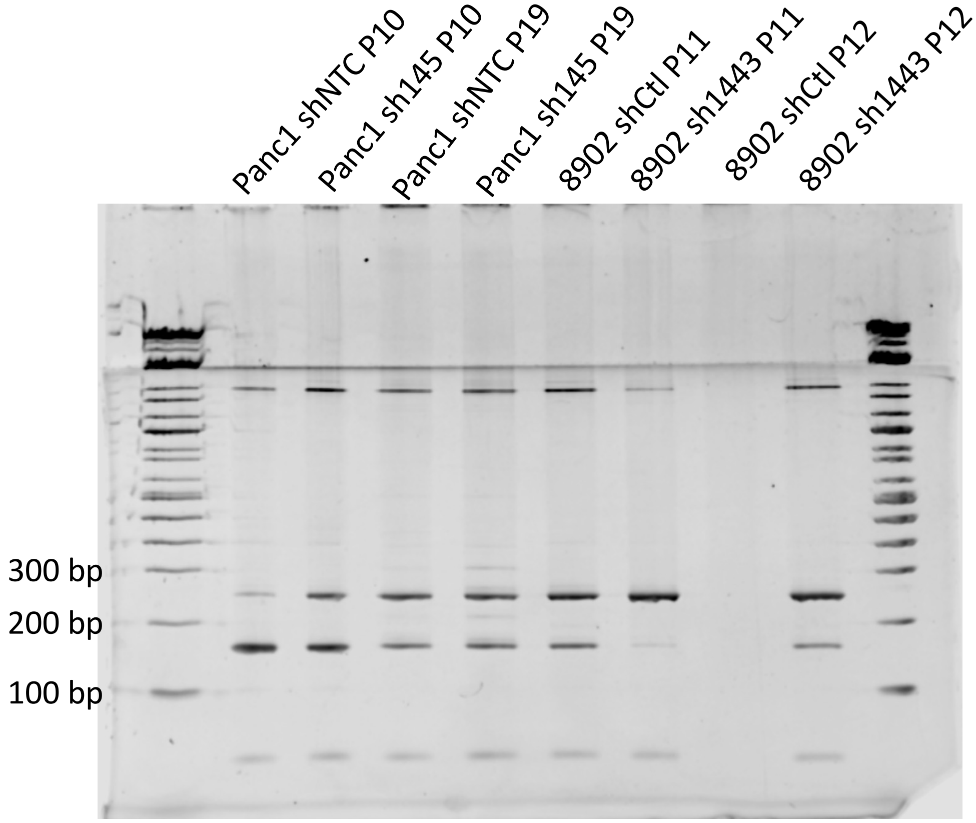

MAP3K7
Panel e

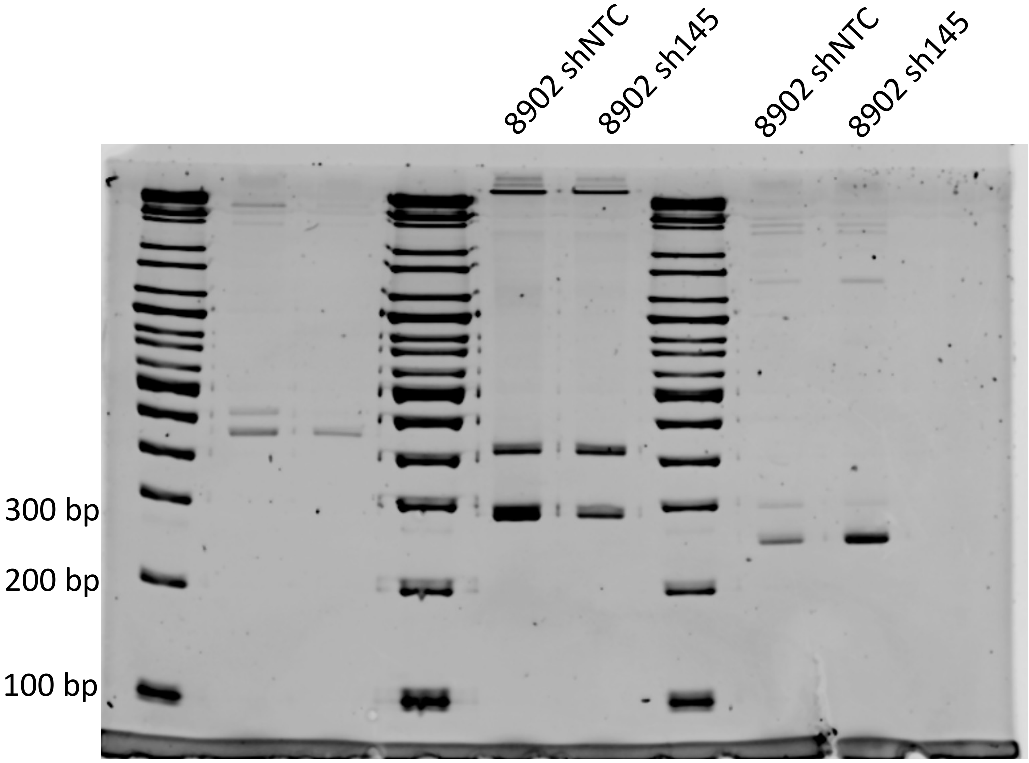

ADD3 ***MYO18A***
Panel e ***Panel e***

Supplemental Figure 10

8902 shNTC 8902 sh145

300 bp
200 bp
100 bp

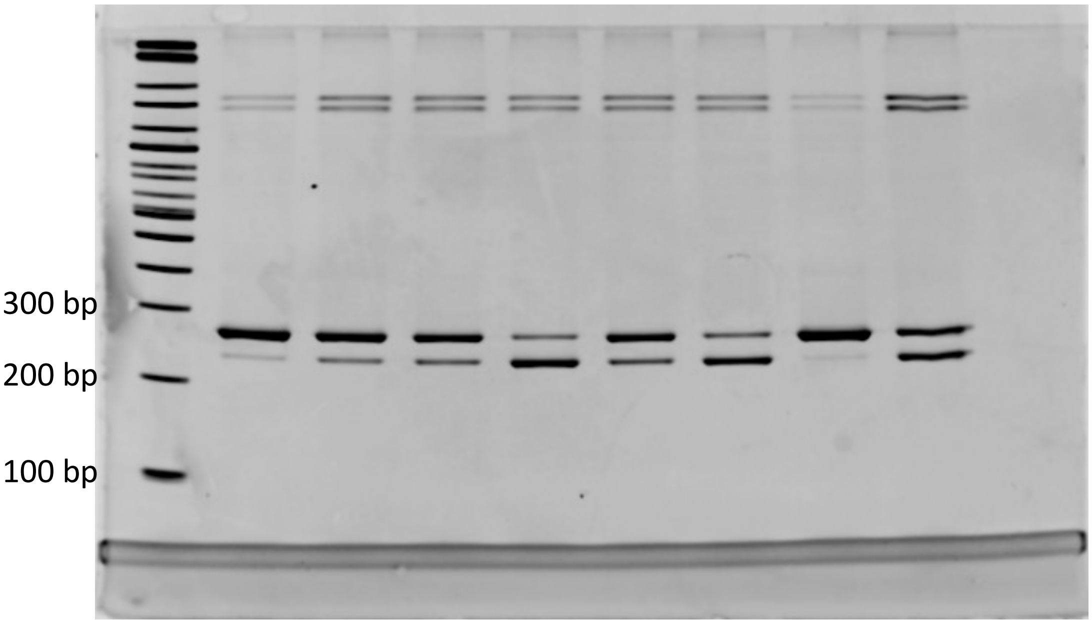

UTRN
Panel e

8902 shNTC 8902 sh145 8902 shNTC 8902 sh145 8902 shNTC 8902 sh145

300 bp
200 bp
100 bp

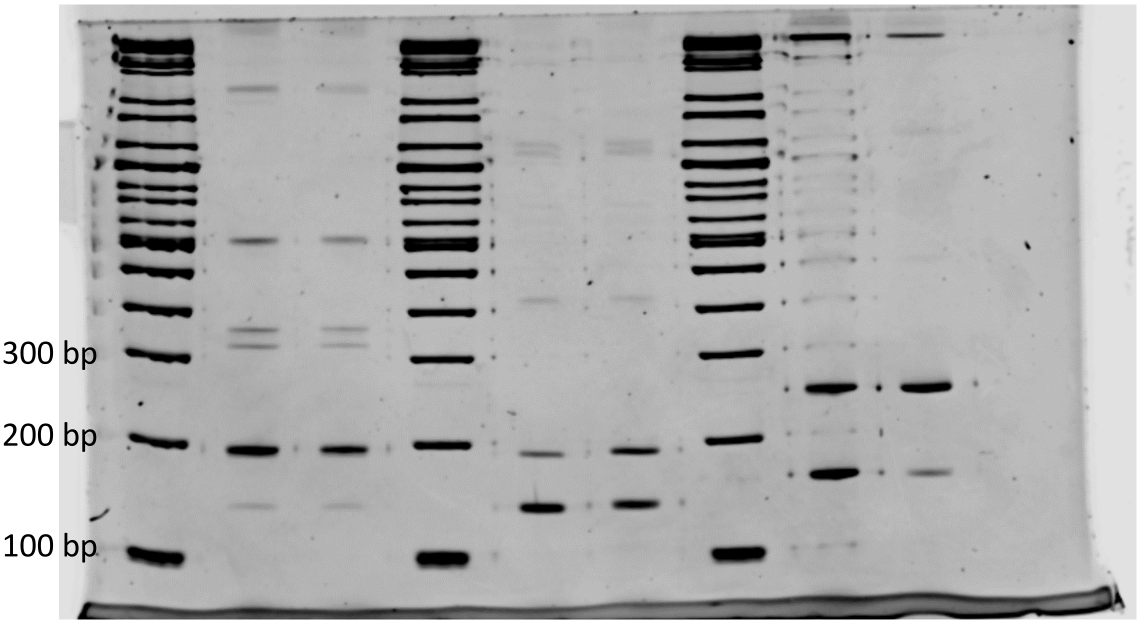

UAP1
Panel e

MAP3K7
Panel e

Supplemental Figure 10

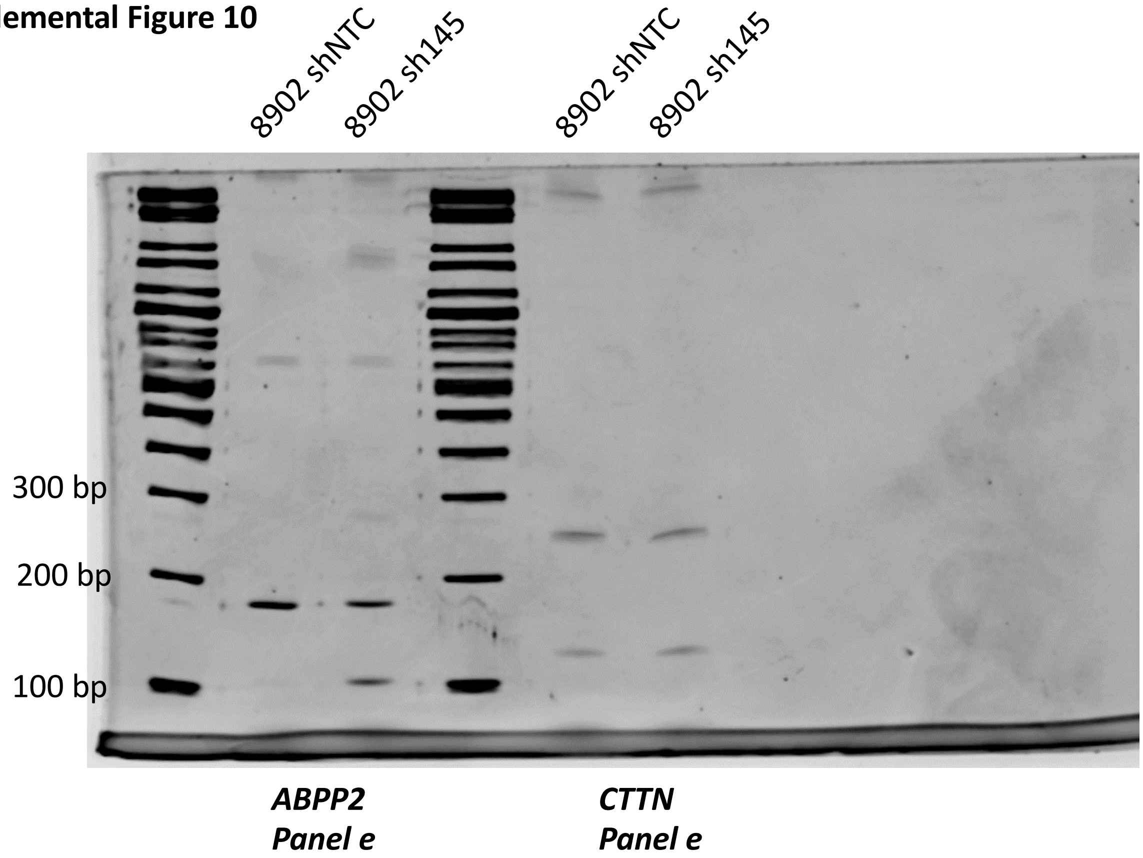

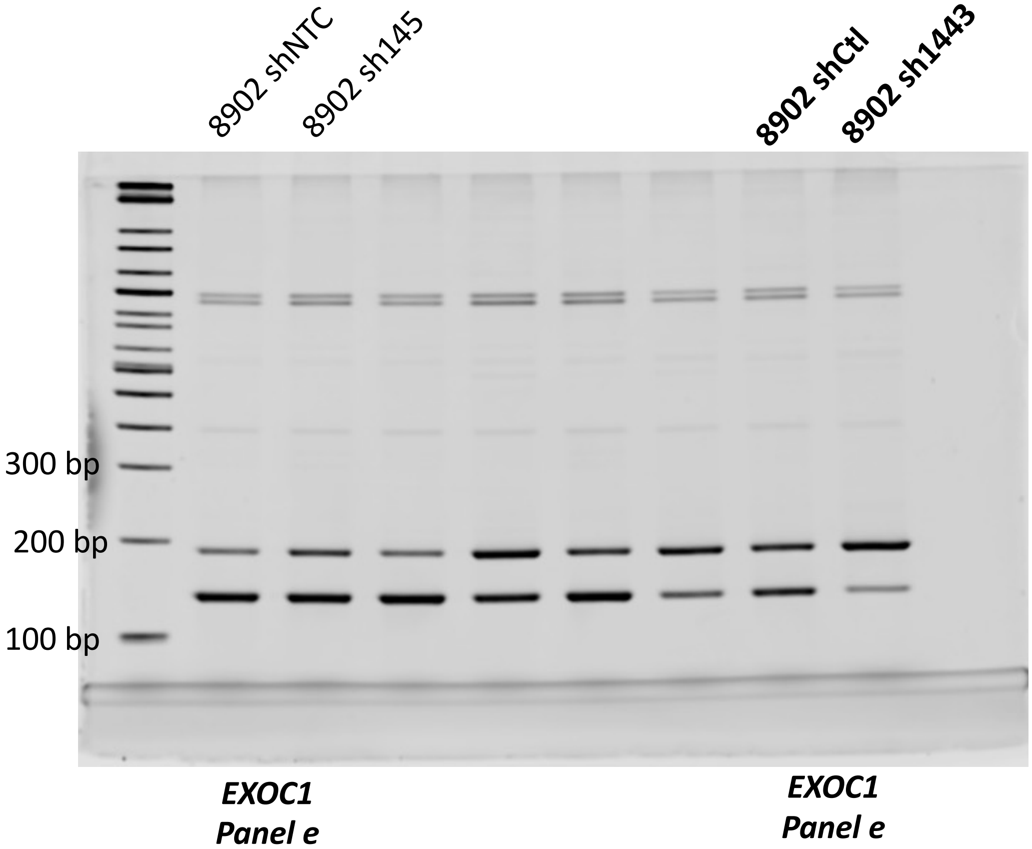

Supplemental Figure 12

Panel a

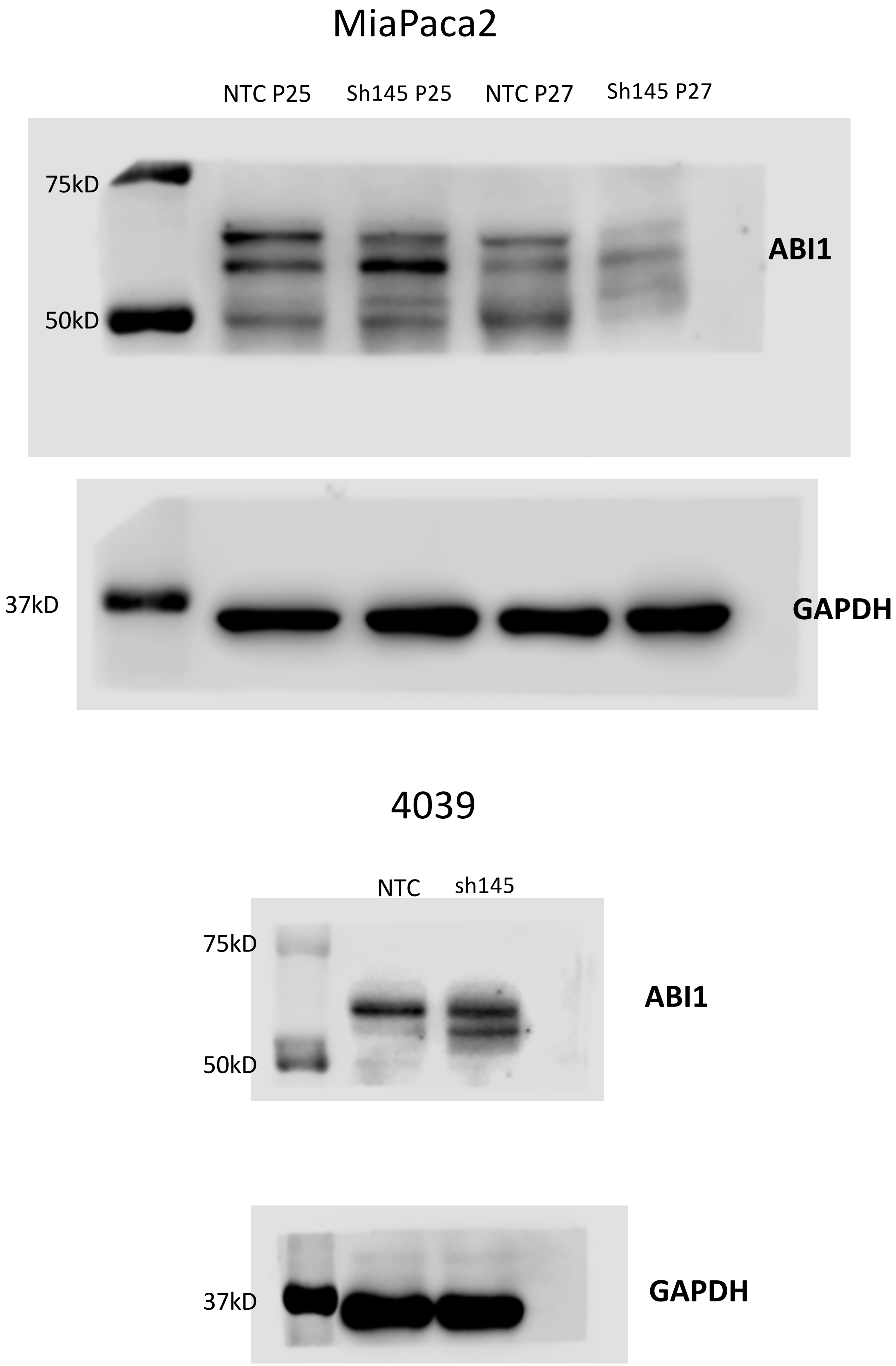

Supplemental Figure 12

Panel a/b

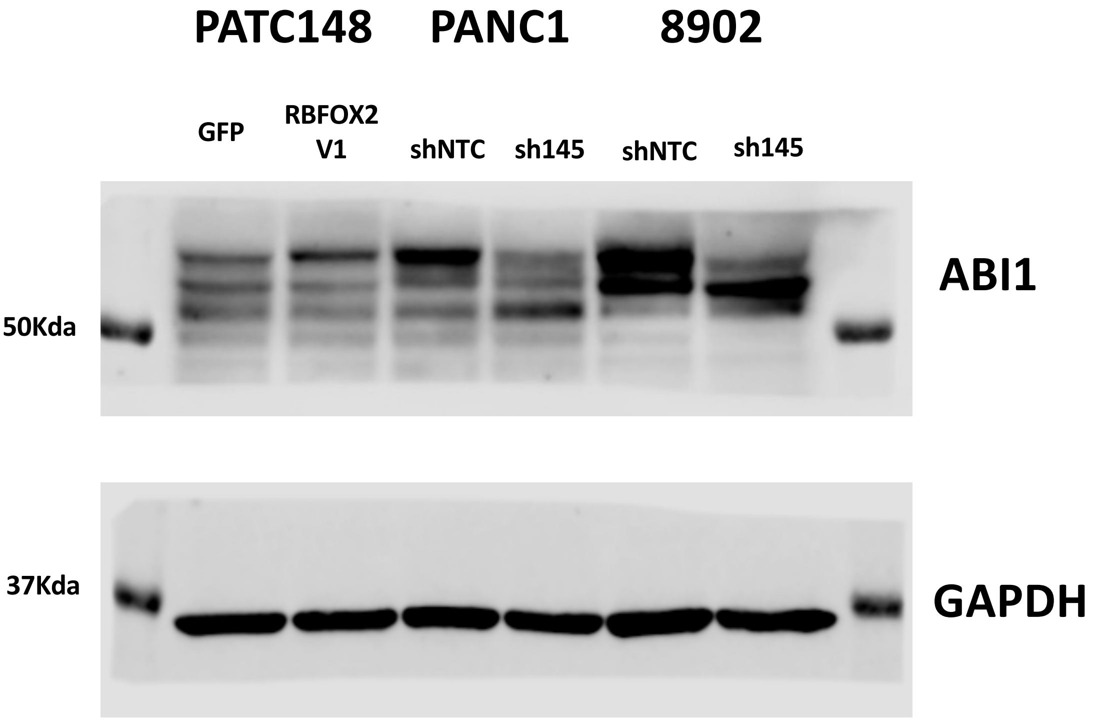

Panel c

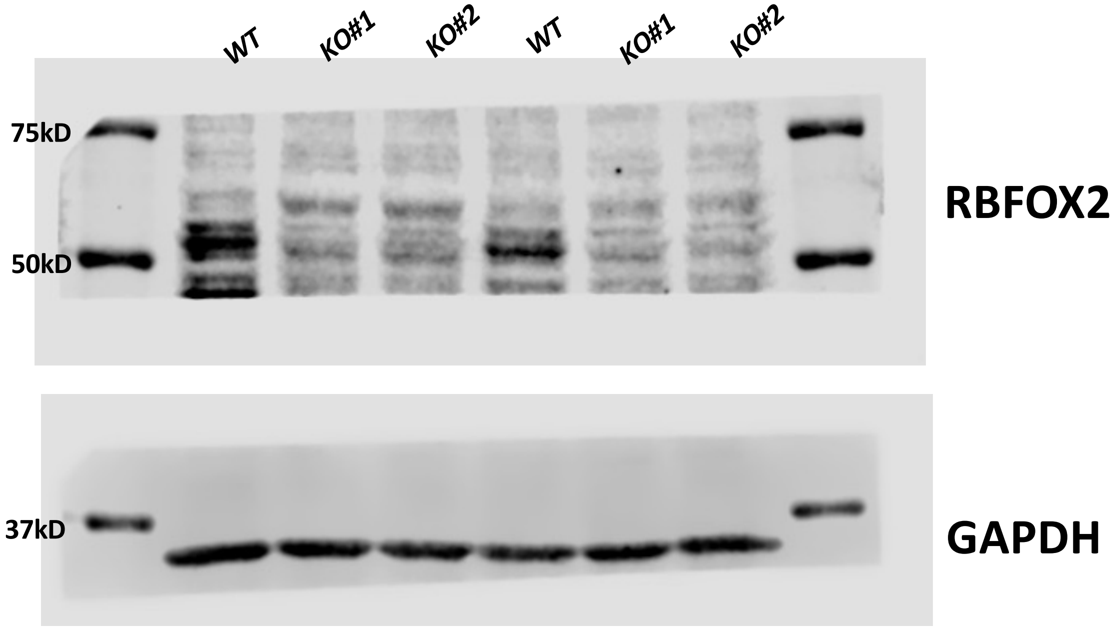

Supplemental Figure 12

Panel d

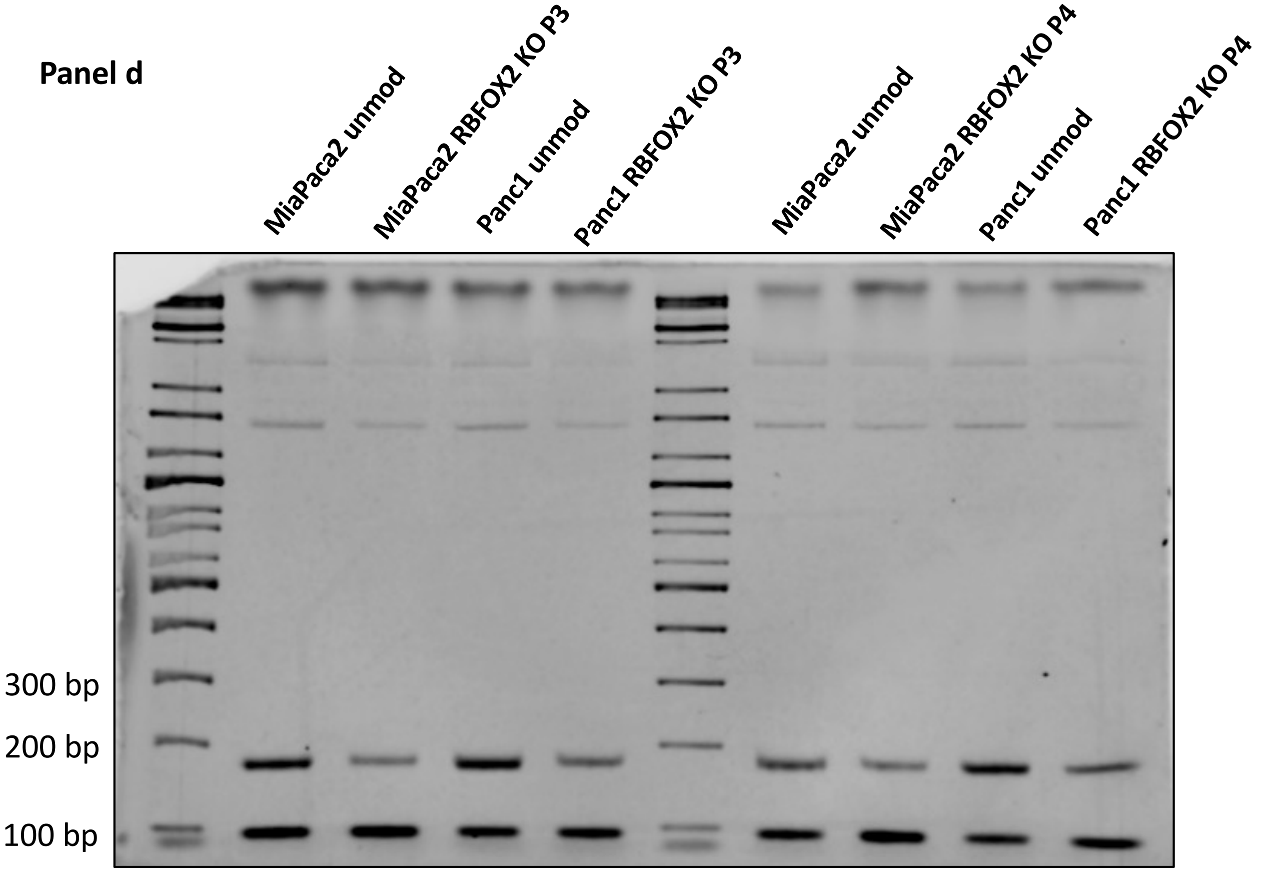

ABI1
Panel d

Panel f

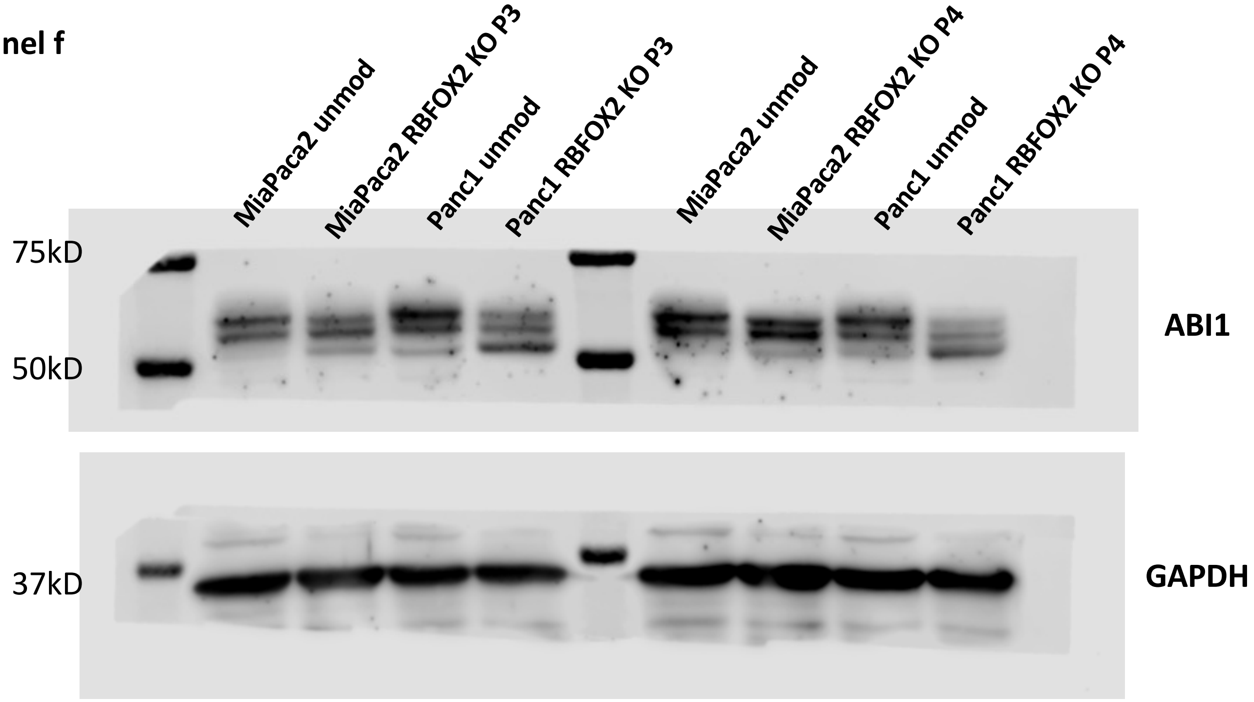

Supplement: Supplementary file 1 — Supplementary Information [file 41467_2023_44126_MOESM1_ESM.pdf]
