# Supplementary material for: RBFOX2 deregulation promotes pancreatic cancer progression and metastasis through alternative splicing
Source: Nat Commun. 2023 Dec 19;14:8444. doi: 10.1038/s41467-023-44126-w (PMC10730836; doi:10.1038/s41467-023-44126-w)
Supplement: Supplementary file 2 — Description of Additional Supplementary Files [file 41467_2023_44126_MOESM2_ESM.pdf]

Description of Additional Supplementary Files

File Name: Supplementary Data 1
Description: Clariom array output for RBFOX2 replete and depleted cell lines and tumors

File Name: Supplementary Data 2
Description: Correlation analysis of RBFOX2 TPM with target exon PSI and target gene TPM in human PDAC datasets
