# Supplementary material for: RBFOX2 deregulation promotes pancreatic cancer progression and metastasis through alternative splicing
Source: Nat Commun. 2023 Dec 19;14:8444. doi: 10.1038/s41467-023-44126-w (PMC10730836; doi:10.1038/s41467-023-44126-w)
Supplement: Supplementary file 5 — Reporting Summary [file 41467_2023_44126_MOESM5_ESM.pdf]

nature portfolio

Corresponding author(s): Karen M Mann, PhD

Last updated by author(s): 11/21/2023

Reporting Summary

Nature Portfolio wishes to improve the reproducibility of the work that we publish. This form provides structure for consistency and transparency in reporting. For further information on Nature Portfolio policies, see our Editorial Policies and the Editorial Policy Checklist.

Statistics

For all statistical analyses, confirm that the following items are present in the figure legend, table legend, main text, or Methods section.

n/a	Confirmed	
☐	☒	The exact sample size (*n*) for each experimental group/condition, given as a discrete number and unit of measurement
☐	☒	A statement on whether measurements were taken from distinct samples or whether the same sample was measured repeatedly
☐	☒	The statistical test(s) used AND whether they are one- or two-sided<br>*Only common tests should be described solely by name; describe more complex techniques in the Methods section.*
☒	☐	A description of all covariates tested
☐	☒	A description of any assumptions or corrections, such as tests of normality and adjustment for multiple comparisons
☐	☒	A full description of the statistical parameters including central tendency (e.g. means) or other basic estimates (e.g. regression coefficient) AND variation (e.g. standard deviation) or associated estimates of uncertainty (e.g. confidence intervals)
☐	☒	For null hypothesis testing, the test statistic (e.g. *F*, *t*, *r*) with confidence intervals, effect sizes, degrees of freedom and *P* value noted<br>*Give P values as exact values whenever suitable.*
☒	☐	For Bayesian analysis, information on the choice of priors and Markov chain Monte Carlo settings
☒	☐	For hierarchical and complex designs, identification of the appropriate level for tests and full reporting of outcomes
☐	☒	Estimates of effect sizes (e.g. Cohen's *d*, Pearson's *r*), indicating how they were calculated

Our web collection on statistics for biologists contains articles on many of the points above.

Software and code

Policy information about availability of computer code

Data collection	Proliferation assays were measured using the Promega Glomax Discover plate reader software version 3.2.3. Wound healing assays and chemotactic invasion assays were performed using the Incucyte SX5 with software version 2021A. Histological images were captured using the Zeiss Axio Imager.M2 with Zeiss Axiocam 503 color camera and Zeiss Zen Blue software version 3.5. TMAs were scanned using the Aperio™ ScanScope AT2 (Leica Biosystems, Vista, CA) with a 20x/0.8NA objective lens. Definiens Tissue Studio v4.7 (Definiens Inc, Germany) was used for segmentation and analysis of antibody staining. Immunofluorescence images were captured using the Leica TCS SP8 STED 3X and LAS X software. Western blot and splicing gel images were acquired using LI-COR Odyssey FC and accompanying software version 1.0.36. Human Affymetrix Clariom D Arrays were run on the Applied Biosystems GeneChip 3000 instrument with accompanying TACX software.
Data analysis	For wound healing assays, percent wound confluence was determined using Incycyte Zoom software version 2021A. For chemotactic invasion assays, percent invasion was calculated using the Integrated Incucyte® Chemotaxis Analysis Software Module (Sartorius) in Incucyte Zoom version 2021A. Positive pixel counts for nuclear and cytoplasmic staining of RBFOX2 in tumor cells from TMAs were determined using Definiens Tissue Studio v4.7 (Definiens Inc, Germany) for segmentation and analysis. In Tissue Studio, a machine learning algorithm was used to segment each TMA core image into Tumor and Non-Tumor (mostly Stroma) areas. A minimum size threshold setting was used to further refine this segmentation. A nucleus detection algorithm was used to find all nuclei within each segmented area. Detected objects that were smaller than 10 microns squared were not considered as nuclei. This nuclei detection was used for cell simulation using a simple growth algorithm of 5 microns. IHC stain intensity within the nucleus compartment was binned into four categories Negative, Low, Moderate, or High based on thresholds set using positive and negative stain controls included with this batch of slides. The data for each image was exported into Microsoft Excel where positive cell density, percent positive, and H-score were calculated using the raw data. Mean IHC staining intensity for the cell, nucleus, and cytoplasm compartments was also exported. Histological images were captured using the Zeiss Axio Imager.M2 with Zeiss Axiocam 503 color camera and Zeiss Zen Blue software version 3.5. Immunofluorescence images were quantified using ImageJ (version 1.53t) with a custom macro previously published (Bravo-Cordero, 2013). Protein and DNA bands were quantified using Image Studio Lite

software version 5.2.5. Differential exon usage analysis from generated .CEL files was analyzed using Affymetrix Transcriptome Analysis Console (TACX) software version 4.0.2 using the r1.GENE.CDF file with na36.hg38.a1 probesets. FASTQ data were aligned to the Human GRCh38 reference genome using STAR (version 2.7.3a). Gene-level and exon-level count data were generated using featureCounts from the Subread software package. Count data were converted to log2 counts per million, and processed in the R computing environment with the Voom normalisation procedure from the limma software package. Limma was also used for all differential expression analyses. The Python script dexseq_prepare_annotation.py provided with R package DEXSeq was used to translate Ensembl v84 GTF file to a GTF file with collapsed exon counting bin. The Percent Spliced In (PSI) index was calculated using a published protocol (doi: 10.1002/0471142905.hg1116s87). Pearson and Spearman correlation between RBFOX2 expression (TPM) and target exon PSI were calculated using R 4.0.3. Statistical analysis was carried out using R or GraphPad PRISM software version 10.2.0. Oncoprints were generated from published datasets from the Sleeping Beauty Cancer Driver Database (SBCDDB, https://sbcddb.moffitt.org/) using the cBioPortal Oncoprinter tool (https://www.cbioportal.org/oncoprinter).

For manuscripts utilizing custom algorithms or software that are central to the research but not yet described in published literature, software must be made available to editors and reviewers. We strongly encourage code deposition in a community repository (e.g. GitHub). See the Nature Portfolio guidelines for submitting code & software for further information.

Data

Policy information about availability of data

All manuscripts must include a data availability statement. This statement should provide the following information, where applicable:

- Accession codes, unique identifiers, or web links for publicly available datasets
- A description of any restrictions on data availability
- For clinical datasets or third party data, please ensure that the statement adheres to our policy

CEL files for Clariom D Exon Arrays generated in this study have been deposited in GEO under accession code GSE211435 (https://www.ncbi.nlm.nih.gov/geo/query/acc.cgi?acc=GSE211435). Microarray datasets for pancreatic tumors and normal pancreas were downloaded from GEO under accession code GSE16515 (https://www.ncbi.nlm.nih.gov/geo/query/acc.cgi?acc=gse16515) [80] and GSE28735 (https://www.ncbi.nlm.nih.gov/geo/query/acc.cgi?acc=GSE28735) [81]. RNA-seq data from patient samples are available at the European Genome Phenome Archive (https://www.ebi.ac.uk/ega) for dataset IDs EGAD00001003584 and EGAD00001004548 [1, 82-84]. CPTAC RNA-seq data from patient samples are available from Genomic Data Commons (https://gdc.cancer.gov) using dbGAP [33]. RNA-seq data from patient samples for dataset E-MTAB-6830 are available from the European Bioinformatics Institute (https://www.ebi.ac.uk) [34]. The Sleeping Beauty Cancer Driver database is accessible here: http://sbcddb.moffitt.org [22]. Source data are provided with this paper. High quality images for RBFOX2 IHC and confocal images can be found here https://doi.org/10.6084/m9.figshare.24212682. The remaining data are available within the Article, Supplementary Information or Source Data files.

Research involving human participants, their data, or biological material

Policy information about studies with human participants or human data. See also policy information about sex, gender (identity/presentation), and sexual orientation and race, ethnicity and racism.

Reporting on sex and gender	Analysis of RBFOX2 gene expression was performed using publicly available, de-identified human patient samples in the context of tumor vs. normal, or in the context of basal or classical subtypes as defined by previously published gene expression signatures. Analysis of alternative splice forms of RBFOX2 was performed in the context of basal or classical subtypes. Sex was not considered as a variable for these molecular signatures. Analysis of RBFOX2 protein abundance in de-identified human pancreatic tumors was analyzed in the context of histologic stage. No gender-specific differences were detected and data from both sexes was combined.
Reporting on race, ethnicity, or other socially relevant groupings	All samples were de-identified. Available population characteristics for gene expression datasets and/or TMAs were limited to sex, diagnosis, histologic grade and/or stage and overall survival.
Population characteristics	All samples were de-identified. Available population characteristics for gene expression datasets and/or TMAs were limited to sex, diagnosis, histologic grade and/or stage and overall survival.
Recruitment	No recruitment was conducted by the authors. All analyzed gene expression datasets were either publicly available or available through MTA. De-identified tissue microarrays were made available for general use by Moffitt Cancer Center.
Ethics oversight	Pancreatic cancer TMAs were previously constructed at Moffitt Cancer Center under Moffitt's Total Cancer Care (TCC), an institutional review board (IRB)-approved general biobanking protocol (MCC14690/IRB 104189 and MCC13579/IRB 101642). Under TCC, patients provide prospective written consent for biospecimen collection. Access to de-identified pancreatic cancer TMAs used in this study was granted under MCC50295.

Note that full information on the approval of the study protocol must also be provided in the manuscript.

Field-specific reporting

Please select the one below that is the best fit for your research. If you are not sure, read the appropriate sections before making your selection.

[x] Life sciences [ ] Behavioural & social sciences [ ] Ecological, evolutionary & environmental sciences

For a reference copy of the document with all sections, see nature.com/documents/nr-reporting-summary-flat.pdf

Life sciences study design

All studies must disclose on these points even when the disclosure is negative.

Sample size	The sample size for mice was determined using power calculations based on preliminary experiments using unmodified cell lines in orthotopic models to determine a 50% difference in the mean tumor volume between RBFOX2 replete and depleted models with 97% confidence at a significance of 0.05. For in vivo analysis of tumor growth and metastasis incidence in PDAC cells modified for RBFOX2 expression, 10-12 mice of both sexes were randomized into two groups, control and modified, for orthotopic implantation of tumor cells. For quantification of ABI1 and F-actin foci, a minimum of 30 cells were scored based on published criteria (www.jcb.org/cgi/doi/10.1083/jcb.201407082).
Data exclusions	Data exclusion criteria for quantification of tumor volumes in mice was based on whether a single data point was 2 standard deviations above or below the population mean. No data points were excluded based on these criteria. For scoring pancreatic TMAs for RBFOX2 expression, an individual core sample was excluded if there were fewer than 100 nuclei scored. Only two cores were excluded based on these criteria. Histology scores for individual samples that were outside 2 standard deviations above or below the population mean were excluded from statistical analysis. For RNA-seq analysis of gene expression or splicing in normal pancreas or PDAC samples, data exclusion criteria was based on whether a single data point was 2 standard deviations above or below the population mean. The number of samples per statistical comparison is indicated in the figures and/or figure legends. For cell based assays, technical replicates were excluded if the cells in the individual well failed to migrate or lifted off due to a poor scratch.
Replication	For all western blots, a minimum of three blots was assayed for quantification of protein levels. For cell proliferation, cell migration and cell invasion assays, 6-8 technical replicates per sample were assayed per experiment. Experiments were repeated a minimum of 3 times over different cellular passages to successfully replicate findings. Clariom D Arrays were performed for 3 independent cell line pairs in duplicate and analyzed at the population level. Four tumors replete or depleted for RBFOX2 were analyzed for differential splicing and gene expression independently of cell line data. Splicing PCRs were performed a minimum of 3 times over different cellular passages in 3 independent cell line pairs replete or depleted for RBFOX2 to successfully replicate array findings. Immunocytochemistry was performed in a minimum of 3 independent experiments for image capture and a minimum of 30 cells were analyzed. In vivo orthotopic experiments were successfully replicated in two or three independent experiments using two cell line pairs replete and depleted for RBFOX2.
Randomization	For in vivo orthotopic experiments, age-matched male and female NSG or C57Bl/6 mice were randomized into control and experimental groups. Randomization for other experiments were not subjective.
Blinding	Investigators were blinded for group designations for quantification of RBFOX2 in pancreatic TMAs. Blinding was performed for necropsies of control and experimental mice. No other blinding was performed as the output parameters were not subjective.

Reporting for specific materials, systems and methods

We require information from authors about some types of materials, experimental systems and methods used in many studies. Here, indicate whether each material, system or method listed is relevant to your study. If you are not sure if a list item applies to your research, read the appropriate section before selecting a response.

Materials & experimental systems

n/a	Involved in the study	
☐	☒	Antibodies
☐	☒	Eukaryotic cell lines
☒	☐	Palaeontology and archaeology
☐	☒	Animals and other organisms
☒	☐	Clinical data
☒	☐	Dual use research of concern
☒	☐	Plants

Methods

n/a	Involved in the study	
☒	☐	ChIP-seq
☒	☐	Flow cytometry
☒	☐	MRI-based neuroimaging

Antibodies

Antibodies used	The following antibodies were used fro immunoblotting in this study: : RBFOX2 ((RBM9) Bethyl Labs, Cat. No. A300-864A, 1:2000); ABI1 (Cat. no. 39444, 1:500), Vimentin (Cat. no. 5741S, 1:1000), CDH1(Cat. no. 3195S, 1:1000) and Rac1 (Cat. no. 4651, 1:500) from Cell Signaling; GAPDH (Santa Cruz Cat. no. SC-69778, 1:5000); Beta-Actin (Cat. no A5441, 1:20K), Flag-M2 (Cat. no. F3165, 1:5000) and Histone H3 (Cat. no. 05-928, 1:2000) from Sigma. LI-COR Secondary antibodies for Infrared were IRDye 800cw anti-Rabbit (Cat. no. 926-32211, 1:5000) and IRDye 680RD anti-Mouse (Cat. no. 925-68070, 1:5000); HRP-conjugated anti-Rabbit (Vector Laboratories, Cat. no. PI-1000, 1:5000) and HRP-conjugated anti-Mouse (Jackson Immuno Research Labs, Cat. no. 115-035-003, 1:5000).<br><br>The following antibodies were used for immunohistochemistry and immunofluorescence: RBFOX2 antibody (RBM9 IHC00199, Bethyl Labs, 1:600 for mouse tissue and 1:2000 for human tissue); Alexa Fluor 594-labelled phalloidin (ThermoFisher, Cat. no. A12381, 1:1200); ABI1 antibody (Invitrogen PA5110991, 1:250) with Alexa-488 conjugated secondary (Invitrogen A11034, 1:1000). Cortactin ab [4F11] (Abcam, Cat. no. ab33333, 1:250) with Alexa-546 conjugated secondary (Invitrogen A10040, 1:1000).
Validation	Antibodies validation was performed as follows: for immunohistochemistry, no primary controls and positive tissue (pancreas and spleen for mouse and breast tissue for human samples; for immunocytochemistry, no primary controls and positive cell line MB-MDA-231 for ABI1 (10.1093/carcin/bgp251) and phalloidin. For western blotting,antibodies were used following the manufacturer's

	recommendations. MCF7 cells were used as a positive control for RBFOX2 (10.1093/narcan/zcaa021) and MB-MDA-231 cells for ABI1 (10.1093/carcin/bgp251) expression in addition to PDAC cell lines with RBFOX2 and ABI1 knockdown or overexpression for protein target validation.

Eukaryotic cell lines

Policy information about cell lines and Sex and Gender in Research

Cell line source(s)	The following cell lines were obtained from ATCC: 4039 (CRL-4039); Panc1 (CRL-1469); MiaPaCa2 (CRL-1420); PL45 (CRL-2558); Panc02.03 (CRL-2553); BxPC3 (CRL-1687); HPNE (CRL-4023), 293FT. MDA-PATC cell lines (PATC124, PATC43, PATC153, PATC107, PATC53, PATC102, PATC108, PATC148) were derived from PDAC PDX models (DOIs 10.1158/1078-0432.CCR-15-2936 and 10.1158/1078-0432.CCR-14-0970) and were obtained from MD Anderson Cancer Center. 8902 cells were obtained from the DSMZ-German Collection of Microorganisms and Cell Cultures GmbH. KPC cell line K1242 (male) was obtained from Dr. Dave Tuveson (CSHL).
Authentication	Cell lines were validated using Sort Tandem Repeat (STR) profiling and mutational analysis using Sanger Sequencing for KRAS and P53 for PATC lines.
Mycoplasma contamination	Cell lines were routinely tested to confirm their mycoplasma-negative status using the PCR-based SIGMA Venor GeM Mycoplasma Detection kit.
Commonly misidentified lines (See ICLAC register)	No commonly mis-identified lines were used in the study.

Animals and other research organisms

Policy information about studies involving animals; ARRIVE guidelines recommended for reporting animal research, and Sex and Gender in Research

Laboratory animals	Male and female NSG recipient mice (JAX 005557) or C57Bl/6J mice (JAX 000664) ages 8 to 16 weeks were utilized in this study. Mice were maintained on a 12:12 light/dark cycle, with food and water ad libitum.
Wild animals	This study did not involve wild animals
Reporting on sex	Age-matched male and female mice were randomized into control and experimental groups. Data from both sexes was analyzed together as there was no significant differences observed between the sexes.
Field-collected samples	This study does not use samples collected in the field
Ethics oversight	All procedures using mice were conducted under approved IACUC protocols and following AALAC guidelines.

Note that full information on the approval of the study protocol must also be provided in the manuscript.
